# BCG-induced non-specific effects on heterologous infectious disease in Ugandan neonates: an investigator-blind randomised controlled trial

**DOI:** 10.1016/S1473-3099(20)30653-8

**Published:** 2021-07

**Authors:** Sarah Prentice, Beatrice Nassanga, Emily L Webb, Florence Akello, Fred Kiwudhu, Hellen Akurut, Alison M Elliott, Rob J W Arts, Mihai G Netea, Hazel M Dockrell, Stephen Cose, The Delayed BCG Study Team, Sarah Prentice, Beatrice Nassanga, Hellen Akurut, Florence Akello, Fred Kiwudhu, Stephen Cose, Hazel Dockrell, Emily Webb, Alison Elliott, Irene Nabaweesi, Christopher Zziwa, Milly Namutebi, Benigna Namarra, Florence Akello, Esther Nakazibwe, Susan Amongi, Grace Kamukama, Susan Iwala, Caroline Ninsiima, Josephine Tumusiime, Fred Kiwanuka, Saadn Nsubuga, Justin Akello, Sebastian Owilla, Jonathan Levin, Stephen Nash, Prossy Kabuubi Nakawungu, Elson Abayo, Grace Nabakooza, Zephyrian Kaushaaga, Miriam Akello

**Affiliations:** a Clinical Research Department, London School of Hygiene and Tropical Medicine, London, UK; b Department of Infectious Disease Epidemiology, London School of Hygiene and Tropical Medicine, London, UK; c Department of Infection Biology, London School of Hygiene and Tropical Medicine, London, UK; d MRC/UVRI and LSHTM Uganda Research Unit, Entebbe, Uganda; e Department of Internal Medicine and Radboud Centre for Infectious Disease, Radboud University Medical Centre, Nijmegen, Netherlands; f Department for Genomics and Immunoregulation, Life and Medical Sciences Institute, University of Bonn, Bonn, Germany

## Abstract

**Background:**

Trials done in infants with low birthweight in west Africa suggest that BCG vaccination reduces all-cause mortality in the neonatal period, probably because of heterologous protection against non-tuberculous infections. This study investigated whether BCG alters all-cause infectious disease morbidity in healthy infants in a different high-mortality setting, and explored whether the changes are mediated via trained innate immunity.

**Methods:**

This was an investigator-blind, randomised, controlled trial done at one hospital in Entebbe, Uganda. Infants who were born unwell (ie, those who were not well enough to be discharged directly home from the labour ward because they required medical intervention), with major congenital malformations, to mothers with HIV, into families with known or suspected tuberculosis, or for whom cord blood samples could not be taken, were excluded from the study. Any other infant well enough to be discharged directly from the labour ward was eligible for inclusion, with no limitation on gestational age or birthweight. Participants were recruited at birth and randomly assigned (1:1) to receive standard dose BCG 1331 (BCG-Danish) on the day of birth or at age 6 weeks (computer-generated randomisation, block sizes of 24, stratified by sex). Investigators and clinicians were masked to group assignment; parents were not masked. Participants were clinically followed up to age 10 weeks and contributed blood samples to one of three immunological substudies. The primary clinical outcome was physician-diagnosed non-tuberculous infectious disease incidence. Primary immunological outcomes were histone trimethylation at the promoter region of *TNF, IL6*, and *IL1B; ex-vivo* production of TNF, IL-6, IL-1β, IL-10, and IFNγ after heterologous stimulation; and transferrin saturation and hepcidin levels. All outcomes were analysed in the modified intention-to-treat population of all randomly assigned participants except those whose for whom consent was withdrawn. This trial is registered with the International Standard Randomised Controlled Trial Number registry (#59683017).

**Findings:**

Between Sept 25, 2014, and July 31, 2015, 560 participants were enrolled and randomly assigned to receive BCG at birth (n=280) or age 6 weeks (n=280). 12 participants assigned to receive BCG at birth and 11 participants assigned to receive BCG at age 6 weeks were withdrawn from the study by their parents shortly after randomisation and were not included in analyses. During the first 6 weeks of life before the infants in the delayed vaccination group received BCG vaccination, physician-diagnosed non-tuberculous infectious disease incidence was lower in infants in the BCG at birth group than in the delayed group (98 presentations in the BCG at birth group *vs* 129 in the delayed BCG group; hazard ratio [HR] 0·71 [95% CI 0·53–0·95], p=0·023). After BCG in the delayed group (ie, during the age 6–10 weeks follow-up), there was no significant difference in non-tuberculous infectious disease incidence between the groups (88 presentations *vs* 76 presentations; HR 1·10 [0·87–1·40], p=0·62). BCG at birth inhibited the increase in histone trimethylation at the *TNF* promoter in peripheral blood mononuclear cells occurring in the first 6 weeks of life. H3K4me3 geometric mean fold-increases were 3·1 times lower at the *TNF* promoter (p=0·018), 2·5 times lower at the *IL6* promoter (p=0·20), and 3·1 times lower at the *IL1B* promoter (p=0·082) and H3K9me3 geometric mean fold-increases were 8·9 times lower at the *TNF* promoter (p=0·0046), 1·2 times lower at the *IL6* promoter (p=0·75), and 4·6 times lower at the *IL1B* promoter (p=0·068), in BCG-vaccinated (BCG at birth group) versus BCG-naive (delayed BCG group) infants. No clear effect of BCG on ex-vivo production of TNF, IL-6, IL-1β, IL-10, and IFNγ after heterologous stimulation, or transferrin saturation and hepcidin concentration, was detected (geometric mean ratios between 0·68 and 1·68; p≥0·038 for all comparisons).

**Interpretation:**

BCG vaccination protects against non-tuberculous infectious disease during the neonatal period, in addition to having tuberculosis-specific effects. Prioritisation of BCG on the first day of life in high-mortality settings might have significant public-health benefits through reductions in all-cause infectious morbidity and mortality.

**Funding:**

Wellcome Trust.

**Translations:**

For the Luganda and Swahili translations of the abstract see Supplementary Materials section.

**Research in context**

**Evidence before this study**

In 2016, a systematic review of the non-specific effects of vaccinations in childhood identified five trials and 13 observational studies investigating the effect of BCG on all-cause mortality, before March 2013. The trials had a low to moderate risk of bias. Combined analysis of the trials suggested a beneficial effect of BCG on all-cause mortality with an average relative risk of 0·70 (95% CI 0·49–1·01). Mortality reduction was most significant in two trials that were restricted to infants with low birthweight (average relative risk 0·52 [0·33–0·82]).

We searched PubMed for articles published in any language from March 1, 2013, to March 31, 2020, using the medical subject headings ”BCG”, in abbreviated and long form, and ”non-specific” or “heterologous” or ”off-target” and their variations. This search identified a further three randomised controlled trials and ten observational studies investigating the effect of BCG on childhood mortality and infectious disease morbidity. Two of the three RCTs were conducted in infants with low birthweight. None of the trials reported statistically significant reductions in all-cause mortality or hospital admissions in BCG-vaccinated infants. Seven of ten of the observational studies did report reductions in all-cause mortality or infectious disease incidence (particularly lower respiratory tract infections and sepsis) associated with BCG, but all had substantial risks of bias.

Taken together, the evidence for BCG-induced protection against heterologous infectious disease before this trial remained contentious. Although tending to support there being beneficial non-specific effects of BCG in neonates, the evidence was limited by a large number of reports from the same study group in west Africa, positive data being dominated by studies in infants with low birthweight, and that most of the trials assessed all-cause mortality rather than non-tuberculous infectious disease incidence.

**Added value of this study**

This study shows a significant reduction in physician-diagnosed all-cause infectious disease incidence in the first 6 weeks of life in infants randomly assigned to receive BCG at birth compared with those who had not yet received the vaccine. It is the first RCT to assess the non-specific effects of BCG on total infectious disease morbidity. The close clinical follow-up of participants and access to enhanced investigations allowed accurate assessment of non-tuberculous infectious disease, rather than relying on the proxy measures of all-cause mortality or hospital admission rates. By performing the trial in east Africa (Uganda), and enrolling infants with normal birthweight and low birthweight, the study also provides more generalisable estimates of the non-specific effects of BCG in neonatal populations. The findings of the study corroborate previous observations that the non-specific benefits of BCG are more pronounced in infants with low birthweight and boys.

This study is also the first RCT in neonates to assess the potential for the BCG to train the innate immune system. We found that BCG at birth is associated with changes to histone trimethylation at some proinflammatory cytokine promoters in peripheral blood mononuclear cells.

**Implications of all the available evidence**

The evidence supports the WHO policy of providing BCG vaccination to all infants on the first day of life in areas of high infectious disease incidence, for reductions in neonatal all-cause infection-related morbidity and mortality as well as tuberculosis-specific protection. Strengthening existing programmes to ensure timely BCG vaccination would be a low cost, easily implementable intervention with public health benefits beyond protection against tuberculosis. Trials assessing the reintroduction of neonatal BCG vaccination in low tuberculosis-incidence settings, to provide non-specific protection against SARS-CoV2, should be strongly considered.

## Introduction

Infections cause nearly 1 million neonatal deaths annually, most of which are in low-income and middle-income countries.^1^ With increasing antimicrobial resistance worldwide and the emergence of novel pathogens such as severe acute respiratory syndrome coronavirus 2 (SARS-CoV-2), improved strategies to enhance neonatal resistance to infection are urgently needed.^2^ The possibility that BCG vaccination protects against non-tuberculous infectious disease in the neonatal period has been suggested from animal models,^3^ human challenge models,^4^, ^5^ observational studies,^6^ and several randomised controlled trials.^7^, ^8^, ^9^, ^10^ Reviewing the immunological^11^ and clinical^12^ evidence in 2016 (from studies published up to January, 2014), the Strategic Advisory Group of Experts for WHO concluded that BCG-induced non-specific beneficial effects might exist, but that the nature, magnitude, timing, and clinical importance of such effects were unclear.^11^ Concerns were raised regarding the quality of the evidence, with many studies at high risk of bias,^13^ and regarding the generalisability of findings, because randomised controlled trial data were only available for infants with low birthweight in west Africa.^7^, ^8^, ^9^, ^10^ No conclusive biological mechanism to explain BCG-induced non-specific effects in neonates was identified. Experiments in Dutch adults show that BCG epigenetically reprogrammes the innate immune system, leading to enhanced responses upon heterologous pathogen challenge,^5^ but such studies have not been done in neonates.

This study was designed to explore whether BCG produces non-specific protection against heterologous infectious disease in healthy Ugandan neonates, and whether the protection is mediated by alterations to innate immunity. In this trial we aimed to accurately identify total infectious disease morbidity by prospective, close, clinical follow-up, rather than relying on the proxy measures of all-cause mortality or hospital admission rates as in previous studies. We further aimed to investigate the potential for BCG to train the innate immune system in neonates.

## Methods

### Study design and participants

We did an investigator-masked, randomised controlled trial comparing early (at birth) versus delayed (age 6 weeks) BCG vaccination in Ugandan neonates at Entebbe Grade B District Hospital in Wakiso District, Uganda, which serves urban and nearby rural communities.

Ugandan neonates were recruited at birth. Infants residing outside the study area, born unwell (ie, those who were not well enough to be discharged directly home from the labour ward because they required medical intervention), with major congenital malformations, to HIV seropositive mothers, or into a household with known or suspected tuberculous disease were excluded. Any other infant well enough to be discharged directly from hospital was eligible for inclusion, with no minimum gestational age or weight, reflecting Ugandan BCG vaccination policy.

This study was approved by the London School of Hygiene and Tropical Medicine (#6545), the Uganda Virus Research Institute (#GC/127/13/11/432), the Ugandan National Drug Authority (#382/ESR/NDA/DID-10/2014), the Uganda National Council for Science and Technology (#HS1524), and the Office of the President of Uganda, and conducted according to Declaration of Helsinki principles. Written, informed consent was taken from participant's mothers, and fathers where possible, before enrolment. The study protocol is in the appendix 3 (pp 22–70) and is published.^14^

### Randomisation and masking

Participants were randomly assigned 1:1 to receive BCG either at birth (early vaccination group) or at age 6 weeks (delayed vaccination group). An independent statistician generated randomisation lists using STATA, version 13.1, in blocks of 24, stratified by sex. Randomisation allocations were concealed within numbered opaque envelopes, prepared by research technicians not otherwise involved in the study. Parents were invited to select between the next two sequentially ordered envelopes, according to the infant's sex, to establish final allocation, with the unselected envelope returned to be used in the next randomisation. This provided a visual reinforcement of randomisation, helping to reassure parents that the study team did not select the timing of BCG administration. Randomisation codes were held by an independent statistician and accessed after follow-up was complete and data cleaned and locked.

Investigators and clinicians were masked to assignment. No placebo vaccination was used; parents were not masked to vaccination status, because BCG produces a visible reaction that would be difficult to conceal, and to ensure that unvaccinated participants lost to follow-up would receive BCG in the community. An alternative vaccination was not used to avoid confounding heterologous effects.

To maintain investigator masking, a non-clinical team member placed a plaster over the expected vaccination site and sealed the vaccination cards in an opaque envelope, upon a participant's presentation to the clinic. Parents were asked not to discuss their child's BCG status with clinicians, unless they were specifically concerned about the vaccination site, in which case unmasking was documented and the clinical episode removed from analysis. Recruitment, randomisation, and intervention administration was done by nurses not involved in clinical assessment of participants, in a separate location. Laboratory technicians were masked to intervention allocation, with samples processed using anonymised laboratory numbers.

### Procedures

Participants were assigned to receive BCG 1331/Danish (Statens Serum Institute, Denmark) either at birth (age <24 h) or at age 6 weeks (±6 days) at the standard neonatal dose, 0·05 mL intradermally to the right deltoid. A single batch of BCG 1331 (113033c) was used, to achieve consistency with most previous studies on non-specific effects, and because of concerns regarding strain or batch variability.^15^, ^16^, ^17^ Participants were actively followed-up until age 10 weeks for illness outcomes. Blood samples were collected at four timepoints to investigate the effect of BCG on three aspects of innate immunity: epigenetic modification of peripheral blood mononuclear cells (PBMCs), innate cytokine production after ex-vivo stimulation with heterologous pathogens, and the inflammatory iron response.

Placental cord blood was collected at delivery. After randomisation, participants allocated to BCG at birth were vaccinated with BCG. All participants were given oral polio vaccine at birth as per the Ugandan Expanded Programme of Immunisations (EPI), and anthropometry and vital signs were measured.

Participants attended four routine follow-up clinic visits during their 10-week participation, for blood sampling or EPI vaccinations (diphtheria–tetanus-–whole-cell pertussis–*Haemophilus influenzae* B–hepatitis B, ten-valent pneumococcal conjugate vaccine, and oral polio vaccine at 6 weeks and 10 weeks) and for BCG in the delayed group at 6 weeks. Participants had open clinic access whenever parents deemed them unwell. At all clinic visits, anthropometry and vital signs were measured and physician review for current illnesses and history of interim illnesses were conducted.

Complete identification of illness episodes was ensured by open, cost-free access to treatment at the research clinic throughout follow-up, physician review at routine appointments, and weekly telephone interviews regarding the participant's health. A study physician attended Entebbe Hospital daily to identify participants presenting there directly. A simple verbal autopsy was performed upon the death of a participant. Diagnostics available at the research clinic included microscopy, cultures (blood, cerebrospinal fluid, swab, urine, stool, and sputum), HIV and malaria rapid diagnostic tests, haematology indices, basic biochemistry, and radiology. More advanced investigations were available at the referral hospital (Mulago Hospital, Kampala, Uganda) if required. A presumptive diagnosis was made by the attending physician and reviewed later with test results. Diagnosis of the likely cause of interim illnesses without clinic presentation was made by the attending physician based on parental history. Because of the potential for duplication of illness events with multiple capture methods, all illness files were hand-searched by a masked senior physician (SP). Presentations with the same symptoms within 1 week of each other were classified as one episode and assigned the earliest date of symptoms as the date of diagnosis.

Participants had two 2-mL venous blood samples taken during the study, randomly selected from four timepoints: 5 days, 6 weeks (immediately before BCG in the delayed group), 6 weeks (5 days after BCG in the delayed group), and 10 weeks. These timepoints allowed exploration of the short-term and longer-term effects of BCG comparing vaccinated and unvaccinated infants (5 days for short term and 6 weeks [before BCG] for longer term), and early and delayed BCG (6 weeks [5 days after BCG] for short term and 10 weeks for long-term). Blood samples contributed to three distinct immunological substudies, run sequentially. Histone-3, lysine-4 trimethylation (H3K4me3) and histone-3, lysine-9 trimethylation (H3K9me3) at the promoter region of *TNF, IL6,* and *IL1B* was assessed in PBMCs by ChIP-qPCR (epigenetic substudy). Ex-vivo production of TNF, IL-6, IL-1β, IL-10 and IFNγ after 24-h stimulation of whole blood with killed-whole pathogens (*Streptococcus pneumoniae, Staphylococcus aureus, Escherichia coli,* and *Candida albicans)*, the TLR agonist poly I:C (polyinosinic:poycytidylic acid) and a positive control (purified peptide derivative), was measured by ELISA (cytokine substudy). Components of the inflammatory iron axis were measured by automated analyser (iron and TSAT), or ELISA (hepcidin and IL-6) for the iron substudy. Funding constraints limited epigenetic substudy sample collection to timepoints 1 and 2, and sample analysis to baseline and 6 weeks. Because the volume of blood that can be taken from each neonate is small (2 mL), samples from each infant could only be used in one substudy. Detailed laboratory methods and the study procedures diagram are in the appendix 3 (pp 1–3).

### Outcomes

The primary clinical outcome for the trial was incidence of physician-diagnosed, non-tuberculous infectious disease. Secondary clinical outcomes were numbers of parental reports of infectious disease (for which no physician review occurred), blood culture-positive infectious disease rates, and mortality.

The primary immunological outcomes were between-group comparisons of histone trimethylation at the promoter region of *TNF, IL6*, and *IL1B* (epigenetic substudy); stimulated TNF, IL-6, IL-1β, IL-10, and IFNγ levels (cytokine substudy); and TSAT and hepcidin levels (iron substudy).

In the original ISRCTN documentation and published protocol,^14^ the coprimary outcome of physician-diagnosed non-tuberculous infectious disease was incorrectly listed as a secondary outcome. In the originally approved version of the study protocol (version 2.2, approval date Jan 2, 2014, before commencement of the study) and all subsequent versions, it was listed as a coprimary outcome, and was thus not a post-hoc change. This erratum in documentation has been corrected.

### Statistical analysis

Based on a previous study done in Entebbe, which showed a physician-diagnosed infection incidence of 680 per 1000 infants during the first 10 weeks of life and serious illness incidence of 223 per 1000,^18^ the 560 recruited infants provided 80% power to detect an at least 25% reduction in all-cause infectious illness, or a minimum of 40% reduction in serious illness, with p<0·05. This reduction in serious illness would be of similar magnitude to the mortality rate reduction reported in the systematic review of clinical evidence.^12^ Sample size calculations and rationale for the immunological substudies are in the study protocol (appendix 3 pp 40–41).

Baseline group characteristics were compared using Pearson's χ^2^ test for categorical variables and the *t* test for continuous variables. Rates of clinical outcomes by BCG group were compared using Poisson regression with robust standard errors, to allow for within-child clustering. Parental reports of interim infection episodes by group were compared using logistic regression with robust standard errors. The main time period of interest was the first 6 weeks of life, when the two groups differed by BCG vaccination status (BCG vaccinated *vs* unvaccinated). The effect of delaying BCG on infectious illness events was also analysed by comparing illness rates after the delayed group had received their BCG (6–10 weeks of age) and over the follow-up period as a whole.

Between-group comparisons of immunological outcomes at each timepoint were conducted using linear regression, with logarithmic transformation of non-normally distributed data. The Mann-Whitney two-tailed test was used for persistently skewed data. Results below the limit of detection were assigned a value half the square root of the lowest detectable value. For stimulated cytokine data, the log-transformed value of the unstimulated cytokine response was included as a co-variate to account for individual variability. Immunological results are reported without correction for multiple comparisons to maximise identification of trends.

Analyses were conducted by sex and birthweight, with tests for interaction reported. Recruitment was stratified by sex and infants of all birthweights were eligible, to allow for these exploratory subgroup analyses.^7^ All analyses were done in both the modified intention-to-treat population (all randomly assigned participants except those for whom consent was withdrawn) and per-protocol population. Modified intention-to-treat results are reported here. Statistical significance was assessed at the two-sided 0·05 level, but interpretation of results was not solely reliant on p values. Stata, version 14.1, was used for statistical analysis. Graphs were produced using GraphPad Prism, version 6.0. An independent data safety monitoring board reviewed the trial twice during its course, with no recommendations for early cessation. This trial is registered with the International Standard Randomised Controlled Trial Number registry (#59683017).

### Role of the funding source

The funders had no role in study design, data collection, data analysis, data interpretation, or writing of the report.

## Results

**Figure 1:**
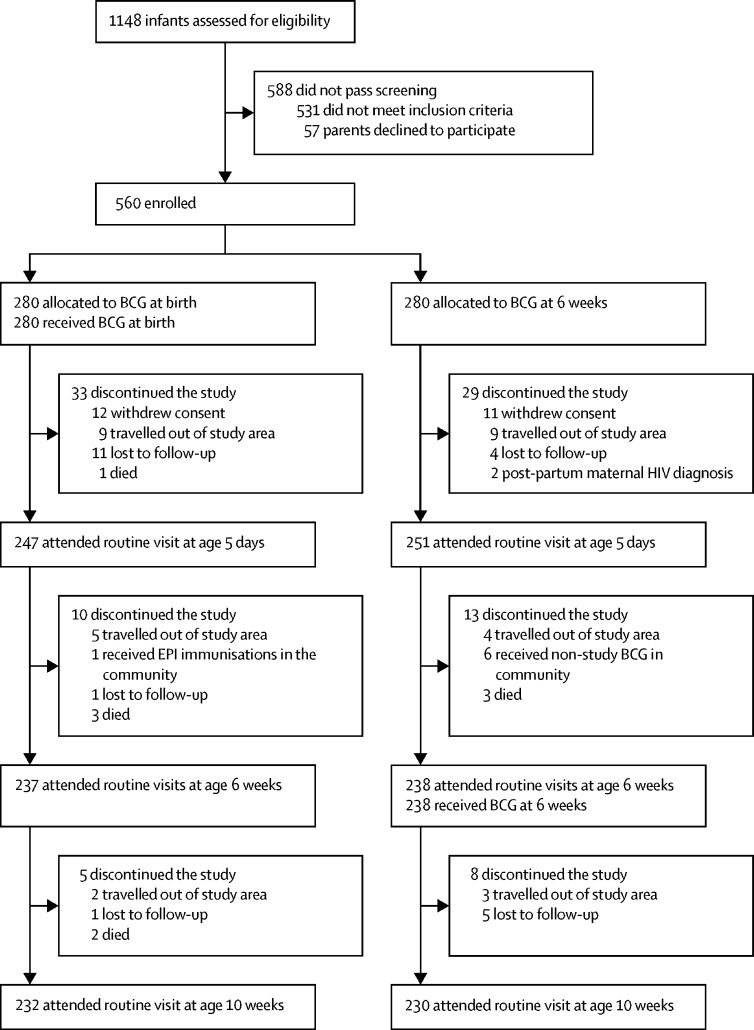
Trial profile Trial profiles for the individual immunological substudies, including total blood sample availability at each timepoint, are in the appendix 3 (pp 5–7). EPI=Expanded Programme of Immunisations.

Between Sept 25, 2014, and July 31, 2015, 1148 Ugandan neonates were screened, of whom 560 were enrolled and randomly assigned to receive BCG at birth (n=280) or at age 6 weeks (n=280). Of 560 recruited participants, 462 (83%) completed the full 10-week study period (figure 1). 12 participants assigned to receive BCG at birth and 11 participants assigned to receive BCG at age 6 weeks were withdrawn from the study by their parents shortly after randomisation and were not included in analyses. Participant characteristics and reasons for study non-completion were similar between groups (table 1, figure 1). Unmasking occurred on 11 occasions, eight due to vaccination site reactions and three accidentally.

**Table 1:** Baseline characteristics of enrolled participants

		**BCG at birth (n=268)**	**BCG at 6 weeks (n=269)**
Sex			
	Male	134 (50%)	137 (51%)
	Female	134 (50%)	132 (49%)
Birthweight, g		3239·1 (410·7)	3199·0 (450·8)
Low birthweight (≤2500 g)		13 (5%)	16 (6%)
Occipitofrontal circumference, cm		34·5 (1·4)	34·5 (1·5)
Maternal age, years		24 (4·6)	24 (4·7)
Marital status			
	Married or living as married	233 (87%)	224 (83%)
	Single	34 (13%)	42 (16%)
Parity		2 (1–3)	2 (1–3)
Number of people in house		4 (3–5)	4 (3–5)
Maternal education			
	None	5 (2%)	8 (3%)
	Primary	89 (33%)	87 (32%)
	Secondary	142 (53%)	149 (55%)
	Tertiary	32 (12%)	25 (9%)
Iron supplements during pregnancy			
	Yes	245 (91%)	245 (91%)
	No	23 (9%)	24 (9%)
Maternal smoking in pregnancy			
	Yes	0	1 (<1%)
	No	268 (100%)	268 (>99%)
Maternal alcohol in pregnancy			
	Yes	39 (15%)	33 (12%)
	No	229 (85%)	236 (88%)

Data are n (%), mean (SD), and median (IQR). Participants who withdrew are not included in the table.

616 illness events occurred, comprising 470 presentations to clinic and 146 parental reports of interim illness with no presentation. Most of these events were deemed infectious in origin. Presentations for non-infectious reasons included 56 for common, benign, infant conditions such as physiological jaundice and 22 where the infant was deemed well. 16 infants were admitted to hospital during the study and eight died. One infant was found to have died 2 days after withdrawal from the study. Inclusion of this death in statistical analysis made little difference to the results. Deaths are not included in the analyses presented here but are described in the appendix 3 (p 4).

**Figure 2:**
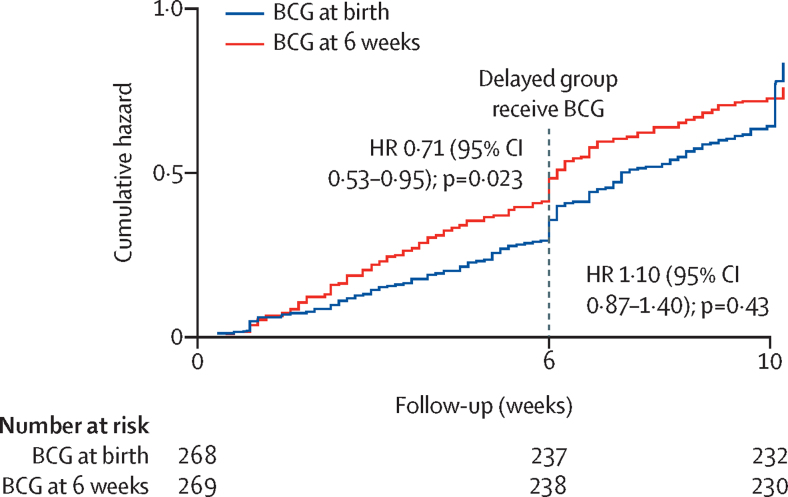
Cumulative hazard of physician-diagnosed, non-tuberculous infectious disease The observed step-in at 6 weeks and 10 weeks in both groups is probably a function of the study design. Routine clinic appointments were scheduled at 6 weeks and at 10 weeks, so parents of infants who were mild to moderately unwell in the few days preceding a clinic visit might have deferred attendance for convenience, leading to an artificial observed increase in illness rates at each of the clinic days.

In the period before BCG vaccination in the delayed group, the incidence of physician-diagnosed non-tuberculous infectious disease was significantly lower in infants who received the BCG vaccine at birth (98 presentations; cumulative incidence 36 per 100) than in those vaccinated at age 6 weeks (129 presentations; cumulative incidence 48 per 100; HR 0·71 [95% CI 0·53–0·95], p=0·023; figure 2, table 2). No clear differences in rates of parental reports of interim infections, culture-positive infections, or mortality were seen (appendix 3 p 9). The types of infectious disease diagnosed by physicians during follow-up did not differ according to BCG timing (appendix 3 p 10), with no evidence for a decrease in specific illnesses such as neonatal sepsis or lower respiratory tract infections. Numbers of presentations with serious illness, as defined by the WHO Integrated Management of Childhood Illness, were somewhat lower during the first 6 weeks of life in infants vaccinated at birth with BCG than in those in the delayed group who had not yet received the vaccine, although the difference was not significant (HR 0·68 [0·43–1·07], p=0·093; table 2),

**Table 2:** Clinical outcome events comparing infants BCG-vaccinated at birth with infants BCG-vaccinated at age 6 weeks, overall and by sex and birthweight

	Before delayed BCG (age 0–6 weeks)*				**After delayed BCG (age 6–10 weeks)**†				**Total follow-up period**			
	Frequency in BCG at birth group	Frequency in BCG at 6 weeks group	Hazard ratio (95% CI)	p value	Frequency in BCG at birth group	Frequency in BCG at 6 weeks group	Hazard ratio	p value	Frequency in BCG at birth group	Frequency in BCG at 6 weeks group	Hazard ratio	p value
**Infectious presentations**												
Total	98	129	0·71 (0·53–0·95)	0·023	88	76	1·10 (0·87–1·40)	0·43	186	205	0·91 (0·76–1·10)	0·33
Male	42	62	0·57 (0·36–0·89)	0·013	41	33	1·11 (0·78–1·59)	0·56	83	95	0·84 (0·63–1·11)	0·22
Female	56	67	0·87 (0·59–1·27)	0·47	47	43	1·11 (0·81–1·52)	0·53	103	110	0·99 (0·78–1·25)	0·93
p_interaction_ between BCG and sex	..	..	..	0·16	..	..	..	0·99	..	..	..	0·37
Birthweight >2500 g	97	115	0·79 (0·59–1·07)	0·12	88	72	1·16 (0·9–1·48)	0·22	185	187	0·99 (0·82–1·19)	0·89
Birthweight ≤2500 g	1	14	0·10 (0·01–0·75)	0·026	0	4	1·31^−8^ (5·64^−9^–3·03^−8^)	<0·0001	1	18	0·07 (0·01–0·45)	0·0061
p_interaction_ between BCG and birthweight	..	..	..	0·044	..	..	..	<0·0001	..	..	..	0·0045
**Serious illness (Integrated Management of Childhood Illness**‡**)**												
Total	33	49	0·68 (0·43–1·07)	0·093	26	18	1·45 (0·82–2·58)	0·20	59	67	0·89 (0·62–1·27)	0·51
Male	10	29	0·34 (0·17–0·69)	0·0028	11	8	1·27 (0·53–3·07)	0·59	21	37	0·54 (0·31–0·94)	0·029
Female	23	20	1·19 (0·65–2·17)	0·57	15	10	1·63 (0·77–3·49)	0·20	38	30	1·33 (0·83–2·15)	0·23
p_interaction_ between BCG and sex	..	..	..	0·0081	..	..	..	0·67	..	..	..	0·014
Birthweight >2500 g	30	43	0·69 (0·43–1·11)	0·13	26	16	1·62 (0·89–2·94)	0·11	56	59	0·94 (0·65–1·38)	0·77
Birthweight ≤2500 g	3	6	0·63 (0·12–3·25)	0·58	0	2	NA	NA	3	8	0·46 (0·09–2·28)	0·34
p_interaction_ between BCG and birthweight	..	..	..	0·90	..	..	..	NA	..	..	..	0·39

NA=numbers too few to analyse.

* Comparison of BCG-vaccinated and BCG unvaccinated infants (ie, comparison of BCG + OPV *vs* OPV).

† Comparison of infants receiving BCG at birth and infants receiving BCG at age 6 weeks (ie, comparison of DTP–Hib–HepB + PCV + OPV [after BCG] *vs* DTP–Hib–HepB + PCV + OPV + BCG); date of EPI-vaccinations in the BCG at birth group was used to establish when they moved into the “Post-BCG in the delayed group” follow-up period (usually day 42).

‡ Serious illness in infants younger than 2 months is defined by the following Integrated Management of Childhood Illness criteria: convulsions or a history of convulsions; not feeding well or vomiting everything; lethargy or unconsciousness or moves only when stimulated or no movement at all or stiff neck; fast breathing (>60 beats per min) or chest in-drawing or stridor in calm child or witnessed apnoeic episodes; axillary temperature of at least 37·5°C or less than 35·5°C; sunken eyes or slow skin pinch; or yellow palms and soles.

During follow-up at age 6–10 weeks (after the delayed group had received BCG), rates of physician-diagnosed, non-tuberculous infections were similar between the two groups (cumulative incidence 33 per 100 in the vaccination at birth group *vs* 29 per 100 in the delayed vaccination group; HR 1·10 [0·87–1·40], p=0·43; figure 2, table 2). For all clinical outcomes, incidence was not significantly different during the 6–10-week follow-up period between infants in the delayed vaccination group and those in the vaccination at birth group (table 2; appendix 3 p 9). The test for interaction between time period and trial group was significant (p=0·023), supporting the suggestion that the effect of trial group on infectious illness presentations is different before and after the delayed group received BCG.

The association of BCG with reduced physician-diagnosed, non-tuberculous infections during the period before BCG vaccination in the delayed group was significant only in boys (HR 0·57 [95% CI 0·36–0·89], p=0·013; table 2), with particularly pronounced reductions in serious illness presentations (HR 0·34 [0·17–0·69], p=0·0028, table 2; appendix 3 p 8). Among girls, there was no significant effect of BCG on infectious presentations (HR 0·87 [0·59–1·27], p=0·47). The test for interaction between BCG and sex was significant for serious illness presentations (p=0·0081), but not overall (p=0·16).

BCG had a protective effect against physician-diagnosed, non-tuberculous infections in infants with a birthweight of 2500 g or less, during the period before BCG vaccination of the delayed group (HR 0·10 [0·01–0·75], p=0·026; test for interaction between BCG and birthweight p=0·044; table 2). By contrast with analysis in all infants, infection rates among low birthweight infants after age 6 weeks remained significantly lower in infants who received BCG at birth than in infants receiving BCG at 6 weeks (table 2). The test for interaction between BCG group and birthweight was significant during this later time period (p<0·0001). Analysis of clinical outcomes in the per-protocol population produced similar results (data not shown).

**Figure 3:**
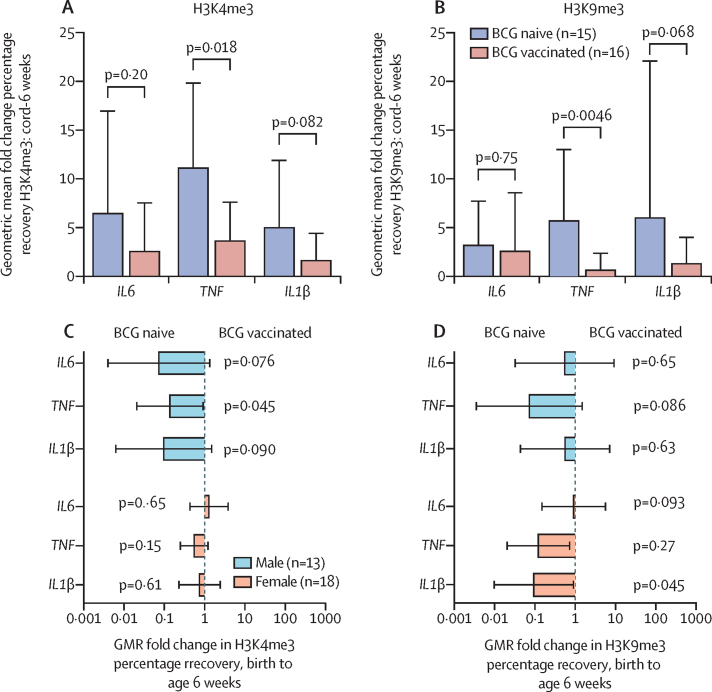
Comparison of fold changes in histone trimethylation at the promoter region of proinflammatory cytokines over the first 6 weeks of life Geometric mean fold change in percentage recovery of H3K4me3 (A) and H3K9me3 (B) at the promoter regions of *TNF, IL6*, and *IL1B* between birth (placental cord blood) and age 6 weeks, comparing infants randomly assigned to BCG at birth (BCG-vaccinated infants) with infants assigned to receive BCG at age 6 weeks (BCG-naive infants). GMRs of the fold change in percentage recovery of H3K4me3 (C) and H3K9me3 (D) comparing BCG-vaccinated infants with BCG-naive infants, by sex. GMR=geometric mean ratio.

Histone trimethylation at the promoter region of proinflammatory cytokines in PBMCs increased significantly over the first 6 weeks of life in all infants (n=31; appendix 3 pp 6, 11). Before the delayed BCG vaccination (ie, age 0–6 weeks), fold increases in both transcriptionally activating (H3K4me3) and repressing (H3K9me3) epigenetic modifications were numerically lower in infants vaccinated at birth than those in the delayed group, although the result was significant only at the *TNF* promoter (figure 3A, B; appendix 3 p 11). The H3K4me3 fold increase at the *TNF* promoter was 3·1 times lower (GMR 0·33 [95% CI 0·13–0·81; p=0·018) and the H3K9me3 fold increase at the *TNF* promoter was 8·9 times lower (GMR 0·11 [0·03–0·48], p=0·0046), in infants in the BCG at birth group than in infants in the delayed BCG group. The numerically smaller fold increase of H3K4me3 in the infants vaccinated at birth versus those in the delayed group was more pronounced in boys than girls, although the difference was only significant at the *IL6* promoter (figure 3). Tests for interaction between sex and BCG group showed p=0·038 at the *IL6* promoter, p=0·12 at the *TNF* promoter, and p=0·12 at the *IL1B* promoter (appendix 3 p 12). No significant effects of BCG on stimulated cytokine production or the inflammatory iron axis were noted at any postnatal timepoint (geometric mean ratios between 0·68 and 1·68; p≥0·038 for all comparisons), with the exception of the expected significant increase in cytokine production in response to the positive control, purified peptide derivative, seen in infants receiving BCG at birth at all timepoints after 5 days, and in infants in the delayed group by age 10 weeks (appendix 3 pp 13–20). At age 6 weeks, 5 days after the delayed group received BCG, there were significant increases in production of some proinflammatory cytokines, in hepcidin, and in unstimulated IL-6 levels in boys who received the BCG vaccine at birth compared with those in the delayed group (appendix 3 p 19), though most results were close to the bounds of conventional statistical significance. Numbers of participants for each of the timepoints in the immunological substudies are in the appendix 3 (pp 5–7).

## Discussion

Trials done in infants with low birthweight in west Africa have shown a reduction in all-cause mortality in the neonatal period associated with BCG at birth.^7^, ^8^, ^9^, ^10^ Our study in healthy Ugandan neonates found a 25% reduction in episodes of physician-diagnosed, non-tuberculous infectious disease in the first 6 weeks of life, in infants who had been randomly allocated BCG vaccine at birth compared with infants who had been assigned to receive BCG vaccine at age 6 weeks and thus had not received it yet. Neonatal BCG was associated with a reduction in the ubiquitous increase in histone trimethylation at the promoter region of proinflammatory cytokines in PBMCs over the first 6 weeks of life, although the reduction was only significant at the *TNF* promoter, indicating that the non-specific effects of BCG in neonates might be mediated in part through epigenetic reprogramming of myeloid cells, albeit in a different manner to that in adults.^5^

This trial is the first to prospectively assess the effect of BCG on all-cause infectious disease morbidity, rather than relying on retrospective analysis of all-cause mortality or hospital admission rates. Our results suggest that BCG produces non-specific beneficial effects against mild and moderate infectious disease presentations, as well as serious illness. The reduction in heterologous infectious disease presentations is strikingly similar to the reduction in all-cause mortality found in larger west African studies,^7^, ^8^, ^9^, ^10^ and that in a systematic review of epidemiological studies.^12^ Similar to the Guinea-Bissau studies, effects were more pronounced in low birthweight babies (ie, birthweight ≤2500 g *vs* >2500 g) and boys (*vs* girls). By contrast with previous RCTs in high-mortality settings^7^ and several epidemiological studies,^19^, ^20^, ^21^ particular reductions in lower respiratory tract infections or sepsis were not seen in our study. This might reflect low event numbers.

This study contributes significantly to the field by providing independent data demonstrating non-specific, BCG-induced protection against infectious disease in neonates from a location geographically distinct from much of the previous work. It also benefits from including healthy, term and preterm, and low birthweight neonates, therefore better reflecting the real-world effect of neonatal BCG vaccination on non-tuberculous infectious disease. Its strict study design and close follow-up of participants enabled reliable identification of illness events, rather than relying on more passive detection methods used in other studies.

A limitation of the study was modest participant numbers, providing inadequate power to detect significant differences in serious illness and death. The follow-up time was also short, meaning that the overall effects of different BCG schedules on morbidity or mortality occurring after age 10 weeks were not investigated. Our results suggest that receipt of BCG at age 6 weeks abrogates the difference in incidence of all-cause infectious disease associated with BCG at birth. This is reassuring, because it suggests that BCG might have a beneficial non-specific effect when given outside of the neonatal period, and therefore short delays to administration might be acceptable. However, because most infant morbidity and mortality occurs in the first month of life, it is likely that most benefit would be derived from earlier administration. Notably, infants with low birthweight receiving BCG at birth continued to have reduced all-cause infectious disease incidence after the delayed group had received BCG at age 6 weeks. Ensuring minimal delays to BCG administration in low birthweight infants might therefore be particularly important, although small numbers prevent firm conclusions being drawn. As the study was not designed specifically to assess the weight-differential and sex-differential effects of BCG, the stronger non-specific effects of BCG in boys and low birthweight infants might be type I errors. However, recruitment was stratified by sex to allow exploration of sex-differential effects, and the similarity to findings in other trials lends weight to the results. Furthermore, the absence of parental masking to BCG status might have confounded the clinical results of the study, if presence or absence of parental anxiety regarding BCG vaccination altered the likelihood of presentation. However, physician and laboratory technician masking was effective. The strengthening of the association between BCG at birth and reduction in illness episodes when data from the masked physicians were analysed independently of parental reports of illness supports a genuine effect of BCG.

The results of this study support a beneficial non-specific effect of BCG, but a number of unanswered questions remain. Notably, a biological mechanism to explain such effects in neonates remains unconfirmed.^11^ Although our study showed reductions in the global increase of histone trimethylation at some cytokine promoters in PBMCs over the first 6 weeks of life, associated with BCG vaccination, we cannot show that this was directly caused by BCG, rather than an indirect effect of the reduced frequency of other illness events. As BCG-associated epigenetic changes in PBMCs occurred for both transcriptionally activating and repressing marks, and corresponding changes to innate cytokine production and the downstream acute-phase inflammatory iron pathway were not shown, it is also impossible to establish whether such changes favour increased or decreased proinflammatory cytokine production in neonates, or have no effect. The more pronounced decrease in H3K9me3 repressor marks in monocytes in our study, known to be associated with a trained immunity phenotype,^22^ might indicate a hyper-responsive immune profile. Studies from Guinea-Bissau^23^ and Australia^24^ report increased proinflammatory cytokine production to heterologous stimuli in neonates after BCG vaccination, although the significant stimuli and cytokines varied. These studies had two to three times more participants than our study, indicating that we might not have had power to show differences in stimulated cytokine production. Alternatively, BCG-associated epigenetic modifications in neonates might alter the speed or quality of cytokine responses to heterologous stimuli, rather than producing quantitative changes. Such differences would not have been identified using our study design. It is intriguing that epigenetic modifications associated with BCG in Ugandan neonates differed in part from those in Dutch adults.^5^ Given the differences between innate and adaptive immunity in adults and neonates, the finding is perhaps not surprising. However, BCG did decrease H3K9me3 in both infants and adults.^22^ Furthermore, the use of PBMCs in our study instead of monocytes as used in the study of Dutch adults (because of sample volume limitations), and the higher exposure of Ugandan neonates to potentially confounding environmental stimuli (eg, infections), means that we cannot rule out that BCG-induced epigenetic reprogramming occurs similarly in neonates as it does in adults, but that we were unable to detect it. It is also possible that the non-specific effects of BCG in neonates occurs via mechanisms not tested in this study. Alternative possibilities include induction of emergency granulopoiesis^25^ and enhanced heterologous T-cell responses.^26^ However, although identification of the biological mechanism underlying the non-specific effects of BCG is important, the exact mechanism responsible for tuberculosis-specific protection from BCG has yet to be established, despite its widespread use for nearly 100 years. Therefore, the current absence of a conclusive biological mechanism should not inhibit public health interventions on the basis of clinical findings of benefit.

The effect of other EPI vaccinations on the non-specific beneficial effects of BCG also remains controversial.^11^ In this study, we cannot exclude the possibility that the equalising of infectious disease presentation rates after age 6 weeks was due to a negative effect of EPI vaccinations in the BCG at birth group, which was abrogated when BCG was given concurrently with EPI vaccinations in the delayed group. It is notable that the only timepoint showing some significant differences in proinflammatory cytokine production and hepcidin was age 6 weeks, 5 days after EPI1 vaccinations in the BCG at birth group and EPI1 vaccinations plus BCG in the delayed group. As similar changes were not seen 5 days after BCG at birth, these findings hint at interactions between BCG and EPI vaccinations. However, because most of these results were close to the bounds of conventional statistical significance, and in the context of multiple comparisons, these findings should be viewed as hypothesis generating for future studies, rather than conclusive.

Concerns regarding the variable specific and non-specific effects of different strains of BCG remain.^16^, ^17^, ^27^ Most studies reporting beneficial non-specific effects of BCG have used BCG-Danish, as we did in this study. However, production issues led to cessation of its supply in 2015. A large trial investigating the effect of early BCG-Russia in Indian neonatal units showed no effect of BCG on all-cause mortality.^15^ A recent study in Guinea-Bissau suggested that BCG-Japan produces greater non-specific effects than either BCG-Russia or BCG-Danish.^27^ Further studies investigating the equivalence of different BCG strains in terms of non-specific protection against heterologous infectious disease would be beneficial.

The mounting evidence that BCG has non-specific beneficial effects, particularly during the neonatal period, has important implications for global public health policy. In areas with high tuberculosis incidence, BCG is recommended at birth, but logistical barriers mean that it is often delayed.^28^ Evidence of non-specific benefits of BCG for neonatal infectious morbidity from this study, in combination with previously published studies showing all-cause mortality reductions, strongly suggest that provision of BCG on the day of birth should be prioritised in areas of high infectious disease burden. Strengthening of existing vaccination programmes to ensure reliable access to BCG and consideration of alternative strategies for early provision, such as training village health workers to vaccinate neonates, might be required to achieve this goal. In areas of low tuberculosis incidence, BCG vaccination is generally limited to specific populations. This study indicates that early BCG, particularly in boys with low birthweight, might be beneficial. Although a Danish trial did not show reduction in hospital admission rates in infancy associated with BCG at birth,^29^ studies investigating the use of BCG as an immunotherapeutic agent in high-risk premature infant populations in neonatal intensive care units might be beneficial. Furthermore, challenges with the variable protection given by BCG against pulmonary tuberculosis means that more effective antituberculosis vaccines are being sought.^30^ If a superior vaccination against tuberculosis is found, our work suggests that the analysis should include all-cause morbidity and mortality outcomes to assess the overall public health impact.

The recent emergence of several novel pathogens, including the pandemic spread of SARS-CoV-2, makes the evidence for BCG-induced non-specific protection particularly important. Developing effective neonatal vaccinations is challenging. Trials assessing the effectiveness of BCG against COVID-19 in health-care workers and older people are ongoing. The addition of trials involving vulnerable neonates in high-income and low-income settings should be strongly considered.

In conclusion, this study provides evidence that BCG at birth significantly reduces all-cause infectious disease morbidity in neonates. Prioritisation of BCG vaccination on the day of birth in settings with high infectious disease morbidity could have major public health benefits.

## Data sharing

The authors recognise the importance of a collaborative approach to research. Relevant anonymised participant data and study documents will be shared with other researchers, upon provision of a methodologically sound, approved proposal. Data will be available beginning after publication of this Article. Proposals should be directed to the corresponding author.

## Supplementary Materials

Luganda translation of the abstract

Swahili translation of the abstract
