# Supplementary material for: BCG-induced non-specific effects on heterologous infectious disease in Ugandan neonates: an investigator-blind randomised controlled trial
Source: Lancet Infect Dis. 2021 Jul;21(7):993–1003. doi: 10.1016/S1473-3099(20)30653-8 (PMC8222005; doi:10.1016/S1473-3099(20)30653-8)
Supplement: Luganda translation of the abstract [file mmc1.pdf]

THE LANCET
Infectious Diseases

Supplementary appendix 1

This translation in Luganda was submitted by the authors and we reproduce it as supplied. It has not been peer reviewed. The Lancet's editorial processes have only been applied to the original in English, which should serve as reference for this manuscript.

Supplement to: Prentice S, Nassanga B, Webb EL, et al. BCG-induced non-specific effects on heterologous infectious disease in Ugandan neonates: an investigator-blind randomised controlled trial. *Lancet Infect Dis* 2021; published online Feb 17. https://doi.org/10.1016/S1473-3099(20)30653-8.

Ekiwandiiko kino ekikyuse mu lulimi Oluganda kyaweereddwayo abawandiisi b'ennyini era tukifulumizza nga bwe baakituwadde. Tekinnakubaganyizibwako birowoozo abakugu abalala. Okusunsula kwa Lancet kukoleddwa ku ekyo eky'Olungereza kyokka nga bwe kyafulumizibwa era kye kirina okujulizibwako ku kiwandiiko kino.

Ebintu eby'awamu ebiva mu ddagala lya Bacillus Calmette-Guérin erigema akafuba ebituuka ku bulwadde obukwata ebintu eby'oluse olw'enjawulo mu baana abate abazaalibwa mu Uganda: okunoonyereza okw'okuteeka abantu mu bibinja eby'enjawulo nga obikkiddwa amaaso.

*Sarah Prentice, Beatrice Nassanga, Emily L Webb, Florence Akello, Fred Kiwudhu, Hellen Akurut, Alison M Elliott, Rob J W Arts, Mihai G Netea, Hazel M Dockrell, Stephen Cose, mu kibinja ky'okunoonyereza ekya BCG**

Obufunze

Ebinnyonnyola Okugezesa okukolebwa mu baana abazaalibwa nga obuzito bwabwe buli wansi nnyo mu bugwanjuba bwa Africa kulaga nti eddagala lya Bacillus Calmette-Guérin (BCG) erigema likendeeza ku byonna ebireeta okufa kw'abaana nga bakyali bawere, nga kirabika kiva ku bukuumi obw'enjawulo ku buwuka obutalwaza. Okunoonyereza kuno kwekenneenya oba nga eddagala lya BCG likyusa byonna ebiviirako obulwadde obusiigibwa nga buyita mu mpewo mu baana abawere abalamu mu bifo eby'enjawulo gye bafa ennyo era ne buzuulwa nga enkyukakyuka zireeteddwawo buleetebwa nga ziyita mu kutendeka omubiri okwerwanako gwokka.

Enkola Kuno kunoonyereza okw'okuteeka abantu mu bibinja eby'enjawulo nga obikkiddwa amaaso okukolebwa ku ddwaaliro ly'e Entebbe mu Uganda. Abaana abawere abaazaalibwa nga embeera yaabwe si nnungi, (kwe kugamba, abaataali bulungi nga tebasobola kusiibulwa butereevu okudda eka nga baakava gye bazaalira kubanga baali beetaaga obujjanjabi), abalina obuzibu ku mubiri oluvannyuma lw'okuzaalibwa, abazadde abalina akawuka akaleeta obulwadde bwa siriimu, okugenda mu maka agamanyiddwa oba agateeberezebwa okubaamu obulwadde bw'akafuba, oba abaana abaali batasabolola kuggyibwako sampolo z'omusaayi ogusigala mu nnabaana, baalekebwa ebbali mu kunoonyereza kuno. Omuwere omulala yenna nga mulamu bulungi era nga asobola okusiibulwa butereevu okuva gye bazaalira yali alina ebisaanyizo by'okwetabamu, awatali kukugirwa ku bbanga lye baakamala ku nsi oba obuzito bwabwe mu kuzaalibwa. Abeetabi baayingizibwanga nga baakazaalibwa era ne bateekebwa mu kibinja awatali kugoberera nsengeka yonna (1:1) okufuna ddoozi ya BCG 1331 (BCG-Danish) ku lunaku lwe bazaalibwa oba nga bawezezza wiiki 6 weeks (nga balondobebwa kyuma kikalimagezi, mu bibinja by'abaana 24, nga basengekebwa okusinziira ku kikula). Abanoonyereza n'abasawo baasibibwa kantuntunu okuweebwa ekibinja; abazadde tebaasibibwa kantuntunu. Abeetabi baalondoolwanga abasawo okutuuka ku bbanga nga bawezezza wiiki 10 ez'obukulu era baawaayo sampolo z'omusaayi eri okumu ku kunoonyereza okw'emirundi esatu okwekenneenya engeri omubiri gye gulwanyisamu obulwadde. Ebikulu ebyavamu bwe bulwadde obwazuulwa abasawo ku buwuka obutalwaza. Ebikulu ebiva mu kwekuuma kw'omubiri byali ebiriisa ebikola obutaffaali obulamba omubiri n'ekkubo eriyitibwamu okubutambuza mu bipimo bya *TNF, IL6*, ne *IL1B;* n'ebyo ebibeera ku mubiri kungulu nga okufulumya TNF, IL-6, IL-1β, IL-10, ne IFN-γ oluvannyuma lw'okukwanaganya obutafaali obutafaanagana; wamu n'obunyisa ensaasaana n'eddaala ly'ekiriisa ekipima obungi bw'ebigumya amagumba ebitambula mu mubiri. Byonna ebivaamu byekenneenyezebwa mu bantu abaakolerwako okugezesebwa kw'ekigendererwa ky'okuwonyezebwa mu bantu bonna abaalondobebwa awatali kugoberera nteekateeka yonna okuggyako abo abaggyayo okukkiriza kwabwe okwetabamu. Okugezesa kuno kwawandiisibwa mu kitongole ekiwandiisa okunoonyereza okw'omutindo gw'eddaala ly'ensi yonna ne kuweebwa n'ennamba (#59683017).

Ebyazuulibwa Wakati w'omwezi Ogwomwenda nga 25, 2014, n'Ogwomusanvu nga 31, 2015, abeetabi 560 be baayingizibwamu era ne bateekebwa mu ebibinja awatali kugoberera nkola yonna ne bafuna BCG nga bazaalibwa (n=280) oba nga bawezezza wiiki 6 (n=280). Abeetabi 12 baateekebwawo okuweebwa BCG nga baakazaalibwa ate abeetabi 11 abaateekebwawo okuweebwa BCG ku bbanga erya wiiki 6 abazadde baabwe baabaggya mu kunoonyereza amangu ddala nga baakateekebwa mu bibinja era tebaateekebwa mu kwekenneenyezebwa. Mu wiiki 6 ezaasooka ez'obulamu nga abawere abaalwawo okuweebwa eddagala erigema tebannaweebwa ddagala lya BCG erigema, abasawo beekennenya ne bazuula nti obulwadde obulala obukwata obutali bwa kafuba bwali butono nnyo mu bawere abaali mu kibinja ekyaweebwa BCG nga baakazaalibwa okusinga abaali mu kibinja ekyalwisibwawo okuweebwa (baali 98 mu kibinja ekyaweebwa BCG nga baakazaalibwa bw'ogeraageranya ne 129 mu kibinja ekyalwisibwa okuweebwa BCG; ekigerero ky'obulwa [HR] 0·71 [95% CI 0·53–0·95], p=0·023). Oluvannyuma lw'okuweebwa BCG mu kibinja ekyalwisibwawo (kwe kugamba, mu bbanga lya wiiki 6–10 nga waliwo okulondoolwa), tewaaliwo njawulo ya maanyi mu abo abalina obulwadde obulala obukwata obutali bwa kafuba mu wakati w'ebibinja (baali 88 bw'ogeraageranya ne 76 abaalabika; HR 1·10 [0·87–1·40], p=0·62). Abaaweebwa BCG nga baakazaalibwa baalaga nti waaliwo okweyongera kwa ebiriisa ebikola obutaffaali obulamba omubiri n'ekkubo eriyitibwamu okubutambuza mu bipimo bya *TNF* mu PBMCs ebibaawo mu wiiki ezisooka 6 ez'obulamu. H3K4me3 kitegeeza okweyongera kuli wansi emirundi 3·1 mu bipimo kya *TNF* (p=0·018), wansi emirundi 2·5 ku kipimo kya *IL6* (p=0·20), n'emirundi egiri wansi 3·1 mu bipimo bya *IL1B* (p=0·082) ne H3K9me3 kitegeeza okweyongera kuli wansi emirundi 8-9 mu bipimo bya *TNF* (p=0·0046), emirundi egiri wansi 1·2 mu bipimo bya *IL6* (p=0·75), n'ebipimo bya mirundi egiri wansi 4·6 mu bipimo bya *IL1B* (p=0·068), mu abo abaagemebwa BCG (BCG mu kibinja ky'abaakazaalibwa) n'abatya BCG (ekibinja ekyalwisibwawo okufuna BCG) mu bawere. Tewali kiva mu BCG ekitangaavu ku ebyo ebibeera ku mubiri kungulu nga okufulumya TNF, IL-6, IL-1β, IL-10, ne IFN-γ oluvannyuma lw'okukwanaganya obutafaali obutafaanagana; wamu n'obunyisa ensaasaana n'eddaala ly'ekiriisa ekipima obungi bw'ebigumya amagumba ebitambula mu mubiri, byakengebwa (ebipimo ebibale byali wakati wa 0·68 ne 1·68; p≥0·038 mu byonna ebyageraageranyizibwa).

Entaputa Okugema kwa BCG kukuuma obutakwatibwa bulwadde obusiigibwa obutali bwa kafuba mu kiseera ng'omwana akyali muwere, kw'ogatta n'okuba n'ebyo ebiva mu bulwadde bw'akafuba. Okuwa BCG enkizo ku lunaku lw'okuzaalibwa lwennyini mu bifo abaana abaakazaalibwa gye bafa ennyo kiyinza okuba n'emiganyulo eri abantu mu by'obulamu nga kiyita mu kukendeeza ku ebyo byonna ebiviirako embeera y'obulwadde wamu n'okufa.

Abateekamu ensimbi Wellcome Trust.

Obwassekkono © 2021 Abawandiisi(s). Kyafulumizibwa aba Elsevier Ltd. Kino kiwandiiko ekisobola okutuukibwako buli ayagadde nga kiri wansi w'olukusa lwa CC BY 4.0.
