# Supplementary material for: BCG-induced non-specific effects on heterologous infectious disease in Ugandan neonates: an investigator-blind randomised controlled trial
Source: Lancet Infect Dis. 2021 Jul;21(7):993–1003. doi: 10.1016/S1473-3099(20)30653-8 (PMC8222005; doi:10.1016/S1473-3099(20)30653-8)
Supplement: Swahili translation of the abstract [file mmc2.pdf]

THE LANCET
Infectious Diseases

Supplementary appendix 2

This translation in Swahili was submitted by the authors and we reproduce it as supplied. It has not been peer reviewed. The Lancet's editorial processes have only been applied to the original in English, which should serve as reference for this manuscript.

Supplement to: Prentice S, Nassanga B, Webb EL, et al. BCG-induced non-specific effects on heterologous infectious disease in Ugandan neonates: an investigator-blind randomised controlled trial. *Lancet Infect Dis* 2021; published online Feb 17. https://doi.org/10.1016/S1473-3099(20)30653-8.

Tafsiri hii katika Swahili iliwasilishwa na waandishi na tunatengeneza tena kama hutolewa. Haijapitiwa. Mchakato wa hariri wa Lancet Global Health umetumika tu kwa asili kwa Kiingereza, ambayo inapaswa kutumika kama kumbukumbu kwa muswada hii.

Bacillus Calmette Guerin hutumia kipengele kinapunguza makali wakati wa matumizi haswa katika magonjwa yaliyo na vitengo vya maambukizi.

*Sarah Prentice, Beatrice Nassanga, Emily L Webb, Florence Akello, Fred Kiwudhu, Hellen Akurut, Alison M Elliott, Rob J W Arts, Mihai G Netea, Hazel M Dockrell, Stephen Cose, for The Delayed BCG Study Team**

Mukhtasari

Historia: Majaribio yaliyofanywa katika watoto wachanga walio na uzito chanya unaonyesh kwamba Chanjo la (BCG) hupunguza vianzo vya magonjwa ya watoto haswa wakati wa siku za kwanza za kuzaliwa. Hii ni kwa sababu labda ya kinga ya maambukizi kwa watoto.

Utafiti huu ulichunguza iwapo Chanjo la (BCG) linazuhia vianzo vya magonjwa ya mda katika afya ya watoto kwenye viwango mbalimbali tangu kuzaliwa.

Mbinu: Mbinu iliyotumika ilikuwa ya kichochezi cha kisengele nyuma cha kijumla na majaribio ya kuongozwa katika hospitali moja Entebbe. Uganda watoto wachanga waliozaliwa na matatizo na hawana uwezo wa kuruhusiwa kwenda nyumbani moja kwa moja baada ya kutoka ukungani huangaliwa na watabibu kwa mda ili kutathimini hali zao za kiafya kabla ya kupokezwa wazazi wao kwenda nyumbani.

Mara nyingi watoto hawa huwa na upungufu wa kinga mwilini au wakati mwingine huwa na maradhi yasio na kinga moja kwa moja ambayo pengine wamezaliwa nao kama walio na virusi vya ukimwi.

Kutoka na utafiti huu waliohusika walipendekezewa kupewa chanjo la kiwango cha wastani la (BCG 1331) ili kuyzuhia makali ya magonjwa hayo katika viwango mbalimbali vya kukuwa kwa watoto hao.

Matokeo yakimsingi yalikuwa ya kihistoria na ambayo yalikuwa yameambata na ukaguzi wa mda wa siku kumi.

Matokeo: Kati ya tarehe 25 mwezi wa Septemba mwaka wa 2014 na tarehe 31 mwezi wa Julai mwaka wa 2015 wahusika 560 walichaguliwa kwa jumla na kupewa chanjo la BCG wakati wa kuzaliwa ama wakati wa umri wa wiki sita ambapo wahusika kumi na mbili waliteuliwa na kupewa chanjo la BCG wakati wa kuzaliwa na wahusika kumi na mmoja waliokuwa wameteuliwa kupewa chajo katika umri wa wiki sita walitolewa tena.

Iligunduliwa kwamba wakati wa umri wa wiki za kwanza kabla mtoto mchanga hajahusishwa katika chanjo lllote ana viwango teule vya kupata magonjwa jambo ambalo linahatarisha maisha yake. Hali hii inamaanisha kwamba chanjo la BCG linastahili kutolewa kwa umri wa kwanza kuzuhia hatari yoyote ya kupata

magonjwa haya ya watoto na maradhi mengine ambayo yanaweza kuhatarisha maisha yao.

Tasfiri: chanjo la BCG huzuhia watoto kutoka kwa magonjwa yanayowaadhiri na pia yaliyo na uwezo wa kuambukizwa kwa urahisi kama vile yale magonjwa ya kifua na kupumua kwa watoto wachanga.

Hii inaacha nafasi ya kutoa chanjo la BCG kwa watoto katika umri wao wa mapema na ni hatua itakayokuwa na umuhimu mkubwa katika maisha ya watoto wachanga na umma kwa jumla.

Wellcome Trust.
Hakimiliki **© 2021** Mwandishi (waandishi)**. Imechapishwa na Elsevier Ltd. Hii ni nakala ya ufikiaji wazi kwa CC BY 4.0 license.**
