# Supplementary material for: BCG-induced non-specific effects on heterologous infectious disease in Ugandan neonates: an investigator-blind randomised controlled trial
Source: Lancet Infect Dis. 2021 Jul;21(7):993–1003. doi: 10.1016/S1473-3099(20)30653-8 (PMC8222005; doi:10.1016/S1473-3099(20)30653-8)
Supplement: Supplementary appendix 3 [file mmc3.pdf]

THE LANCET
Infectious Diseases

Supplementary appendix 3

This appendix formed part of the original submission and has been peer reviewed. We post it as supplied by the authors.

Supplement to: Prentice S, Nassanga B, Webb EL, et al. BCG-induced non-specific effects on heterologous infectious disease in Ugandan neonates: an investigator-blind randomised controlled trial. *Lancet Infect Dis* 2021; published online Feb 17. https://doi.org/10.1016/S1473-3099(20)30653-8.

Detailed Laboratory Methods

Blood sampling

- **Cord Blood:** Cord blood was obtained within 5 minutes of delivery, by direct venepuncture through the outside of the cord into visible umbilical veins using a 10ml syringe and 21-gauge needle. Cord blood was divided equally between heparinised and ethylene diamine tetra-acetic acid (EDTA) blood bottles (Becton-Dickinson, UK**)**. Blood samples were kept at room temperature prior to transfer to the laboratory for processing within a maximum of 16 hours.
- **Infant blood:** 2ml of venous blood was collected from infants from the dorsum of the hands or feet using a 24-gauge cannula, allowing blood to drop into microtainers (Becton-Dickinson, UK) under gravity. This technique was found to be more successful than use of needle and syringe, during pilot work. Samples obtained using either technique were equally sterile upon culturing. Blood for the epigenetic and cytokine sub-studies was collected into sodium-heparinised microtainers. Blood for the iron sub-study was collected into a lithium-heparin microtainer (1.5ml) and an EDTA microtainer (0.5ml). Infant blood samples were stored at room temperature and transferred to the laboratory for processing within a maximum of 4 hours.

Epigenetic sub-study methods

Peripheral blood mononuclear cells (PBMCs) were separated from whole blood using double density centrifugation on ficoll-paque (Sigma-Aldrich, UK) as per the manufacturer's guidelines. PBMCs were fixed in formaldehyde, to ensure stability of epigenetic marks during medium-term storage at 4˚C. Following refrigerated transfer of samples to Radboud University Medical centre Nijmegen PBMC samples were lysed, sonicated and the chromatin stored at -80˚C. Thawed chromatin samples were de-crosslinked by incubating at 65˚C for one hour, and purified using MinElute PCR purification columns (Qiagen, Germany) under negative pressure. Purified chromatin was incubated overnight with antibodies to the epigenetic marks histone-3, lysiene-4 trimethylation (anti-H3K4me3, Diagenode, Belgium) or histone-3, lyseine-9 tri-methylation (anti-H3K9me3, Diagenode, Belgium) and pre-blocked agarose beads (Santa Cruz Protein A/G, USA). Bound chromatin was eluted from beads, de-crosslinked and the DNA purified using MinElute purification columns as previously. Quantitative PCR was then carried out with primers corresponding to *IL6* (forward (5'-3'): AGGGAGAGCCAGAACACAGA, reverse (3'-5'): GAGTTTCCTCTGACTCCATCG), *TNF* (forward: GTGCTTGTTCCTCAGCCTCT, reverse: ATCACTCCAAAGTGCAGCAG) and *IL1β* (forward: AATCCCAGAGCAGCCTGTTG, reverse: AACAGCGAGGGAGAAACTGG). Positive and negative controls used were: myoglobulin (forward: AGCATGGTGCCACTGTGCT, reverse: GCTTAATCTCTGCCTCATGAT) and histone 2B (forward: TGTACTTGGTGACGGCCTTA, reverse: CATTACAACAAGCGCTCGAC) for H3K4me3, zinc fingers (forward: AAGCACTTTGACAACCGTGA, reverse: GGAGGAATTTTGTGGAGCAA) and glycerol-3-phosphate dehydrogenase (forward: CCCCGGTTTCTATAAATTGAGC, reverse: AAGAAGATGCGGCTGACTGT) for H3K9me3. Results were calculated as the percentage of total chromatin that was anti-H3K4me3 or anti-H3K9me3 bound. Where recovery was below the limit of detection, a value of half the lowest level of recovery recorded for that cytokine was assigned. The epigenetic marks studied were chosen to a) provide homology to the only previous similar study in the area and b) to investigate both a stimulatory epigenetic modification (H3K4me3) and an inhibitory epigenetic modification (H3K9me3).

Cytokine sub-study methods

Whole blood, diluted 1:1 with Roswell Park Memorial Institute (RMPI) 1640 medium (Invitrogen, UK, supplemented 4mM L-glutamine, 50,000IU/ml penicillin/streptomycin and 20mM HEPES buffer) was added to pre-prepared stimulant plates to a final dilution of 1:4, and stimulated for 24 hours at 37˚C in air with 5% CO_2. Stimulant plates were pre-prepared in batches and stored at -20˚C until use. The final concentrations of stimulants used were as follows: *Streptococcus pneumonia* ($1x10^6$ colony forming units (CFU)/ml, Radboud University, Nijmegen), *Staphylococcus aureus* ($1x10^6$ CFU/ml, Radboud University, Nijmegen)*, Eschericia coli* ($1x10^6$ CFU/ml, Radboud University, Nijmegen)*, Candida albicans* ($1x10^6$ CFU/ml, Radboud University, Nijmegen), Polyinosinic:polycytidylic acid (Poly I:C 1530/0913, 5 µg/ml Sigma Aldrich, UK), purified peptide derivative (10µg/ml, Statens Serum Institute, Denmark). RPMI 1640 medium was used to provide un-stimulated sample measurements. The four whole pathogens used were heat-killed by exposure to 70˚C temperatures for 2 hours. They were supplied by the Netea group, Radboud University Nijmegen, and were from the same stocks as those used for their study about the training potential of BCG in Dutch adults (Kleinnijenhuis et al, PNAS, 2012). The neonatal pathogens were selected a) to enable direct comparison with the comparable study in Dutch adults b) due to their importance as aetiologies of neonatal infections and c) to provide a representative spectrum of pathogen types. Optimisation of stimulant concentration and duration was conducted in preliminary experiments. FEC (influenza, Epstein Barr virus and cytomegalovirus) was originally planned as the viral-type stimulant. However, optimisation experiments using FEC failed to elicit good cytokine response, so poly I:C was used as a substitute (double-stranded RNA, TLR3 agonist). Harvested supernatants were stored at -80˚C until use.

The production of cytokines in stimulated supernatants was measured using competitive ELISA (BD-OptEIA, Becton Dickinson, UK), conducted in batches. ELISAs were performed according to the manufacturers instructions except that due to sample volume limitations, half the manufacturers recommended sample and reagent volumes were used. This modification has previously been used by our group and was confirmed in optimisation experiments to produce comparable results to the recommended sample

volumes. Assays were conducted in singlecate, due to low supernatant volume. Optimisation assays confirmed low inter-plate variability. Each plate contained two positive control wells, from a single stimulated supernatant batch. Plates with average positive control levels lying outside the normal range, or with a coefficient of variance >15% were re-run (approximately 7% of samples). Cytokines were analysed in the order of sensitivity to freeze-thaw cycles, as determined by optimisation experiments: TNF/IL-10, IL-6/IL-1β, IFN-γ. Any samples with cytokine readings outside the linear portion of the graph were re-run at alternative dilutions. Samples with concentrations below the lower limit of detection were imputed using (limit of detection/√2).

Iron sub-study methods

Lithium-heparinised blood was centrifuged at 400g for 10 minutes at room temperature and the plasma stored in aliquots at -80˚C until use. EDTA samples were transferred immediately to the clinical laboratory for automated full blood count analysis (Coulter $A^{c}T$ 5 Diff CP haematology analyser). Plasma iron indices (iron, TSAT, ferritin, UIBC, TIBC, transferrin and serum transferrin receptor), were measured using an automated analyser (COBAS Integra 400 plus, Roche Diagnostics, USA), calibrated daily prior to use. Hepcidin was measured by competitive ELISA (Bachem-25, California, USA) according to the manufacturer's guidelines, in duplicate. IL-6 (un-stimulated) was measured from stored plasma as described for the cytokine sub-study, in duplicate. Supernatants were subject to one freeze-thaw cycle unless re-runs were required.

Figure A1. Diagram of study procedures

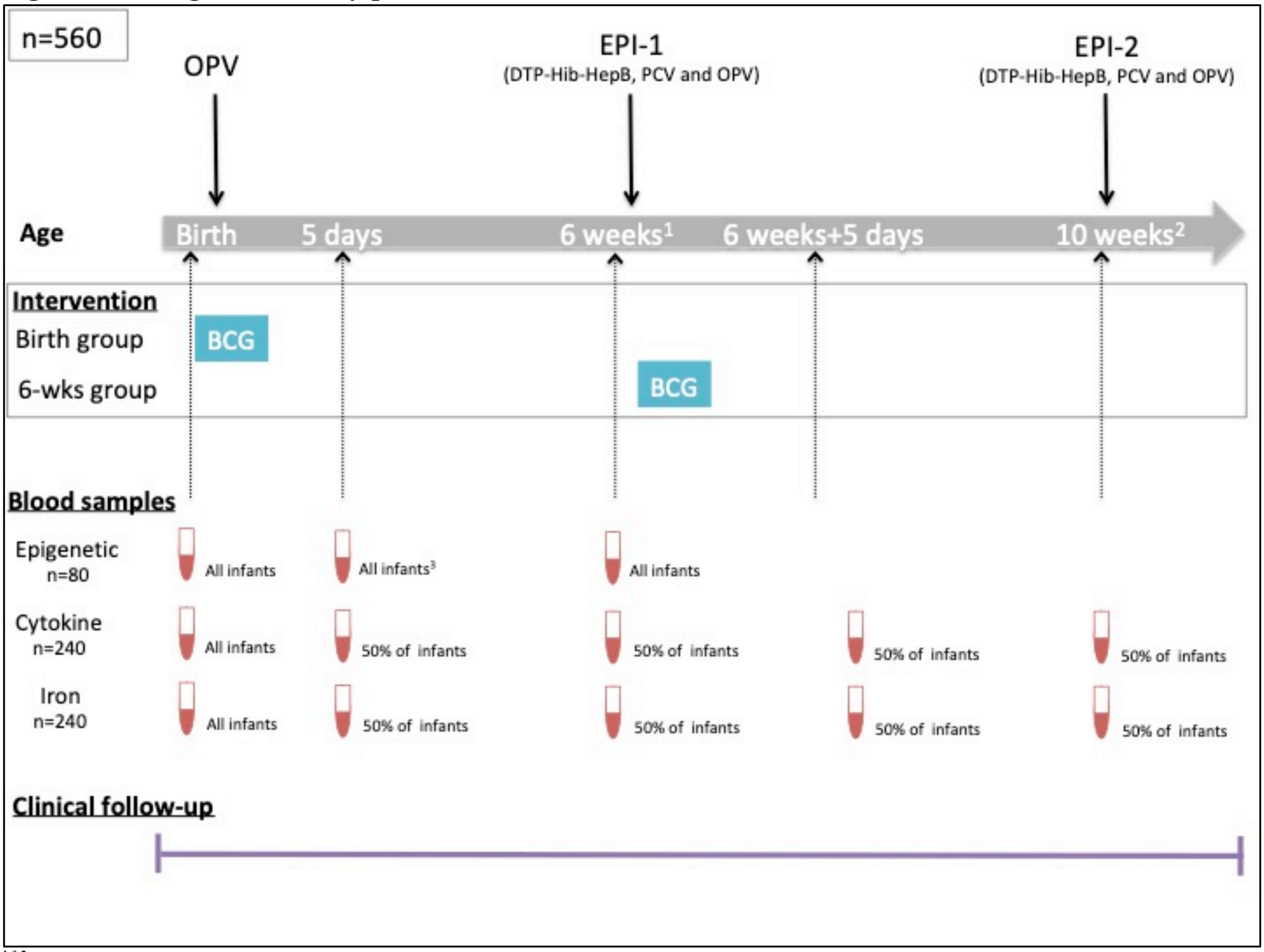

[1&2]In the epigenetic and cytokine sub-studies, blood taken at the 6- and 10-week time-points was taken immediately prior to EPI-vaccinations+/- BCG in the delayed group. In the iron sub-study, EPI vaccinations were used as an in-vivo non-specific stimulant of the iron-inflammatory axis. Therefore blood was taken 1 day after EPI-immunisations (but prior to BCG in the delayed group).
[3]In the epigenetic sub-study, conduct of ChIP experiments was only performed on samples collected at baseline (pre-intervention) and at 6-weeks post-intervention at birth. Funding constraints limited numbers of samples that could be analysed. Samples collected at 6-weeks post-intervention were prioritised over samples collected at 5 days post-intervention, as experiments in adults have not shown effects of trained immunity at such early time-points.
For the cytokine and iron sub-studies each infant had blood taken at only 2 of the possible 4 post-natal sampling points, following guidelines on acceptable blood sampling volumes in infants in research studies. Blood sampling points were randomly allocated. Double the required sample size was recruited for each sub-study to account for this.
OPV: oral polio vaccine, EPI: Expanded programme of immunisations, DTP-Hib-HepB: diphtheria-tetanus-pertusis-haemophilus influenza-hepatitis B, PCV: pneumococcal conjugate vaccine (10-valent), BCG: Bacille-Calmette Guerin.

Table A1. Causes of hospitalisation/death of study participants

	Hospitalisation	Death[1] n= 8
**Possibly infectious**	Lower respiratory tract infection (5)<br>Suspected sepsis (10)	Lower respiratory tract infection (2)<br>Suspected sepsis (1)<br>Respiratory distress in premature neonate (1)<br>Unexplained death/cot-death (2)
**Non-infectious**	Duodenal atresia (1)	Duodenal atresia (1)<br>Cord accident (haemorrhage from avulsed stump) (1)

[1]*One infant was reported to have died two days after withdrawal from the study. This was reported as an unexplained death/cot-death.*

Figure A2. Trial profile for infants in the iron sub-study

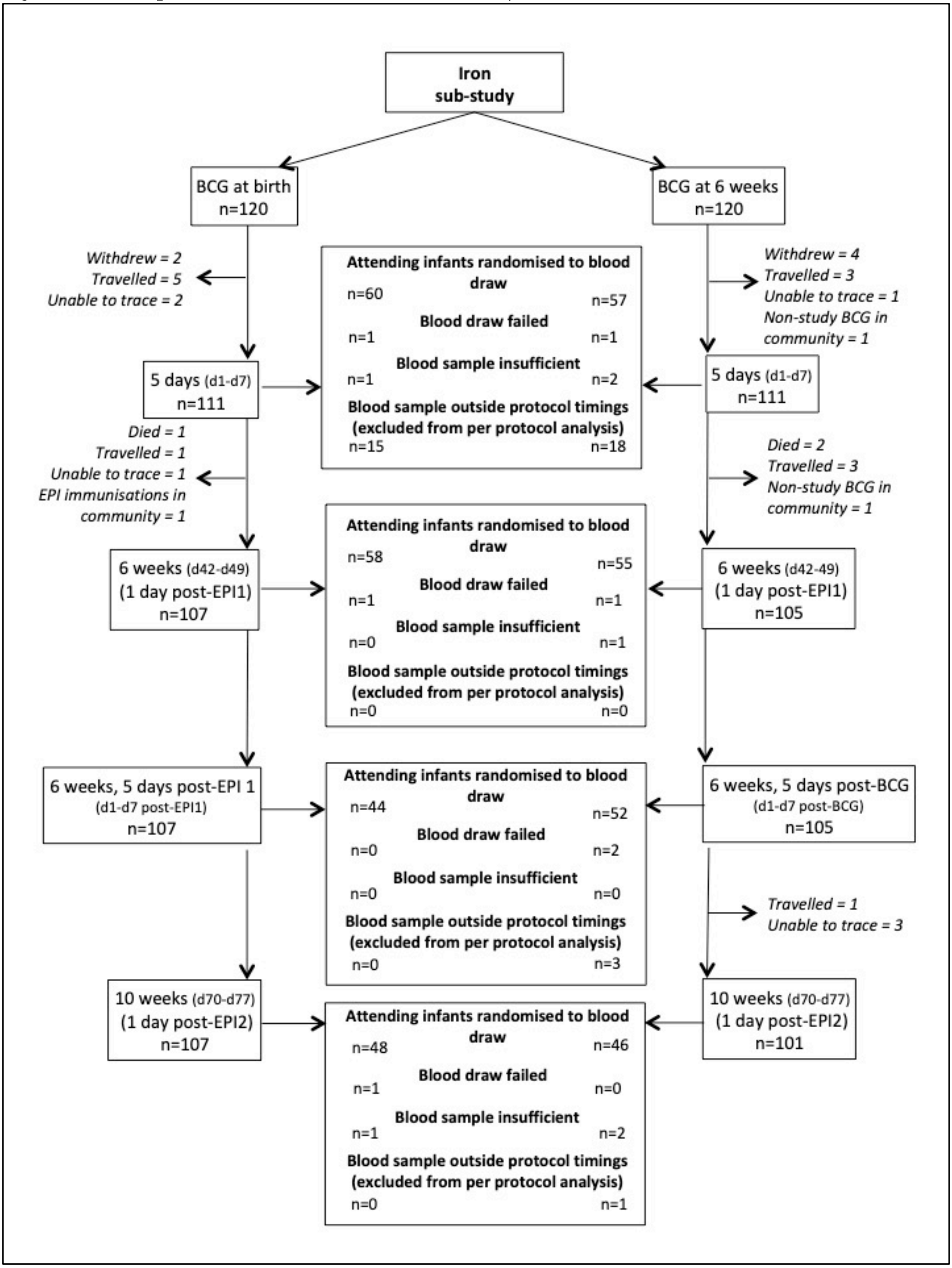

BCG: Bacille-Calmette Guerin, n: number, d: days, EPI: Expanded programme of immunisations (=diptheria, tetanus, whole cell pertussis, haemophillus Influenzae type B, hepatitis B, oral polio vaccine and 10-valent pneumococcal conjugate vaccine given at 6 weeks (EPI 1), 10 weeks (EPI 2) and 14 weeks (EPI 3) of age)

Figure A3. Trial profile for infants in the cytokine sub-study

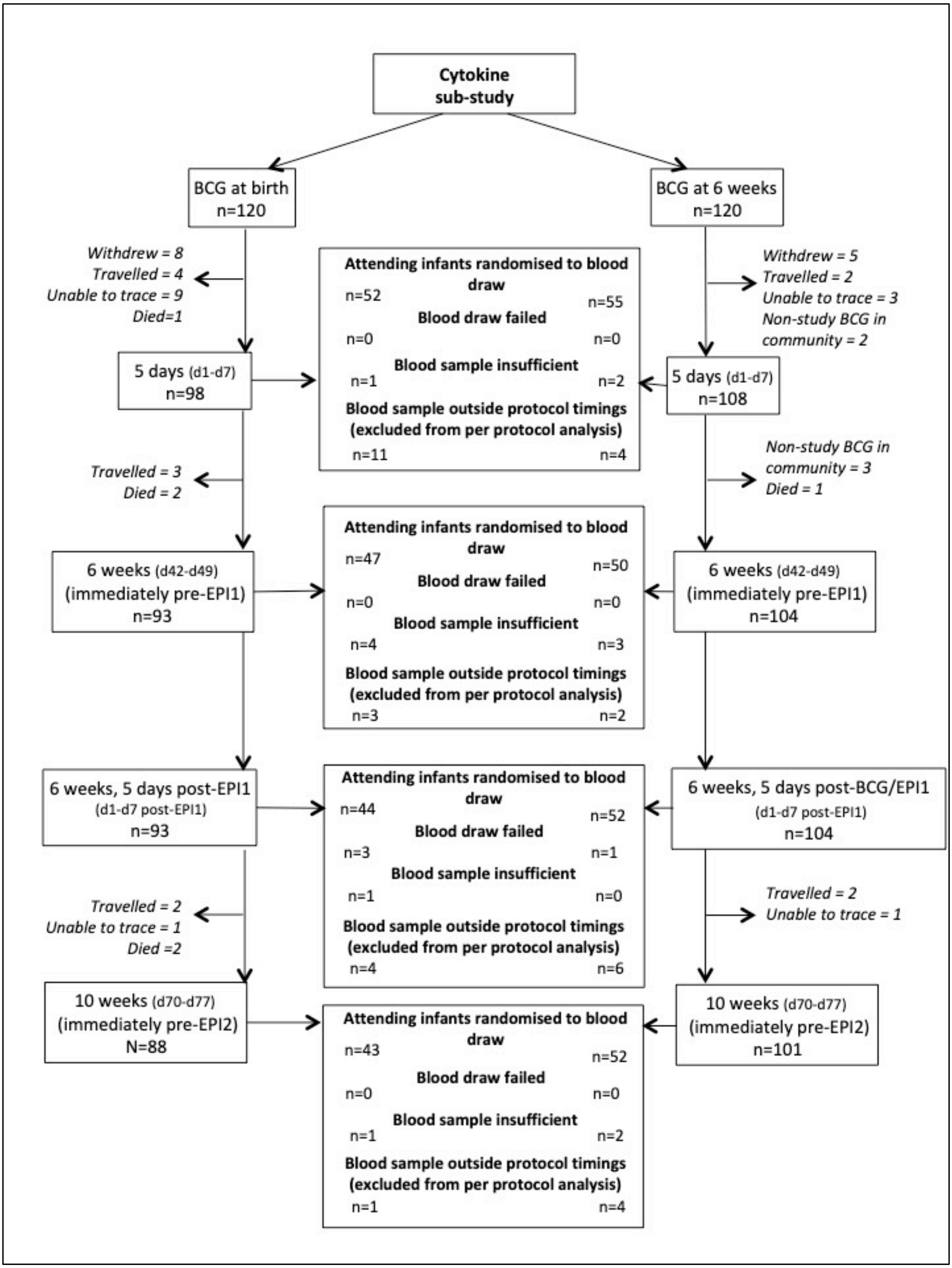

BCG: Bacille-Calmette Guerin, n: number, d: days, EPI: Expanded programme of immunisations (=diptheria, tetanus, whole cell pertussis, haemophillus Influenzae type B, hepatitis B, oral polio vaccine and 10-valent pneumococcal conjugate vaccine given at 6 weeks (EPI 1), 10 weeks (EPI 2) and 14 weeks (EPI 3) of age)

Figure A4. Trial profile for infants in the epigenetic sub-study

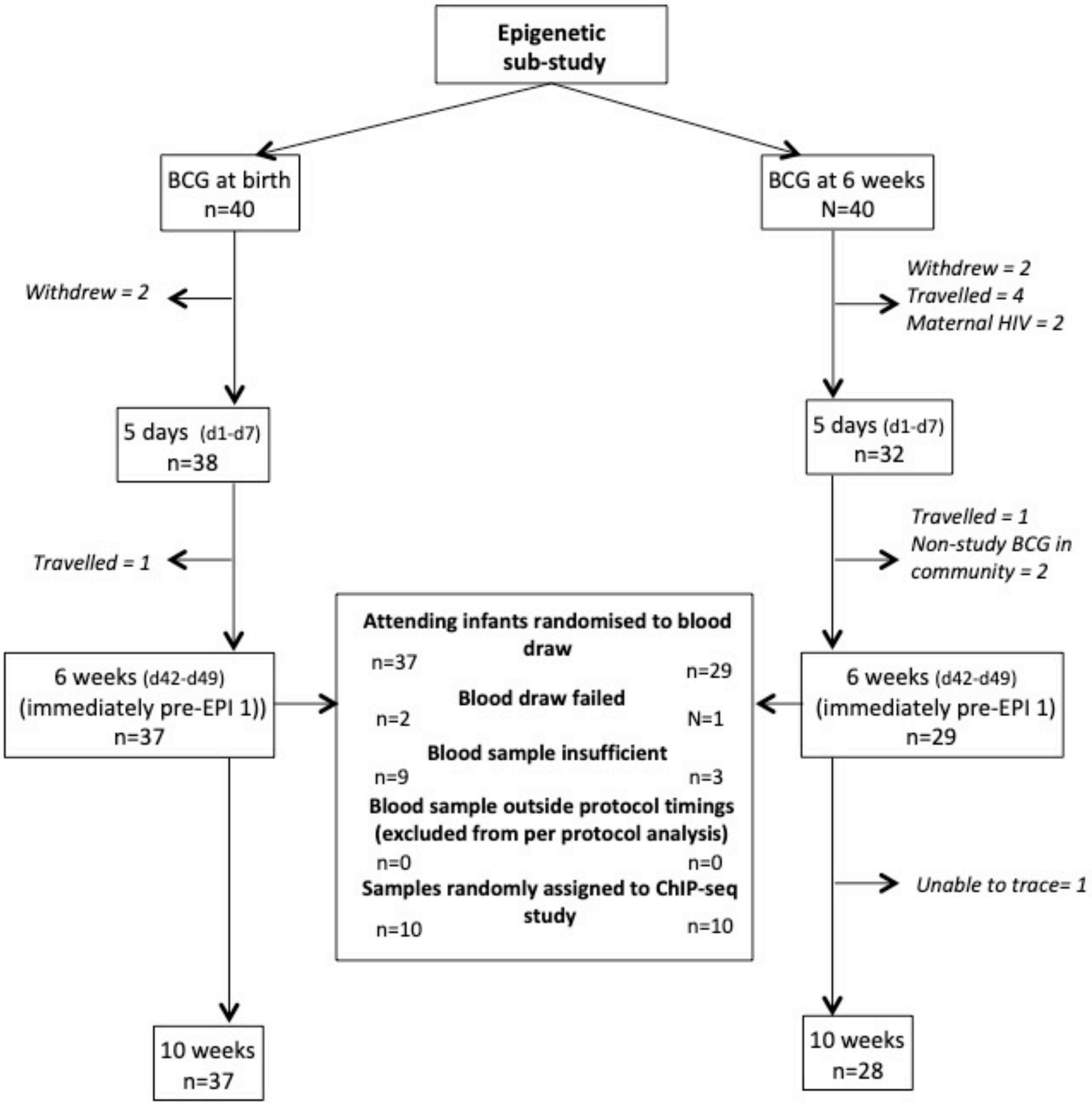

BCG: Bacille-Calmette Guerin, n: number, d: days, EPI: Expanded programme of immunisations (=diptheria, tetanus, whole cell pertussis, haemophillus Influenzae type B, hepatitis B, oral polio vaccine and 10-valent pneumococcal conjugate vaccine given at 6 weeks (EPI 1), 10 weeks (EPI 2) and 14 weeks (EPI 3) of age), ChIP-seq: Chromatin Immunoprecipitation – sequencing.

Figure A5. Cumulative hazard of physician-diagnosed non-tuberculous infections in the first 10-weeks of life by BCG randomisation group and sex

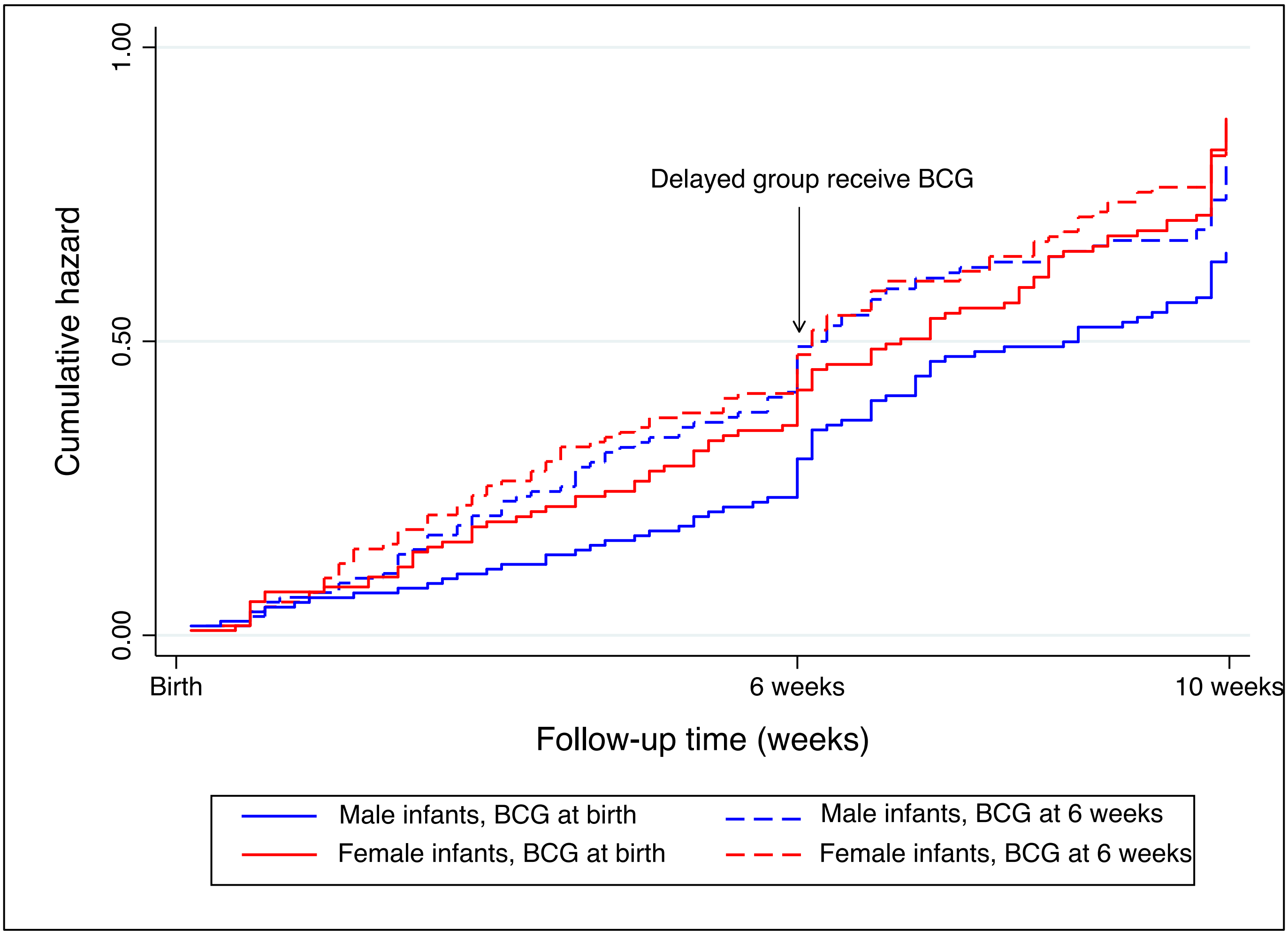

Nelson-Aelen plot of cumulative hazard of physician-diagnosed non-tuberculous infectious disease by BCG randomisation group in the first 10-weeks of life. The observed step at 6-weeks and 10-weeks in both groups is likely to be a function of the study design. Routine clinic appointments were scheduled at 6-weeks and 10-weeks, so parents of infants mild-moderately unwell in the few days preceding a clinic visit may have deferred attendance for convenience, leading to an artificial observed increase in illness rates at each of the clinic days. BCG: Bacille Calmette Guerin.

Table A2. Secondary clinical outcomes comparing infants randomised to receive BCG at birth with infants randomised to receive BCG at 6-weeks of Age

		Pre-BCG in delayed group[1] (0-6 weeks of age)				Post-BCG in delayed group[2] (6-10 weeks of age)				Total follow-up period			
		Frequency BCG at birth	Frequency BCG at 6-weeks	Hazard Ratio[3]	P-value	Frequency BCG at birth	Frequency BCG at 6-weeks	Hazard Ratio	P-value	Frequency BCG at birth	Frequency BCG at 6-weeks	Hazard Ratio	P-value
**Physician - diagnosed illness**	**All presentations**	**128**	**156**	**0·77 (0·60-1·00)**	**0·05**	100	86	1·12 (0·89-1·39)	0·34	228	242	0·94 (0·80-1·11)	0·45
**Culture-positive infections**		2	2			1	0	Numbers too few to analyse		3	2	1·68 (0·26-10·86)	0·59
**Death**		4	3	1·01 (0·20-4·99)	1·0	2	0	Numbers too few to analyse		6	3	1·68 (0·40-7·04)	0·48
**Parental report of illness**	**All reports**	26	28	0.91 (0.52-1.59)	0.73	47	45	1.04 (0.66-1.62)	0.88	73	73	0.98 (0.67-1.43)	0.92
	**Reports of likely infections**	24	27	0.86 (0.49-1.54)	0.62	41	35	1.18 (0.73-1.92)	0.50	65	62	1.04 (0.70-1.55)	0.83

BCG, Bacille Calmette Guerin; [1]Comparison of BCG vaccinated vs. BCG unvaccinated infants, [2]Comparison of infants receiving BCG at birth vs. infants receiving BCG at 6 weeks of age, [3]Comparison of parental reports of previous illnesses by randomisation group were calculated as odds ratios

Table A3. Frequency of infectious presentations by week in study

Age in Weeks (days)	BCG at birth	BCG at 6-weeks
0 (d1-7)	15	15
1 (d8-14)	6	17
2 (d15-21)	16	24
3 (d22-28)	10	20
4 (d29-35)	15	14
5 (d36-42)	24 (15 on d42: routine clinic visit)	27 (17 on d42: routine clinic visit)
6 (d43-49)	23	26
7 (d50-56)	17	10
8 (d57-63)	17	15
9 (d64-70)	29 (19 on d70: routine clinic visit)	16 (11 on d70: routine clinic visit)
10 (d71-77+)	14	21
**Total**	**186**	**205**

BCG, Bacille Calmette Guerin;
Note: In the delayed group, the timing of BCG vaccination was aimed for d42 but was allowed from d36-d48. No participant receiving study BCG in the delayed group received it earlier than d40

Figure A6 and Table A4. Illness aetiologies by BCG group and time post-randomisation

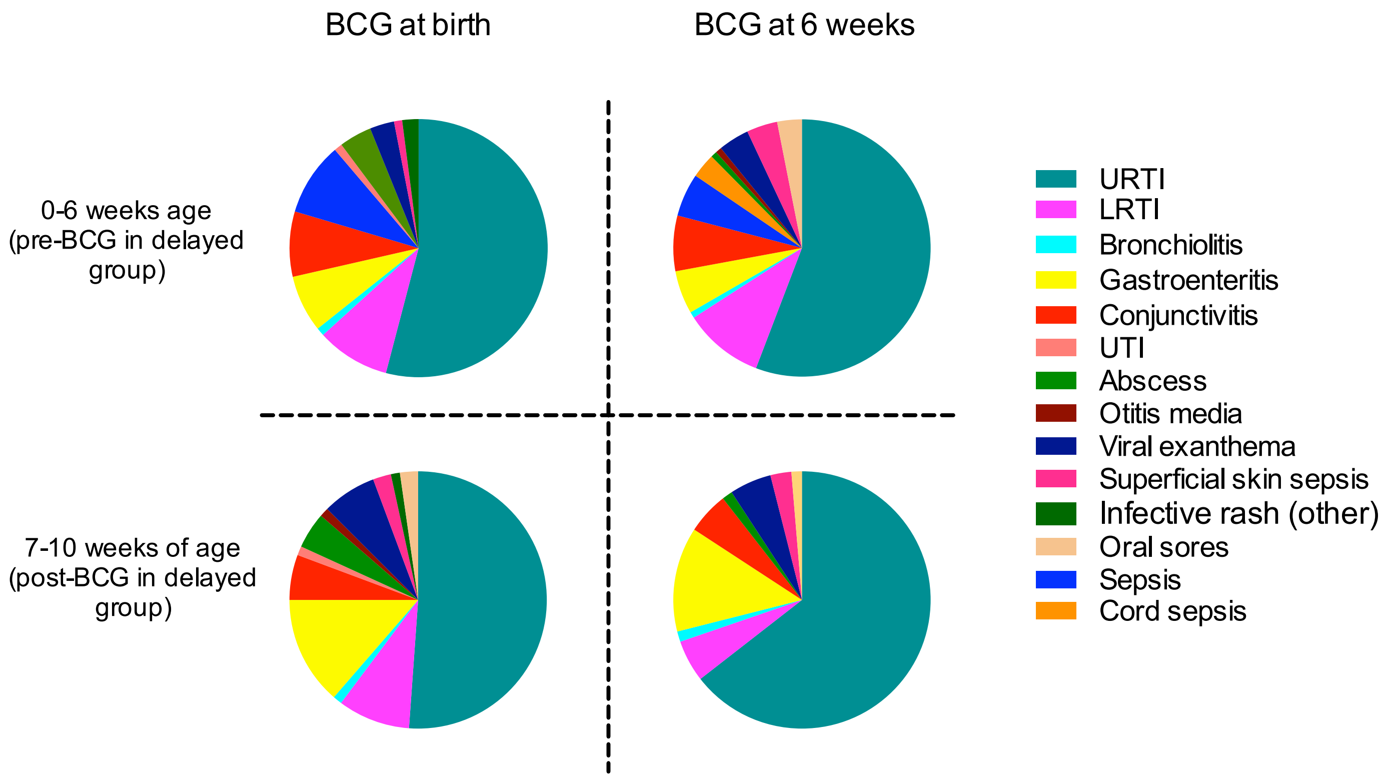

Illness aetiology	0-6 weeks (pre-BCG in delayed group)		6-10 weeks (post-BCG in delayed group)	
	BCG at birth n (%)	BCG at 6 weeks n (%)	BCG at birth n (%)	BCG at 6 weeks n (%)
URTI	53 (54%)	72 (56%)	45 (51%)	49 (64%)
LRTI	9 (9%)	13 (10%)	8 (9%)	4 (5%)
Bronchiolitis	1 (1%)	1 (1%)	1 (1%)	1 (1%)
Gastroenteritis	7 (7%)	7 (5%)	12 (14%)	10 (13%)
Conjunctivitis	8 (8%)	9 (7%)	5 (6%)	4 (5%)
UTI	1 (1%)	0	1 (1%)	0
Abscess	4 (4%)	1 (1%)	4 (5%)	1 (1%)
Otitis media	0	1 (1%)	1 (1%)	0
Viral exanthem	3 (3%)	5 (4%)	6 (7%)	4 (5%)
Superficial skin sepsis	1 (1%)	5 (4%)	2 (2%)	2 (3%)
Infective rash (other)	2 (2%)	0	1 (1%)	0
Oral Sores	0	4 (3%)	2 (2%)	1 (1%)
Sepsis	9 (9%)	7 (5%)	0	0
Cord Sepsis	0	4 (3%)	0	0
**Total**	**98**	**129**	**88**	**76**

BCG: Bacille-Calmette Guerin, URTI: Upper respiratory tract infection, LRTI: Lower respiratory tract infection, UTI: Urinary tract infection.

Figure A7. Median % recovery of histone trimethylation at the promoter region of pro-inflammatory cytokines in PBMCs, from cord blood at blood taken at 6-weeks of age

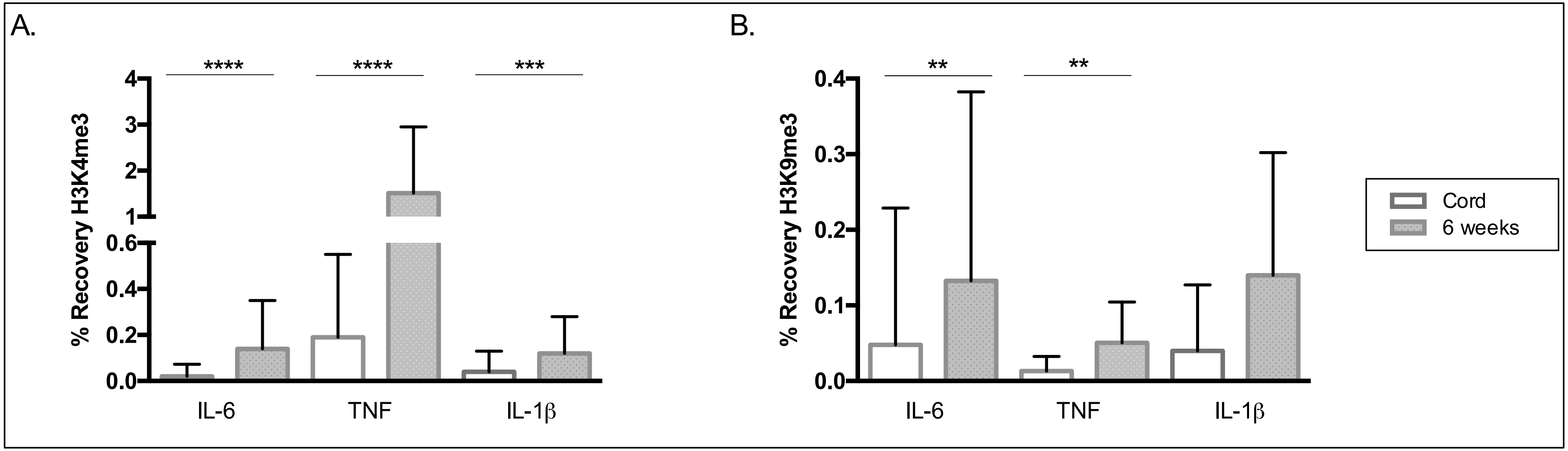

*A. Median % recovery of histone-3, lysine-4 trimethylation (H3K4me3) from the promoter regions of IL-6, TNF and IL-1β in cord blood and blood taken at 6 weeks of age (pre-BCG in delayed group and EPI-vaccinations in both groups). B. . Median % recovery of histone-3, lysine-9 trimethylation (H3K9me3) from the promoter regions of IL-6, TNF and IL-1β in cord blood and blood taken at 6-weeks of age (pre-BCG in delayed group and EPI-vaccinations in both groups). *:p<0.05 **:p<0.01 ***:p<0.001 ***:p<0.0001.*

Table A5. Percentage recovery of epigenetically modified chromatin from the promoter region of pro-inflammatory cytokines, by BCG randomisation group

		Cord blood (baseline)			6 weeks (pre-BCG in delayed group)			Fold-change Birth – 6 weeks			
		Median % recovery		p-value	Median % recovery		p-value	Geometric mean		GMR (95% CI)	p-value
		BCG vaccinated (n=16)	BCG naive (n=15)		BCG vaccinated (n=16)	BCG naive (n=15)		BCG vaccinated (n=16)	BCG naive (n=15)		
H3K4me3	IL-6	0.045	0.02	0.10	0.14	0.17	0.61	2.55	6.44	0.40 (0.10-1.62)	0.20
	TNF	**0.31**	**0.13**	**0.04**	1.29	1.73	0.58	**3.62**	**11.12**	**0.33 (0.13-0.81)**	**0.02**
	IL-1β	0.06	0.02	0.15	0.135	0.12	0.74	1.61	4.99	0.32 (0.09-1.16)	0.08
H3K9me3	IL-6	0.07	0.04	0.55	0.08	0.21	0.41	2.56	3.18	0.81 (0.20-3.29)	0.75
	TNF	0.02	0.005	0.20	**0.05**	**0.09**	**0.03**	**0.64**	**5.69**	**0.11 (0.03-0.48)**	**0.005**
	IL-1β	**0.102**	**0.02**	**0.03**	0.13	0.22	0.60	1.31	5.99	0.22 (0.04-1.13)	0.07

BCG: Bacille-Calmette Guerin, GMR: geometric mean ratio, CI: confidence interval, H3K4me3: Histone-3, lyseine-4 trimethylation, H3K9me3: Histone 3, lyseine-9 trimethylation, IL-6: interleukin-6, TNF: tumour necrosis factor, IL-1β: interleukin-1 beta.

Table A6. Percentage recovery of epigenetically modified chromatin from the promoter region of pro-inflammatory cytokines, by BCG randomisation group and sex

			Cord blood			6 weeks (pre-BCG in delayed group)			Fold-change in % recovery Birth – 6 weeks			
			Median % recovery		p-value	Median % recovery		p-value	Geometric mean		GMR (95% CI)	p-value
			BCG vaccinated	BCG naive		BCG vaccinated	BCG naive		BCG vaccinated	BCG naive		
H3K4me3	IL-6	Male	**0.07**	**0.01**	**0.03**	0.06	0.17	0.43	0.70 (0.05-10.25)	9.57 (1.19-77.06)	0.07 (0.004-1.37)	0.08
		Female	0.02	0.02	1.0	0.15	0.16	0.89	5.54 (2.32-13.24)	4.34 (2.07-9.10)	1.28 (0.41-3.93)	0.65
		Test for interaction between BCG group and sex										**0.04**
	TNF	Male	0.51	0.13	0.07	0.68	1.73	0.25	**1.60 (0.27-9.41)**	**11.56 (2.98-44.91)**	**0.14 (0.02-0.95)**	**0.05**
		Female	0.24	0.135	0.27	1.58	1.74	0.86	5.93 (2.97-11.83)	10.75 (6.61-17.47)	0.55 (0.24-1.26)	0.15
		Test for interaction between BCG group and sex										0.12
	IL-1β	Male	0.12	0.03	0.06	0.14	0.18	0.39	0.72 (0.05-10.04)	7.42 (1.14-48.34)	0.10 (0.006-1.54)	0.09
		Female	0.04	0.02	0.56	0.15	0.11	0.72	2.63 (1.03-6.71)	3.53 (1.47-8.50)	0.74 (0.22-2.50)	0.61
		Test for interaction between BCG group and sex										0.12
H3K9me3	IL-6	Male	0.10	0.04	0.17	0.13	0.11	0.68	1.66 (0.06-47.67)	3.01 (0.49-18.60)	0.55 (0.03-9.64)	0.65
		Female	0.15	0.04	0.96	0.06	0.30	0.10	3.11 (0.64-14.99)	3.37 (1.21-9.35)	0.92 (0.15-5.85)	0.93
		Test for interaction between BCG group and sex										0.73
	TNF	Male	0.02	0.004	0.22	0.05	0.08	0.68	0.34 (0.002-62.30)	4.69 (1.42-15.45)	0.07 (0.003-1.57)	0.09
		Female	0.02	0.01	0.59	**0.03**	**0.15**	**0.02**	**0.83 (0.22-3.17)**	**6.75 (1.60-28.46)**	**0.12 (0.02-0.76)**	**0.03**
		Test for interaction between BCG group and sex										0.74
	IL-1β	Male	**0.12**	**0.03**	**0.05**	0.15	0.03	0.22	1.29 (0.09-17.99)	2.29 (0.40-13.05)	0.56 (0.04-7.48)	0.63
		Female	0.07	0.008	0.17	0.1	0.36	0.08	**1.31 (0.30-5.86)**	**13.90 (1.70-114.01)**	**0.09 (0.009-0.94)**	**0.05**
		Test for interaction between BCG group and sex										0.29

BCG: Bacille-Calmette Guerin, GMR: geometric mean ratio, CI: confidence interval, H3K4me3: Histone-3, lyseine-4 trimethylation, H3K9me3: Histone 3, lyseine-9 trimethylation, IL-6: interleukin-6, TNF: tumour necrosis factor, IL-1β: interleukin-1 beta. Total numbers: Male BCG vaccinated = 6, BCG naïve = 7, Female BCG vaccinated: n=10, BCG naïve = 8

Figure A8. Geometric mean ratios of stimulated cytokine production and inflammatory-iron responses, comparing infants BCG-vaccinated at birth with infants BCG-vaccinated at 6-weeks, at four post-natal time-points

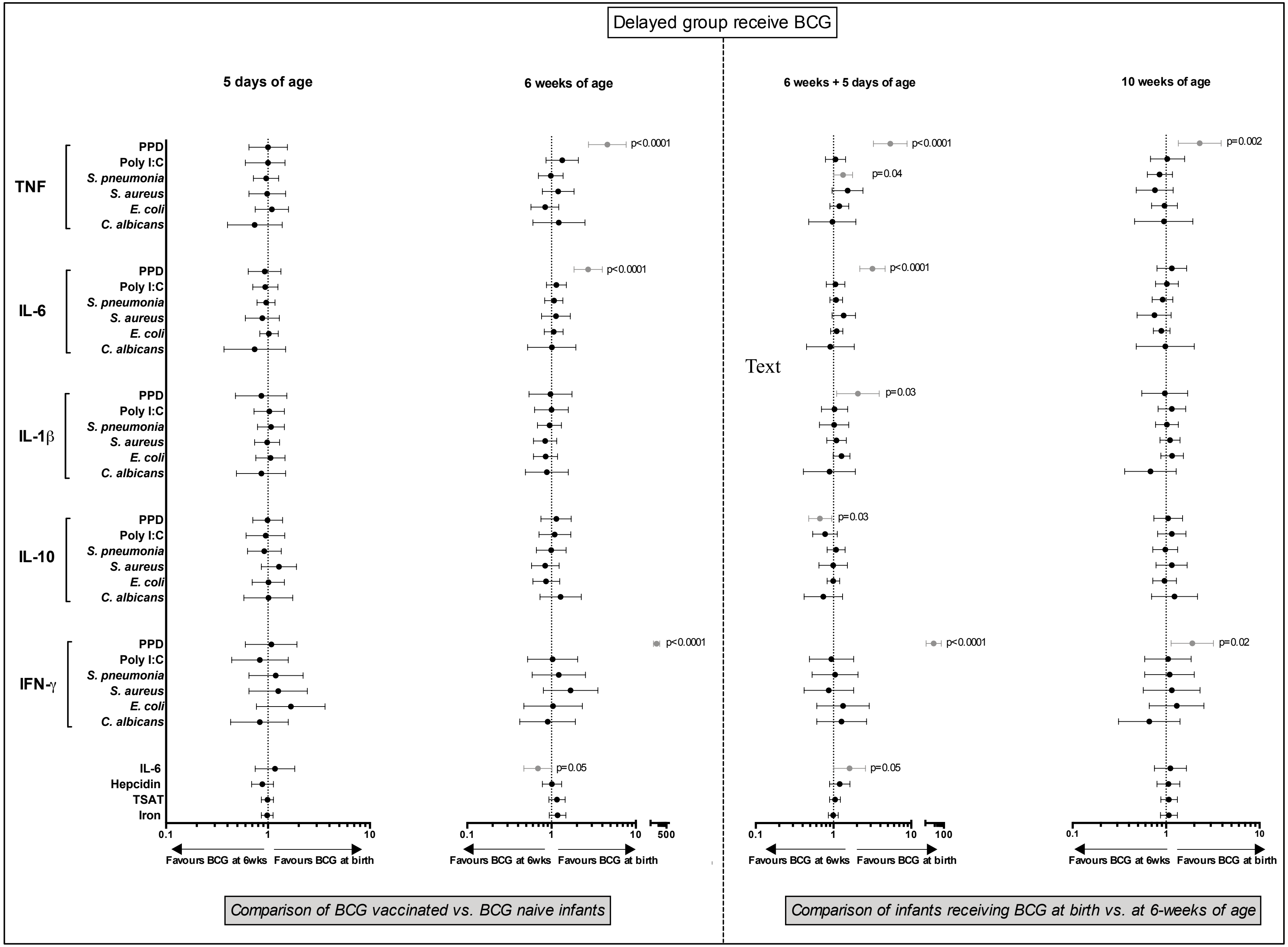

Geometric mean ratios (95% confidence intervals) of a) innate cytokine production measured by ELISA, following 24 hour stimulation of whole blood with bacterial/fungal whole killed pathogens, a viral-type stimulant (Poly I:C) and positive control (PPD) b) circulating inflammatory iron parameters, by BCG status. A geometric mean ratio >1 indicates that levels were higher in the BCG at birth group. The first two postnatal time points compared BCG-vaccinated vs naïve infants. The second two time points compared early (birth) with delayed (6-wks) BCG-vaccinated infants. Comparisons reaching statistical significance are shown in grey. The range of allowable blood-sampling timing at each age were 5d (d1-d7), 6 weeks (d42-49), 6 weeks + 5 days (d1-d7 post EPI-1+/-BCG vaccinations), 10 weeks (d70-77). Adjusting for day of blood sampling during analysis had little effect on the results. BCG: Bacille-Calmette Guerin, GMR: geometric mean ratio, TNF: tumor necrosis factor, IL-6: interleukin-6, IL-1β: interleukin-1beta, IL-10:interleukin 10, IFNγ: interferon-gamma, PPD: purified peptide derivative, Poly I:C: polyinosinic:polycytidylic acid, S. pneumoniae: Streptococcus pneumoniae, S. aureus: Staphylococcus aureus, E.coli: Escherichia coli, C.albicans: Candida albicans, TSAT: transferrin saturation, wks:weeks

Table A7. Geometric mean cytokine levels and geometric mean ratios of cytokine production, comparing infants randomised to receive BCG at birth with infants randomised to receive BCG at 6-weeks of age, at all blood sampling time points. Intention to treat analysis.

Intention to treat analysis		Cord Blood (baseline)				5 days of age				6 weeks of age (pre-EPI1 vaccinations and BCG in delayed group)				6 weeks + 5 days of age (5 days post EPI1 vaccinations + BCG in delayed group)				10 weeks of age (pre EPI2 vaccinations)			
		Geometric means		GMR (95% CI)	p-value	Geometric means		GMR (95% CI)	p-value	Geometric means		GMR (95% CI)	p-value	Geometric means		GMR (95% CI)	p-value	Geometric means		GMR (95% CI)	p-value
		BCG at birth n=112	BCG at 6 weeks n=113			BCG at birth n=51	BCG at 6 weeks n=53			BCG at birth n=43	BCG at 6 weeks n=47			BCG at birth n=40	BCG at 6 weeks n=51			BCG at birth n=42	BCG at 6 weeks n=50		
TNF (pg/ml)	Unstimulated	6.39	6.63	0.96 (0.78-1.20)	0.74	12.42	13.17	0.94 (0.51-1.75)	0.85	10.31	9.20	1.12 (0.67-1.88)	0.66	9.02	8.03	1.12 (0.76-1.67)	0.56	12.76	13.24	0.96 (0.54-1.73)	0.90
	PPD	21.49	20.64	1.04 (0.74-1.46)	0.82	74.44	76.65	1.00 (0.65-1.55)	0.99	**177.03**	**36.73**	**4.59 (2.74-7.68)**	**<0.0001**	**179.60**	**32.01**	**5.38 (3.27-8.84)**	**<0.0001**	**145.47**	**64.27**	**2.29 (1.35-3.88)**	**0.002**
	Poly I:C	229.66	217.69	1.07 (0.77-1.47)	0.69	1279.91	1274.68	1.00 (0.68-1.47)	0.99	1060.56	790.96	1.34 (0.86-2.08)	0.19	999.86	937.29	1.06 (0.79-1.43)	0.7	1105.17	1067.40	1.03 (0.68-1.58)	0.88
	S. pneumoniae	687.98	653.17	1.05 (0.75-1.48)	0.76	1772.98	1860.62	0.96 (0.72-1.27)	0.75	1638.00	1668.06	0.98 (0.70-1.37)	0.92	**1864.19**	**1404.74**	**1.33 (1.01-1.76)**	**0.04**	1493.14	1753.77	0.85 (0.63-1.17)	0.32
	S. aureus	74.23	68.30	1.09 (0.75-1.57)	0.65	246.75	254.90	0.98 (0.65-1.49)	0.92	345.10	288.03	1.20 (0.78-1.85)	0.41	264.18	172.62	1.52 (0.96-2.39)	0.07	226.20	299.98	0.76 (0.48-1.19)	0.23
	E. coli	836.75	692.07	1.22 (0.98-1.53)	0.08	3069.85	2808.02	1.09 (0.75-1.59)	0.65	2172.65	2626.80	0.84 (0.57-1.22)	0.35	2806.61	2356.96	1.19 (0.90-1.57)	0.22	2112.47	2199.06	0.96 (0.70-1.32)	0.79
	C. albicans	77.87	95.91	0.82 (0.49-1.38)	0.46	87.30	122.67	0.74 (0.40-1.38)	0.34	131.37	104.12	1.22 (0.60-2.49)	0.58	109.09	110.32	0.97 (0.48-1.94)	0.93	138.77	148.38	0.95 (0.46-1.93)	0.88
IL-6 (pg/ml)	Unstimulated	15.85	16.52	0.96 (0.54-1.70)	0.89	109.06	112.23	0.97 (0.33-2.83)	0.96	83.65	63.02	1.33 (0.51-3.47)	0.56	96.58	67.97	1.42 (0.61-3.32)	0.41	165.58	101.22	1.64 (0.61-4.36)	0.32
	PPD	4723.70	4364.76	1.05 (0.79-1.38)	0.75	8732.23	8938.70	0.93 (0.64-1.34)	0.68	**9555.42**	**3315.16**	**2.72 (1.85-4.00**	**<0.0001**	**10980.09**	**3678.01**	**3.17 (2.18-4.61)**	**<0.0001**	8902.38	6376.13	1.15 (0.80-1.66)	0.45
	Poly I:C	15784.04	15429.97	1.01 (0.82-1.25)	0.93	39730.22	42387.86	0.94 (0.71-1.25)	0.67	27316.40	23092.36	1.14 (0.87-1.50)	0.32	31057.62	28539.04	1.06 (0.81-1.40)	0.66	31570.28	29763.10	1.02 (0.77-1.35)	0.90
	S. pneumoniae	67806.09	69405.33	0.99 (0.82-1.19)	0.92	83429.82	88370.33	0.96 (0.78-1.17)	0.65	54526.57	50880.19	1.07 (0.83-1.36)	0.60	65826.30	60781.95	1.08 (0.90-1.31)	0.41	51868.66	54826.57	0.92 (0.71-1.18)	0.49
	S. aureus	1773.32	1752.52	1.06 (0.77-1.47)	0.72	4588.19	5112.36	0.88 (0.60-1.29)	0.51	4258.28	3723.16	1.13 (0.76-1.67)	0.55	5088.71	3746.75	1.35 (0.96-1.92)	0.09	4168.86	4521.16	0.75 (0.49-1.13)	0.17
	E. coli	54140.54	51420.97	1.04 (0.87-1.24)	0.67	89928.44	88585.32	1.02 (0.83-1.26)	0.85	58856.48	54567.03	1.06 (0.82-1.37)	0.67	79023.18	73466.20	1.10 (0.92-1.32)	0.30	61301.12	66791.07	0.89 (0.73-1.10)	0.29
	C. albicans	22711.01	23388.27	1.00 (0.65-1.54)	1.00	9870.85	13352.39	0.74 (0.37-1.49)	0.40	9420.65	7564.49	1.01 (0.52-1.94)	0.99	9204.97	8128.97	0.91 (0.45-1.85)	0.79	15165.15	13144.90	0.98 (0.48-2.00)	0.96
IL-1β (pg/ml)	Unstimulated	5.41	6.91	0.78 (0.53-1.15)	0.22	21.99	15.43	1.42 (0.65-3.12)	0.37	16.80	8.26	2.03 (0.99-4.19)	0.06	8.03	10.93	0.73 (0.35-1.52)	0.40	25.07	13.27	1.89 (0.91-3.93)	0.09
	PPD	47.26	47.85	1.04 (0.70-1.56)	0.84	103.27	100.00	0.86 (0.48-1.53)	0.60	84.71	69.15	0.97 (0.54-1.75)	0.92	**94.61**	**51.41**	**2.06 (1.10-3.88)**	**0.03**	109.91	90.49	0.97 (0.55-1.70)	0.90
	Poly I:C	208.59	222.45	0.97 (0.68-1.38)	0.85	1733.38	1609.24	1.03 (0.73-1.45)	0.88	872.12	776.55	1.00 (0.63-1.58)	0.98	958.78	959.55	1.03 (0.70-1.52)	0.87	1597.64	1348.04	1.15 (0.82-1.62)	0.40
	S. pneumoniae	1409.41	1514.06	0.96 (0.72-1.27)	0.76	1576.76	1383.47	1.07 (0.79-1.45)	0.67	1557.48	1531.46	0.95 (0.68-1.31)	0.75	1479.20	1494.85	1.02 (0.66-1.57)	0.93	1619.04	1534.60	1.02 (0.77-1.35)	0.91
	S. aureus	350.60	305.50	1.15 (0.87-1.51)	0.33	718.49	690.78	0.98 (0.74-1.30)	0.89	637.68	704.87	0.84 (0.61-1.15)	0.27	647.64	610.39	1.09 (0.82-1.46)	0.53	765.99	690.13	1.10 (0.86-1.41)	0.45

	**E. coli**	1758.45	1613.57	1.10 (0.86-1.41)	0.45	4236.65	4016.13	1.06 (0.76-1.47)	0.72	3413.48	3978.57	0.85 (0.61-1.18)	0.33	5717.79	4558.38	1.27 (0.99-1.63)	0.06	4939.50	4475.66	1.16 (0.88-1.53)	0.30
	**C. albicans**	145.45	188.56	0.81 (0.45-1.43)	0.46	153.08	151.26	0.86 (0.49-1.49)	0.58	139.80	130.91	0.88 (0.49-1.58)	0.66	71.19	90.05	0.89 (0.41-1.91)	0.76	188.55	231.06	0.68 (0.36-1.28)	0.23
**IL-10 (pg/ml)**	**Unstimulated**	7.05	7.16	0.98 (0.77-1.25)	0.90	11.07	13.28	0.83 (0.69-1.49)	0.95	8.33	8.23	1.01 (0.69-1.49)	0.95	9.78	14.77	1.22 (0.84-1.77)	0.29	12.83	10.05	1.28 (0.80-2.03)	0.30
	**PPD**	86.05	88.44	0.98 (0.76-1.27)	0.88	170.23	186.41	0.99 (0.71-1.39)	0.98	94.03	82.11	1.14 (0.75-1.71)	0.54	**66.54**	**89.69**	**0.67 (0.48-0.95)**	**0.03**	106.31	92.85	1.05 (0.74-1.50)	0.78
	**Poly I:C**	276.24	268.74	1.03 (0.80-1.33)	0.82	514.62	580.19	0.95 (0.61-1.46)	0.81	287.10	260.58	1.09 (0.71-1.69)	0.69	275.81	341.94	0.78 (0.54-1.12)	0.17	418.35	344.85	1.15 (0.81-1.63)	0.44
	**S. pneumoniae**	597.12	638.42	0.93 (0.75-1.16)	0.54	374.57	431.35	0.92 (0.63-1.35)	0.67	276.51	277.87	0.99 (0.66-1.49)	0.96	368.64	329.2907	1.08 (0.83-1.41)	0.54	362.59	359.07	0.98 (0.72-1.33)	0.89
	**S. aureus**	39.20	37.38	1.05 (0.79-1.41)	0.72	67.66	67.62	1.28 (0.86-1.90)	0.23	51.95	61.58	0.84 (058-1.23)	0.37	55.67	54.26	0.99 (0.65-1.51)	0.97	68.75	56.89	1.15 (0.78-1.68)	0.48
	**E. coli**	1066.72	1044.41	1.02 (0.89-1.17)	0.79	1452.53	1470.81	1.01 (0.70-1.45)	0.95	863.65	999.64	0.86 (0.60-1.25)	0.43	1249.94	1221.24	0.99 (0.83-1.20)	0.96	1096.58	1127.00	0.96 (0.72-1.29)	0.78
	**C. albicans**	122.10	151.71	0.81 (0.51-1.26)	0.34	40.83	45.90	1.01 (0.58-1.75)	0.97	44.55	34.56	1.28 (0.73-2.25)	0.39	38.71	49.44	0.74 (0.42-1.31)	0.30	89.68	67.49	1.23 (0.70-2.17)	0.47
**IFN-γ (pg/ml)**	**Unstimulated**	12.53	12.48	1.00 (0.63-1.59)	0.99	13.44	11.29	1.19 (0.67-2.12)	0.55	10.39	10.60	0.98 (0.49-1.95)	0.95	14.52	10.13	1.43 (0.79-2.61)	0.24	11.06	9.41	1.17 (0.69-1.99)	0.55
	**PPD**	13.83	13.13	1.05 (0.66-1.67)	0.83	24.25	20.88	1.08 (0.60-1.92)	0.80	**1253.82**	**18.49**	**68.09 (36.33-127.60)**	**<0.0001**	**1138.62**	**24.70**	**41.39 (21.46-79.82)**	**<0.0001**	**922.86**	**487.73**	**1.91 (1.13-3.21)**	**0.02**
	**Poly I:C**	20.51	17.34	1.18 (0.74-1.89)	0.49	100.00	115.43	0.83 (0.44-1.58)	0.57	172.96	168.40	1.03 (0.52-2.04)	0.93	107.01	102.49	0.94 (0.49-1.82)	0.86	333.15	319.95	1.05 (0.59-1.85)	0.88
	**S. pneumoniae**	11.78	13.39	0.88 (0.60-1.30)	0.53	169.23	141.91	1.19 (0.65-2.21)	0.56	130.48	106.78	1.22 (0.59-2.53)	0.59	135.08	118.19	1.05 (0.53-2.07)	0.89	160.91	149.75	1.09 (0.59-2.00)	0.78
	**S. aureus**	9.43	12.60	0.74 (0.48-1.14)	0.17	89.26	67.93	1.26 (0.65-2.43)	0.49	159.42	94.65	1.68 (0.80-3.54)	0.17	85.77	90.03	0.87 (0.42-1.82)	0.71	175.02	154.44	1.15 (0.57-2.31)	0.69
	**E. coli**	22.74	26.86	0.86 (0.55-1.35)	0.51	225.08	132.48	1.68 (0.77-3.63)	0.19	233.24	223.48	1.04 (0.47-2.32)	0.92	366.82	251.73	1.33 (0.61-2.88)	0.47	345.23	274.82	1.30 (0.66-2.54)	0.44
	**C. albicans**	9.02	9.83	0.92 (0.60-1.39)	0.68	13.82	15.85	0.83 (0.43-1.58)	0.57	38.71	43.00	0.90 (0.42-1.92)	0.79	66.89	47.53	1.27 (0.61-2.66)	0.52	50.71	77.04	0.66 (0.31-1.41)	0.28

Geometric mean ratios were calculated including the log-transformed value of the un-stimulated cytokine levels as a co-variate to account for individual variability. BCG: Bacille-Calmette Guerin, GMR: Geometric Mean Ratios, CI: Confidence interval, TNF: Tumour necrosis factor, IL-6: interleukin-6, IL-1β: interleukin 1-beta, IL-10: interleukin-10, IFN-γ: interferon-gamma, PPD: purified peptide derivative, Poly I:C: Polyinosinic:polycytidylic acid, S. pneumoniae: Streptococcus pneumoniae, S.aureus: Staphylococcus aureus, E.coli: Escherichia coli, C.albicans: Candida albicans.

Table A8. Geometric mean inflammatory-iron parameters and geometric mean ratios, comparing infants randomised to receive BCG at birth with infants randomised to receive BCG at 6-weeks of age, at all blood sampling time points. Intention to treat analysis.

Intention to treat analysis	Cord Blood (baseline)				5 days of age				6 weeks of age (1 day post-EPI1 vaccinations)				6 weeks + 5 days of age (5 days post EPI1 vaccinations + BCG in delayed group)				10 weeks of age (1 day post-EPI2 vaccinations)			
	Geometric means		GMR (95% CI)	p-value	Geometric means		GMR (95% CI)	p-value	Geometric means		GMR (95% CI)	p-value	Geometric means		GMR (95% CI)	p-value	Geometric means		GMR (95% CI)	p-value
	BCG at birth n=117	BCG at 6 weeks n=116			BCG at birth n=58	BCG at 6 weeks n=54			BCG at birth n=57	BCG at 6 weeks n=53			BCG at birth n=44	BCG at 6 weeks n=50			BCG at birth n=46	BCG at 6 weeks n=44		
**IL6 (pg/ml)**	9.62	6.92	1.39 (0.93-2.06)	0.10	8.45	7.21	1.17 (0.75-1.83)	0.48	**43.31**	**62.75**	**0.69 (0.47-1.01)**	**0.05**	**11.30**	**7.00**	**1.61 (1.01-2.58)**	**0.05**	42.57	38.36	1.11 (0.75-1.65)	0.60
**Hepcidin (ng/ml)**	60.93	58.27	1.05 (0.82-1.33)	0.72	92.19	104.45	0.88 (0.69-1.13)	0.32	192.29	189.71	1.01 (0.78-1.32)	0.92	85.58	71.18	1.20 (0.89-1.63)	0.23	201.17	189.55	1.06 (0.80-1.40)	0.67
**TSAT (%)**	41.62	43.96	0.95 (0.86-1.05)	0.29	39.79	40.20	0.99 (0.86-1.13)	0.88	13.27	11.46	1.16 (0.93-1.45)	0.19	26.66	25.46	1.05 (0.89-1.23)	0.57	9.35	8.70	1.07 (0.88-1.32)	0.49
**Iron (µmol/L)**	19.89	20.89	0.95 (0.86-1.05)	0.34	15.48	15.81	0.98 (0.86-1.12)	0.75	6.29	5.33	1.18 (0.94-1.48)	0.15	12.31	12.39	0.99 (0.86-1.15)	0.94	4.89	4.56	1.07 (0.87-1.32)	0.50

BCG: Bacille-Calmette Guerin, GMR: Geometric Mean Ratios, CI: Confidence interval, IL-6: interleukin-6, TSAT: Transferrin saturation.

Table A10. Geometric mean cytokine levels and geometric mean ratios, comparing infants randomised to receive BCG at birth with infants randomised to receive BCG at 6-weeks of age, at all blood sampling time points. Per protocol analysis.

Per protocol[1] analysis		Cord Blood (baseline)				5 days of age				6 weeks of age (pre-EPI1 vaccinations and BCG in delayed group)				6 weeks + 5 days of age (5 days post EPI1 vaccinations +/- BCG in delayed group)				10 weeks of age (pre EPI2 vaccinations)			
		Geometric means		GMR (95% CI)	p-value	Geometric means		GMR (95% CI)	p-value	Geometric means		GMR (95% CI)	p-value	Geometric means		GMR (95% CI)	p-value	Geometric means		GMR (95% CI)	p-value
		BCG at birth n=112	BCG at 6 weeks n=110			BCG at birth n=40	BCG at 6 weeks n=49			BCG at birth n=39	BCG at 6 weeks n=44			BCG at birth n=37	BCG at 6 weeks n=44			BCG at birth n=41	BCG at 6 weeks n=44		
TNF (pg/ml)	Unstimulated	6.39	6.74	0.95 (0.76-1.18)	0.64	12.74	13.26	0.96 (0.48-1.92)	0.91	10.63	8.51	1.25 (0.73-2.13)	0.41	9.16	8.14	1.13 (0.74-1.71)	0.58	11.94	11.42	1.05 (0.59-1.87)	0.88
	PPD	21.49	20.45	1.06 (0.75-1.48)	0.76	74.25	84.38	0.90 (0.56-1.43)	0.65	**188.05**	**32.70**	**5.26 (3.05-9.06)**	**<0.0001**	**178.26**	**33.43**	**5.11 (3.01-8.67)**	**<0.0001**	**141.23**	**54.57**	**2.56 (1.47-4.44)**	**0.001**
	Poly I:C	229.66	214.29	1.09 (0.79-1.50)	0.61	1262.25	1285.68	0.98 (0.62-1.54)	0.92	1042.21	748.72	1.39 (0.87-2.21)	0.17	985.87	929.35	1.05 (0.76-1.45)	0.76	1117.87	1010.54	1.11 (0.70-1.75)	0.65
	S. pneumoniae	687.66	647.29	1.07 (0.75-1.50)	0.72	1779.98	1905.16	0.94 (0.68-1.28)	0.68	1692.30	1615.23	1.04 (0.76-1.44)	0.79	1876.23	1404.44	1.34 (0.98-1.82)	0.06	1477.27	1640.22	0.90 (0.65-1.24)	0.51
	S. aureus	74.23	66.62	1.12 (0.77-1.63)	0.54	248.92	262.04	0.96 (0.60-1.52)	0.86	344.41	277.19	1.24 (0.78-1.98)	0.36	257.17	174.52	1.46 (0.89-2.38)	0.13	221.73	282.71	0.78 (0.48-1.26)	0.31
	E. coli	836.75	685.84	1.24 (0.99-1.55)	0.07	2999.29	2890.27	1.04 (0.68-1.58)	0.87	2233.75	2533.18	0.90 (0.61-1.31)	0.57	2858.51	2465.76	1.16 (0.85-1.58)	0.35	2147.10	2215.37	0.97 (0.69-1.37)	0.86
	C. albicans	77.87	98.28	0.80 (0.47-1.36)	0.42	86.63	123.17	0.72 (0.37-1.43)	0.35	125.48	97.08	1.20 (0.57-2.55)	0.62	126.41	99.48	1.24 (0.60-2.59)	0.55	133.34	118.34	1.11 (0.53-2.35)	0.78
IL-6 (pg/ml)	Unstimulated	15.85	16.47	0.96 (0.54-1.72)	0.90	115.58	103.21	1.12 (0.34-3.72)	0.85	84.53	62.03	1.36 (0.52-3.60)	0.53	102.77	68.52	1.50 (0.59-3.82)	0.39	165.58	92.00	1.80 (0.67-4.85)	0.24
	PPD	4723.70	4430.94	1.03 (0.78-1.36)	0.85	8576.65	9138.93	0.85 (0.57-1.27)	0.43	**9657.99**	**3200.19**	**2.75 (1.86-4.07)**	**<0.0001**	**10955.64**	**3733.29**	**3.07 (2.04-4.61)**	**<0.0001**	8655.37	5650.41	1.30 (0.92-1.85)	0.14
	Poly I:C	15784.04	15441.44	1.01 (0.81-1.25)	0.94	40138.74	42351.43	0.95 (0.69-1.31)	0.75	27701.45	22553.83	1.18 (0.90-1.54)	0.24	30594.84	28961.34	1.01 (0.76-1.35)	0.94	31268.03	27412.04	1.10 (0.83-1.46)	0.49
	S. pneumoniae	67806.09	68474.3	1.00 (0.83-1.21)	0.97	82160.53	89264.36	0.94 (0.75-1.17)	0.56	53193.18	48945.98	1.06 (0.82-1.37)	0.64	66179.38	59733.77	1.10 (0.90-1.33)	0.36	51481.11	52415.10	0.96 (0.74-1.24)	0.73
	S. aureus	1773.32	1754.88	1.06 (0.76-1.47)	0.73	4297.55	5218.51	0.76 (0.50-1.14)	0.18	4307.91	3475.21	1.16 (0.77-1.75)	0.48	5094.94	3554.96	1.39 (0.97-1.99)	0.07	4021.59	3898.80	0.86 (0.57-1.30)	0.46
	E. coli	54140.54	50838.94	1.05 (0.88-1.25)	0.58	87800.33	88120.67	1.00 (0.79-1.27)	0.98	60161.49	53691.23	1.09 (0.84-1.43)	0.50	80408.62	74354.33	1.10 (0.90-1.34)	0.35	61601.48	63378.00	0.93 (0.76-1.15)	0.52
	C. albicans	22711.01	23353.27	1.00 (0.65-1.55)	0.99	8134.38	12783.76	0.60 (0.27-1.31)	0.19	8830.85	7002.31	0.99 (0.50-1.96)	0.98	9022.05	7148.40	0.96 (0.45-2.05)	0.91	14675.15	11144.96	1.14 (0.55-2.39)	0.72
IL-1β (pg/ml)	Unstimulated	5.41	7.06	0.77 (0.52-1.13)	0.18	24.60	16.05	1.53 (0.65-3.60)	0.32	17.85	8.25	2.16 (1.02-4.61)	0.05	8.33	8.76	0.95 (0.44-2.05)	0.90	23.63	11.98	1.97 (0.93-4.18)	0.08
	PPD	47.26	48.61	1.03 (0.69-1.55)	0.88	103.19	107.31	0.76 (0.41-1.42)	0.39	84.26	65.87	0.94 (0.50-1.78)	0.86	**96.74**	**46.28**	**2.13 (1.12-4.05)**	**0.02**	102.41	75.70	1.11 (0.62-1.98)	0.72
	Poly I:C	208.59	223.68	0.96 (0.67-1.38)	0.84	1840.18	1590.81	1.08 (0.74-1.57)	0.69	939.41	744.96	1.08 (0.69-1.69)	0.72	987.17	902.79	1.10 (0.72-1.68)	0.67	1564.18	1269.72	1.23 (0.86-1.74)	0.25
	S. pneumoniae	1409.41	1511.00	0.96 (0.72-1.28)	0.78	1625.53	1368.33	1.09 (0.78-1.53)	0.61	1505.50	1476.32	0.92 (0.65-1.30)	0.63	1454.86	1446.96	1.01 (0.63-1.62)	0.96	1582.58	1482.35	1.05 (0.79-1.39)	0.74
	S. aureus	350.60	302.06	1.16	0.30	705.67	697.99	0.93	0.66	663.68	732.01	0.84 (0.60-	0.29	641.34	584.06	1.10	0.53	759.68	679.62	1.13 (0.89-1.45)	0.32

				(0.88-1.54)				(0.69-1.27)				1.17)				(0.81-1.49)					
	E. coli	1758.45	1609.27	1.11 (0.86-1.42)	0.43	4546.82	3861.38	1.18 (0.83-1.69)	0.35	3481.41	3192.66	0.88 (0.62-1.24)	0.45	5606.34	4465.21	1.26 (0.97-1.64)	0.09	4948.97	4197.91	1.25 (0.93-1.67)	0.14
	C. albicans	145.45	188.81	0.81 (0.45-1.45)	0.47	154.59	150.31	0.83 (0.47-1.48)	0.52	134.33	127.43	0.80 (0.43-1.50)	0.48	87.33	82.98	1.07 (0.50-2.31)	0.85	178.04	205.05	0.75 (0.38-1.45)	0.38
**IL-10 (pg/ml)**	**Unstimulated**	7.05	7.16	0.98 (0.77-1.26)	0.90	10.33	13.77	0.75 (0.40-1.41)	0.37	8.24	8.02	1.03 (0.69-1.53)	0.89	9.93	7.96	1.25 (0.83-1.88)	0.29	12.03	10.29	1.17 (0.73-1.88)	0.52
	**PPD**	86.05	89.55	0.97 (0.75-1.26)	0.81	165.53	189.68	1.00 (0.67-1.48)	0.99	93.29	80.02	1.14 (0.74-1.78)	0.55	**62.08**	**85.89**	**0.65 (0.45-0.93)**	**0.02**	101.80	85.55	1.13 (0.78-1.62)	0.52
	**Poly I:C**	276.24	266.43	1.04 (0.80-1.35)	0.78	490.86	576.94	0.95 (0.57-1.57)	0.84	288.45	252.18	1.12 (0.70-1.79)	0.62	281.23	333.03	0.81 (0.55-1.21)	0.31	404.97	313.74	1.25 (0.86-1.80)	0.24
	S. pneumoniae	597.12	631.86	0.94 (0.76-1.17)	0.60	331.76	428.95	0.85 (0.54-1.32)	0.46	269.64	261.10	1.02 (0.66-1.56)	0.93	371.13	317.67	1.12 (0.85-1.48)	0.40	354.32	343.64	1.01 (0.74-1.39)	0.95
	S. aureus	39.20	36.23	1.09 (0.81-1.45)	0.57	57.25	70.26	1.15 (0.73-1.81)	0.54	55.14	58.28	0.94 (0.64-1.38)	0.74	60.12	52.22	1.11 (0.72-1.71)	0.62	66.23	50.18	1.30 (0.88-1.91)	0.18
	E. coli	1066.72	1041.51	1.02 (0.66-1.55)	0.94	1416.13	1450.91	0.90 (0.61-1.34)	0.61	888.12	979.06	1.06 (0.87-1.27)	0.57	1290.68	1183.68	1.06 (0.87-1.27)	0.57	1086.38	1092.91	0.98 (0.72-1.35)	0.92
	C. albicans	122.10	151.85	0.80 (0.51-1.26)	0.34	33.75	44.52	0.92 (0.50-1.69)	0.79	40.90	33.33	1.20 (0.67-2.16)	0.53	41.77	44.16	0.89 (0.48-1.65)	0.71	85.25	56.05	1.45 (0.81-2.58)	0.21
**IFN-γ (pg/ml)**	**Unstimulated**	12.53	12.80	0.98 (0.61-1.56)	0.93	15.36	11.11	1.38 (0.72-2.65)	0.32	10.59	9.94	1.07 (0.52-2.20)	0.86	13.60	10.92	1.24 (0.67-2.31)	0.49	10.69	9.17	1.17 (0.67-2.03)	0.59
	**PPD**	13.83	12.99	1.07 (0.68-1.70)	0.76	17.49	20.21	0.74 (0.40-1.34)	0.31	**1287.61**	**18.01**	**70.44 (37.29-133.07)**	**<0.0001**	**1111.50**	**24.33**	**42.13 (21.17-83.83)**	**<0.0001**	**923.22**	**445.81**	**2.08 (1.22-3.56)**	**0.008**
	**Poly I:C**	20.51	17.44	1.18 (0.74-1.90)	0.49	106.00	110.19	0.90 (0.44-1.83)	0.77	170.57	166.71	1.01 (0.50-2.03)	0.98	102.84	100.95	0.96 (0.48-1.95)	0.92	333.53	317.29	1.05 (0.57-1.93)	0.87
	S. pneumoniae	11.78	13.44	0.88 (0.60-1.29)	0.51	167.19	137.41	1.25 (0.63-2.47)	0.52	119.34	108.16	1.11 (0.53-2.33)	0.79	134.44	123.89	1.03 (0.50-2.11)	0.94	160.21	156.24	1.04 (0.54-1.99)	0.92
	S. aureus	9.43	12.88	0.74 (0.48-1.13)	0.16	90.03	66.40	1.26 (0.60-2.67)	0.54	148.00	102.09	1.45 (0.66-3.17)	0.35	80.31	90.17	0.84 (0.39-1.82)	0.65	183.34	161.75	1.14 (0.55-2.36)	0.72
	E. coli	22.74	27.07	0.84 (0.53-1.33)	0.46	211.26	123.87	1.68 (0.71-3.95)	0.23	232.20	220.02	1.05 (0.46-2.4)	0.90	354.22	258.88	1.29 (0.56-3.00)	0.54	364.13	272.34	1.37 (0.68-2.74)	0.38
	C. albicans	9.02	10.00	0.91 (0.59-1.38)	0.65	11.33	15.75	0.66 (0.33-1.32)	0.24	38.40	45.63	0.833 (0.39-1.78)	0.64	72.13	44.40	1.53 (0.72-3.24)	0.26	54.57	63.53	0.85 (0.40-1.80)	0.67

[1]Per protocol analysis excludes any infant not receiving study BCG (SSI-1331) as per their randomisation group (0 in BCG at birth group and 5 in BCG at 6 weeks group (received non-study BCG in the community prior to 6 week follow-up visit)) and any blood sample taken outside the protocol timings (see Figure A2). Geometric mean ratios were calculated including the log-transformed value of the unstimulated cytokine levels as a co-variate to account for individual variability. BCG: Bacille-Calmette Guerin, GMR: Geometric Mean Ratios, CI: Confidence interval, TNF: Tumour necrosis factor, IL-6: interleukin-6, IL-1β: interleukin 1-beta, IL-10: interleukin-10, IFN-γ: interferon-gamma, PPD: purified peptide derivative, Poly I:C: Polyinosinic:polycytidylic acid, S. pneumoniae: Streptococcus pneumoniae, S.aureus: Staphylococcus aureus, E.coli: Escherichia coli, C.albicans: Candida albicans.

Table A11. Geometric mean inflammatory-iron parameters and geometric mean ratios, comparing infants randomised to receive BCG at birth with infants randomised to receive BCG at 6-weeks of age, at all blood sampling time points. Per protocol analysis.

Per protocol[1] analysis	Cord Blood (baseline)				5 days of age				6 weeks of age (1 day post-EPI1 vaccinations)				6 weeks + 5 days of age (5 days post EPI1 vaccinations + BCG in delayed group)				10 weeks of age (1 day post-EPI2 vaccinations)			
	Geometric means				Geometric means				Geometric means				Geometric means				Geometric means			
	BCG at birth n=110	BCG at 6 weeks n=113	GMR (95% CI)	p-value	BCG at birth n=41	BCG at 6 weeks n=35	GMR (95% CI)	p-value	BCG at birth n=53	BCG at 6 weeks n=52	GMR (95% CI)	p-value	BCG at birth n=42	BCG at 6 weeks n=47	GMR (95% CI)	p-value	BCG at birth n=46	BCG at 6 weeks n=42	GMR (95% CI)	p-value
**IL-6 (pg/ml)**	**10.12**	**6.72**	**1.51 (1.00-2.26**	**0.05**	9.75	7.45	1.31 (0.75-2.28)	0.34	**44.85**	**65.11**	**0.69 (0.47-1.00)**	**0.05**	**11.41**	**7.00**	**1.63 (1.00-2.66)**	**0.05**	42.57	40.09	1.06 (0.72-1.58)	0.76
**Hepcidin (ng/ml)**	60.37	57.80	1.04 (0.81-1.34)	0.73	109.15	115.63	0.94 (0.73-1.22)	0.66	192.50	192.68	1.00 (0.76-1.32)	0.99	87.85	73.37	1.20 (0.88-1.62)	0.24	201.17	198.60	1.01 (0.77-1.33)	0.93
**TSAT (%)**	41.09	44.28	0.93 (0.84-1.03)	0.16	43.30	42.92	1.01 (0.87-1.17)	0.90	13.28	11.20	1.19 (0.95-1.48)	0.13	27.31	25.04	1.09 (0.93-1.29)	0.30	9.35	8.54	1.09 (0.89-1.35)	0.39
**Iron (µmol/L)**	19.67	21.01	0.94 (0.84-1.04)	0.21	16.18	16.34	0.99 (0.83-1.17)	0.91	6.41	5.20	1.23 (0.98-1.55)	0.07	12.72	12.16	1.05 (0.90-1.21)	0.54	4.89	4.45	1.10 (0.89-1.35)	0.37

[1]Per protocol analysis excludes any infant not receiving study BCG (SSI-1331) as per their randomisation group (6 in BCG at birth group due to receipt at >24 hours of age (when study BCG was not accessible over a bank holiday weekend) and 2 in BCG at 6 weeks group, who received non-study BCG in the community prior to 6 week follow-up visit) and any blood sample taken outside the protocol timings (see Figure A1). BCG: Bacille-Calmette Guerin, GMR: Geometric Mean Ratios, CI: Confidence interval, IL-6: interleukin-6, TSAT: Transferrin saturation.

Figure A9. Geometric mean ratios of stimulated pro-inflammatory cytokine production and inflammatory-iron parameters comparing infants randomised to BCG at birth vs. BCG at 6-weeks by sex, at the '6-weeks, 5 days post-BCG in the delayed group' time-point.

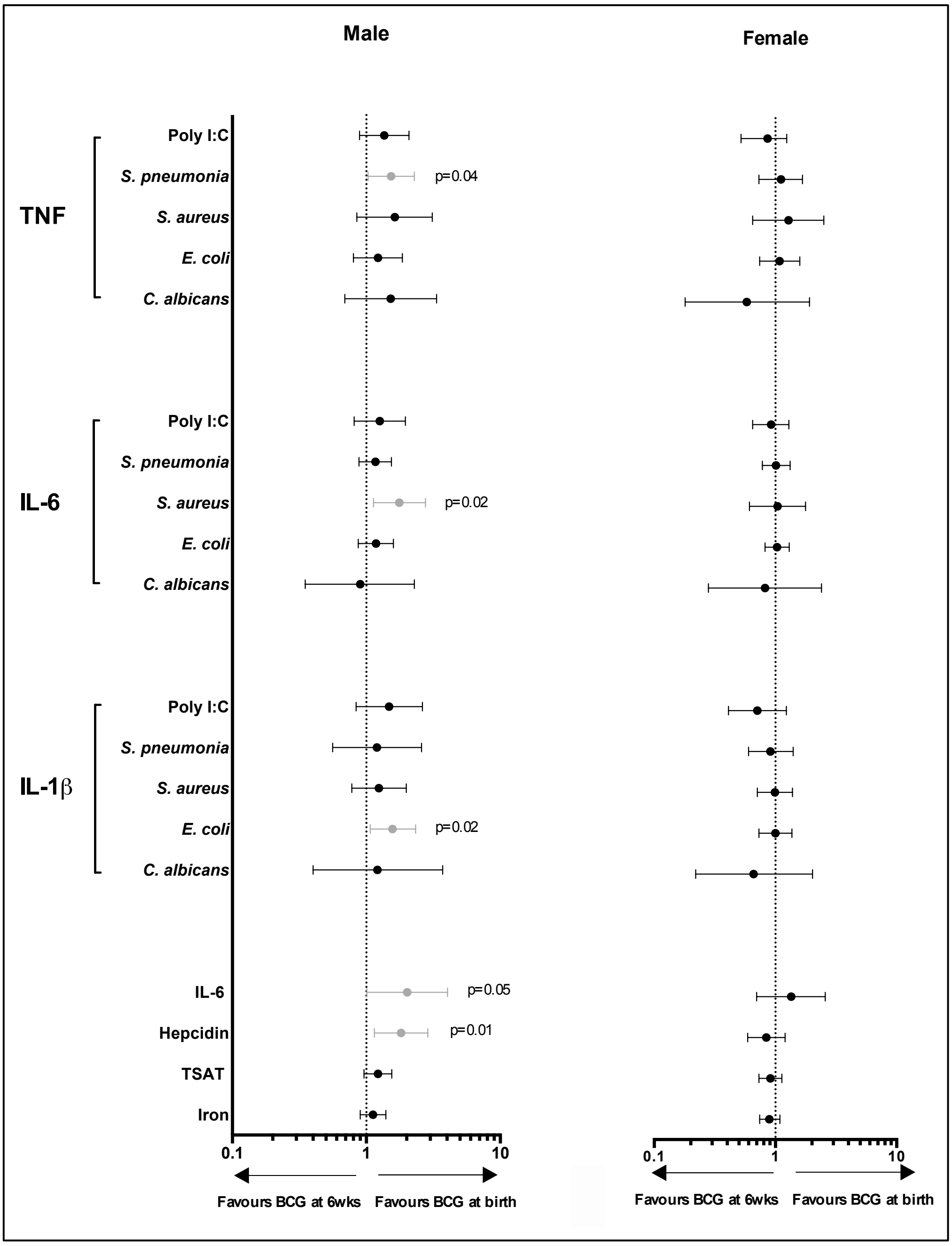

Geometric mean ratios (95% confidence intervals) of a) pro-inflammatory cytokine production measured by ELISA, following 24 hour stimulation of whole blood with bacterial/fungal whole killed pathogens, a viral-type stimulant (Poly I:C) and positive control (PPD) b) circulating inflammatory iron parameters, by BCG status. A geometric mean ratio >1 indicates that levels were highe in the BCG at birth group. Comparisons reaching statistical significance are shown in grey. BCG: Bacille-Calmette Guerin, GMR: geometric mean ratio, TNF: tumour necrosis factor, IL-6: interleukin-6, IL-1β: interleukin-1beta, PPD: purified peptide derivative, Poly I:C: polyinosinic:polycytidylic acid, S. pneumoniae: Streptococcus pneumoniae, S. aureus: Staphylococcus aureus, E.coli: Escherichia coli, C.albicans: Candida albicans, TSAT: transferrin saturation, wks:weeks

Title: Does Neonatal BCG Immunisation Provide Protection Against Heterologous Invasive Infectious Disease by Stimulating the Innate Immune System?

ISRCTN: 59683017

Names of applicants: 1) Dr Sarah Prentice
2) Dr Stephen Cose

Position of applicants:

1) Wellcome Trust Clinical Fellow, London School of Hygiene and Tropical Medicine, UK
2) Senior Immunologist, MRC/UVRI Uganda Research Unit on AIDS, Entebbe, UG
Lecturer in Immunology, London School of Hygiene & Tropical Medicine, London, UK
Hon. Senior Lecturer, Makerere University College of Health Sciences, Kampala, UG

Sponsor

Professor Alison M. Elliott,
Programme Head, MRC/UVRI Uganda Research Unit on AIDS, Entebbe, UG
Professor of Tropical Medicine, London School of Hygiene & Tropical Medicine, London,

Primary Supervisor

Dr Stephen Cose
Senior Immunologist, MRC/UVRI Uganda Research Unit on AIDS P.O Box 49, Entebbe, Uganda
Lecturer in Immunology, London School of Hygiene & Tropical Medicine, London, UK
Hon. Senior Lecturer, Makerere University College of Health Sciences, Kampala, UG

Secondary Supervisor

Professor Hazel Dockrell
Professor of Immunology
London School of Hygiene & Tropical Medicine, Keppel Street, London WC1E 7HT, UK.

Clinical Trial Sponsor

London School of Hygiene and Tropical Medicine

Collaborating Institutions

Dr Moses Muwanga, Medical Superintendent, Entebbe General Hospital, Entebbe, UG
Dr Robert Asaba, Medical Superintendent, Kisubi Hospital, Kisubi, UG
London School of Hygiene & Tropical Medicine, UK
Dr Alexander Drakesmith, Weatherall Institute for Molecular Medicine, Oxford University, Oxford, UK
Professor Mihai Netea, University Radboud, Nijmegen, The Netherlands

Table of Contents

Summary of proposed work **22**
Background **23**
Evidence for the non-specific effects of BCG immunisation 23
Potential innate immunological mechanisms for the non-specific effects of BCG immunisation 24
Rationale for the study **26**
Aims/Objectives/Hypotheses **26**
Methodology **28**
Study design and sampling strategies 28
Study populations 31
Inclusion criteria 31
Exclusion criteria 31
Consent and recruitment 32
Randomisation 33
Blinding 33
Main outcome measures 34
Data collection methods 35
Study procedures 35
Laboratory methods 36
Sample size determination 38
Ethical considerations 40
Risk-benefit analysis 40
Evaluation of alternative study designs 48
Other ethical considerations 49
Clinical Trial oversight and monitoring 52
Data management **53**
Data entry 53
Analysis plan 54
Potential limitations; anticipated problems **55**
Potential logistical problems 55
Potential study design limitations 56
Significance of the proposed work **57**
Plans for dissemination **58**
Time frame **59**
Budget **59**
References **59**
Appendix 1 .... **65**
Safety reporting procedures 65
Safety reporting flow chart 68

Summary of proposed work

Studies in Guinea-Bissau suggest that BCG immunisation of low birth-weight infants may protect them against a range of non-mycobacterial infectious disease. This heterologous protection appears to be most within the first few weeks of life, but may also persist longer-term. This finding has yet to be replicated in normal birth-weight infants and in different settings. In addition, the biological mechanism for such effects has not been elucidated. We aim to investigate whether BCG immunisation non-specifically enhances the innate immune system, providing short-term protection against heterologous infection, and that 'training' of this response occurs, leading to persistently enhanced innate immune responses to heterologous pathogens.

To investigate this we propose a randomised controlled trial of neonates born at Entebbe Grade B and Kisubi Hospitals, comparing infants that are BCG vaccinated at birth with those vaccinated at 6 weeks of age. This delay is within the longest acceptable delay (up to 8 weeks) defined by WHO. Comparison of intervention groups shortly after immunisation and at distant time-points will be carried out to assess: 1) innate cytokine levels following *in vitro* stimulation with various pathogens and 2) inflammatory alterations in the hepcidin-iron axis. The potential for BCG-induced epigenetic modification of monocytes to produce long-term up-regulation of the innate immune response against heterologous pathogens will also be investigated. Clinical follow-up of all infants will occur until 10 weeks of age.

Discovery of a broadly protective effect of neonatal BCG immunisation would have profound implications for public healthcare policy both in Uganda and worldwide. This project could provide evidence to prioritise BCG immunisation for all infants on the first day of life, an EPI goal that is yet to be met. Evidence of protection against heterologous pathogens would also provide important advocacy against the termination of BCG's use in favour of other anti-tuberculous immunisations.

1. Background

Bacillus Calmette-Guerin (BCG) immunisation, the only currently available tuberculosis (TB) vaccine, is one of the most frequently administered immunisations worldwide with more than 100 million children receiving it per year.[1] Although it provides protection against severe forms of TB in children, it has variable efficacy against adult pulmonary disease, with protection generally poor in high-risk areas such as sub-Saharan Africa and Asia.[2] There are currently concerted efforts in the scientific community to improve anti-TB protection by either enhancing existing BCG immunisation strategies (e.g. altering strain[3] or delaying administration past the neonatal period to allow maturation of immune responses[4-7]) or by developing an alternative vaccine.[8]

However, BCG may have other beneficial effects beyond protection against TB. There is some evidence to suggest that BCG immunisation in neonates may protect them against a variety of heterologous pathogens, therefore reducing mortality to a greater extent than by protection against TB.[9] It is important to fully interrogate this possibility as any new vaccine or BCG schedule would need to be evaluated in terms of overall benefit to recipient, rather than in terms of TB-specific protection alone.

Evidence for the non-specific effects of BCG immunisation

The potential for Bacillus Calmette-Guérin (BCG) immunisation to protect infants against non-mycobacterial infectious disease was first suggested following its introduction more than 80 years ago. Controlled trials including more than 46,500 infants, carried out in the 1940's and 50's in the USA and UK, showed on average a 25% (95% CI 6-41%) reduction in all cause mortality in children receiving BCG immunisation compared to those not receiving BCG.[10-14] This reduction was noted to be larger than the reduction in rates of TB, but this was dismissed as secondary to other public health improvements occurring co-incidentally. Multiple animal studies have also revealed BCG's potential to protect against non-mycobacterial pathogens including bacteria,[15-19] viruses,[20-22] fungi[23, 24] and protozoa.[25-27]

Only one randomised controlled trial investigating the potential non-specific protective effects of BCG immunisation in humans exists.[28] In this trial of low birth-weight infants in Guinea-Bissau, subjects randomised to receive BCG immunisation at birth had a 45% lower mortality rate (MRR 0.55 (0.34,0.89)) in the first 2 months of life than infants who had BCG immunisation delayed to, on average, 6 weeks of age. The reduction in deaths was due to protection from all-cause febrile illness, respiratory tract infections and diarrhoea, but not against tuberculosis (verbal autopsy data). Although the benefit was strongest within the first few weeks of life, a weaker effect appeared to persist to age 12 months (MRR 0.83 (0.63-1.08)).[28] These randomised controlled trial data support earlier, controversial, observational data collected by the same group, which showed that children with BCG immunisation as their last immunisation had approximately half the rate of death and hospital admissions for respiratory and diarrhoeal illness, than

infants who had DTP as their last immunisation.[29-35] Although exciting, this randomised controlled trial provides insufficient evidence to accept the global applicability of the non-specific effects of BCG immunisation as it was conducted in a selected population of high risk infants (low birth-weight) and in a country with one of the highest infant mortality rates in the world (98 per 1000 live births). [36] The evidence for generalizability is further limited by a lack of recent observational studies conducted outside of Guinea-Bissau to assess the potential non-specific effects of BCG immunisation.

A biological mechanism for the proposed non-specific protective effects of BCG immunisation is also lacking, limiting the acceptance of the theory by the scientific and public health communities. Studies into the hypothesis that BCG immunisation might skew the adaptive immune response from the Th2 dominant environment of early neonatal life, toward a more protective Th1 environment, have been inconclusive.[37-39] The evidence from Guinea-Bissau shows that any putative immunological mechanism would need to be:

- Effective at birth despite the immature neonatal immune system
- Rapidly inducible (most protection at <1wk post-immunisation)
- Active against a range of pathogens

These features suggest that BCG mediates its non-specific effects by stimulating the innate immune system. If long-term non-specific protection also occurs following BCG immunisation, a mechanism is required to explain this ‘training’ of the innate immune system to respond, in a persistently amplified manner, against heterologous pathogens.

Potential innate immunological mechanisms for the non-specific effects of BCG immunisation

1. *Enhancement of pro-inflammatory cytokine production*

The generalised pro-inflammatory effects of BCG immunisation have long been recognised, with its use as immunotherapy against bladder cancer[40] and as an adjuvant in novel vaccine design.[41] BCG immunisation of naïve European adults has been shown to lead to enhanced production of the pro-inflammatory cytokines TNF-α, IL-1β and IFN-γ after *in vitro* non-specific stimulation.[24] Few studies exist investigating alterations in cytokine production to heterologous stimulants following neonatal BCG immunisation.[3, 42, 43] The few that have been done have generally shown moderate enhanced cytokine production to heterologous pathogen challenge, but they all have limitations. Each of these studies have focused more on adaptive cytokines, using a 6 day *in vitro* stimulation protocol, which is sub-optimal for the quantification of innate cytokine production. No studies exist where samples have been collected prior to 5 months of age, thus potentially missing early non-specific effects of BCG immunisation. Lastly, *in vitro* stimulants used in previous studies have been antigens (e.g. LPS or tetanus toxoid) and not whole organisms, potentially excluding the effect of other important pattern recognition receptor pathways. As part of this proposed study we aim to address these issues by focusing on the impact of BCG immunisation on innate cytokine production, conducting overnight stimulation using non-mycobacterial whole organism stimulants, on blood samples taken before 10 weeks of age.

1. *Enhancement of the inflammatory iron pathway*

Iron supply is critical for the virulence of most pathogens.[44] Plasma hypoferraemia profoundly inhibits growth of bacteria,[45, 46] viruses,[47] protozoa[48-51] and fungi.[52, 53] The iron regulatory hormone hepcidin is induced by IL-6, leading to hypoferraemia as part of the innate acute-phase response. Guinea-pig models reveal that BCG immunisation induces a rapid bacteriostatic hypoferraemia,[54] although involvement of the IL-6/hepcidin pathway has never been studied. BCG-induced reduction in serum iron might be particularly beneficial in the first few days of life, as transition from fetal circulation leads to a relatively iron-overloaded state. The 20-fold increase in *E.coli* sepsis occurring after administration of iron-dextran to Polynesian neonates shows the influence of iron on neonatal vulnerability to infection.[55, 56] To our knowledge, no studies exist investigating the influence of BCG immunisation on the human iron-inflammatory pathway. As part of this study we will investigate alterations to the inflammatory iron axis induced by BCG immunisation in neonates, as a potential effector mechanism for heterologous protection.

2. *Monocyte epigenetic modification*

Enhancement of pro-inflammatory cytokine production and/or potentiation of the iron inflammatory pathway may provide short-term protection against heterologous pathogens by a bystander effect of inflammation induced by BCG. For longer-term enhanced protection a mechanism whereby BCG immunisation trains the innate immune system to respond in a persistently amplified manner when challenged by heterologous pathogens is required. Epigenetic modification of histone proteins at the promoter region of innate cytokines can persistently up-regulate their transcription in response to inflammatory stimuli. BCG immunisation of naïve adults has been shown to produce tri-methylation of histone-3 lyseine-4 at the promoter region of TNF-α, IL-1β and IFN-γ in monocytes.[24] This led to enhanced cytokine production following *in vitro* stimulation with the heterologous pathogens *S. aureus* S. pneumoniae and *C. albicans*, which persisted to at least 3 months post-immunisation. The ability for BCG immunisation to produce similar monocyte epigenetic modification in neonates, and thus longer-term up-regulation of innate immune effector mechanisms in response to heterologous pathogen challenge, has not yet been studied. We therefore aim to investigate whether BCG-induced epigenetic modification of monocytes persistently up-regulates the pro-inflammatory cytokine response to heterologous pathogens, training the innate immune system to provide long-term amplified protection, through potentiation of the general acute-phase response, enhancement of IL-6 mediated hypoferraemia, or both.

Thus, we propose to conduct a randomised controlled trial, comparing BCG administration at birth with administration at 6 weeks of age. We will use this design to interrogate alterations in the innate immune response to heterologous pathogens, as well as comparing clinical illness end-points between the two groups.

3. Rationale for the study

Global recognition of the potential of a non-specific protective effect afforded by BCG immunisation has been limited due to a paucity of randomised controlled trial data, along with the fact that most research on the topic has been conducted by one research group in one location, and also the lack of a plausible biological mechanism to explain the observed effects. By carrying out this randomised controlled trial in Uganda and investigating the impact of BCG immunisation on the innate immune system in neonates, we aim to address all of these issues. Interrogation of the possible heterologous protection afforded by BCG immunisation is important to ensure that any new anti-tuberculosis vaccine or alteration in timing of BCG administration is evaluated in terms of overall benefit to recipient, rather than in terms of TB-specific protection alone. This project could also provide evidence to prioritise BCG immunisation for all infants on the first day of life, an EPI goal that is yet to be met.

4. Aims/Objectives/Hypotheses

Our goal is to understand the impact of neonatal BCG immunisation on the innate immune response to heterologous pathogens.

Overall Null Hypothesis: Neonatal BCG vaccination has no impact on the innate immune systems response to heterologous pathogens

Overall Alternative Hypothesis: Neonatal BCG vaccination enhances the innate immune systems response to heterologous pathogens, which may lead to increased protection against invasive infectious disease.

Aims

1. To determine whether BCG immunisation at birth alters the innate immune response to heterologous pathogens in the short term.
2. To determine whether BCG immunisation at birth alters the innate immune response to heterologous pathogens in the longer term (~6 weeks)
3. To determine whether BCG immunisation given at age 6 weeks has similar short and longer-term effects on the innate immune response to heterologous pathogens compared to BCG immunisation at birth.
4. To obtain preliminary data upon the effect of BCG on neonatal susceptibility to invasive infections in Ugandan infants.

These aims will be achieved using a single-blind, randomised controlled trial comparing infants who are BCG vaccinated at birth with those vaccinated at age 6 weeks. Aims 1, 2 and 3 will be addressed using sub-studies to interrogate three different elements of the innate immune system. These sub-studies have the following specific hypotheses and objectives:

Sub-study 1: Pro-inflammatory cytokine production

Null hypothesis: BCG immunisation produces no alteration in the innate pro-inflammatory milieu in response to heterologous pathogen challenge in the short or longer-term

Alternative hypothesis: BCG immunisation produces a generalised potentiation of the innate pro-inflammatory-milieu in response to heterologous pathogen challenge in the short and longer-term

Primary Objectives: Cross-sectional comparison of IL-1β, IL-6, IL-10, TNF-α and IFN-γ cytokine levels following overnight *in vitro* stimulation with *S.aureus, S.pneumoniae, E.coli, FEC (Influenza, Epstein Barr Virus and Cytomegalovirus)* and *C.albicans* between the two intervention groups*:*

1. Shortly after birth intervention (BCG immunisation/no immunisation) **Aim 1**
2. Six weeks post-birth intervention (immediately prior to first dose of primary immunisations) **Aim 2**
3. Shortly after 6 week intervention (BCG immunisation/no immunisation) **Aim 3**
4. Three weeks post-6 week intervention (immediately prior to second dose of primary immunisations) **Aim 3**

Secondary objectives: Longitudinal analysis of within-infant changes in innate cytokine production following *in vitro* stimulation with the above pathogens.

Sub-study 2: Inflammatory mediated changes in iron status

Null hypothesis: BCG vaccination does not stimulate an IL-6/hepcidin mediated hypoferraemia in the short or longer term.

Alternative hypothesis: BCG vaccination produces an IL-6/hepcidin mediated hypoferraemia that enhances protection against heterologous pathogens in the short and longer-term.

Primary Objectives: Cross-sectional comparison of transferrin saturation and hepcidin levels between the two intervention groups:

1. Shortly after birth intervention (BCG immunisation/no immunisation) **Aim 1**
2. Six weeks post-birth intervention (Shortly after first dose of primary immunisations) **Aim 2**
3. Shortly after 6 week intervention (BCG immunisation/no immunisation) **Aim 3**
4. Three weeks post-6 weeks intervention (Shortly after second dose of primary immunisations) **Aim 3**

Secondary objectives:

- Cross-sectional comparison of serum iron, total iron binding capacity, ferritin, transferrin, haemoglobin and red cell parameters at the above time-points.
- Longitudinal analysis of within-infant changes to iron status following *in-vivo* non-specific stimulation (provided by primary immunisations)

Sub-study 3: Monocyte epigenetic modification

Null hypothesis: BCG vaccination does not induce epigenetic modification of monocytes to produce persistent up-regulation of the pro-inflammatory cytokine response to heterologous pathogens.

Alternative hypothesis: BCG vaccination induces epigenetic modification of monocytes which persistently up-regulates the pro-inflammatory cytokine response to heterologous pathogens, training the innate immune system to provide long-term amplified protection.

Primary objectives: Cross-sectional comparison of monocyte histone-3, lyseine 4 tri-methylation at the promoter region of pro-inflammatory cytokines between the two intervention groups:

1. Shortly after birth intervention (BCG immunisation/no immunisation) **Aim 1**
2. Six weeks post-birth intervention (Immediately prior to first dose of primary immunisations) **Aim 2**

Secondary objectives: Longitudinal analysis of within-infant changes in monocyte epigenetic modification.

Clinical outcome measures from all 3 sub-studies will be combined to address **Aim 4.**

5. Methodology

Study design and sampling strategies

This study will be a single-blind randomised controlled trial comparing infants that are BCG vaccinated on the first day of life with those that are BCG vaccinated at 6 weeks of age. Infants will have placental cord blood and two 2ml venous blood samples taken to investigate immunological parameters. Blood samples taken before 6 weeks of age will compare heterologous innate immune responses between BCG vaccinated and naïve infants. Blood samples taken between 6 and 10 weeks of age will compare heterologous innate immune responses between early and delayed BCG vaccinated infants, investigating whether there is a critical period for the non-specific effects of BCG immunisation. Interrogation of pro-inflammatory cytokine production, the iron-inflammatory response and epigenetic changes will be carried out using separate sub-studies,

utilising the same overall design. All infants will be clinically followed up to 10 weeks to allow combined analysis of clinical end-points across the three sub-studies.

Figure 1 displays the intervention and blood sampling time-points for the cytokine and epigenetic sub-studies. All infants will receive immunisations, other than BCG, according to the Ugandan National vaccination schedule.

Figure 1. Intervention and blood-sampling time-points. Cytokine and epigenetic sub-studies

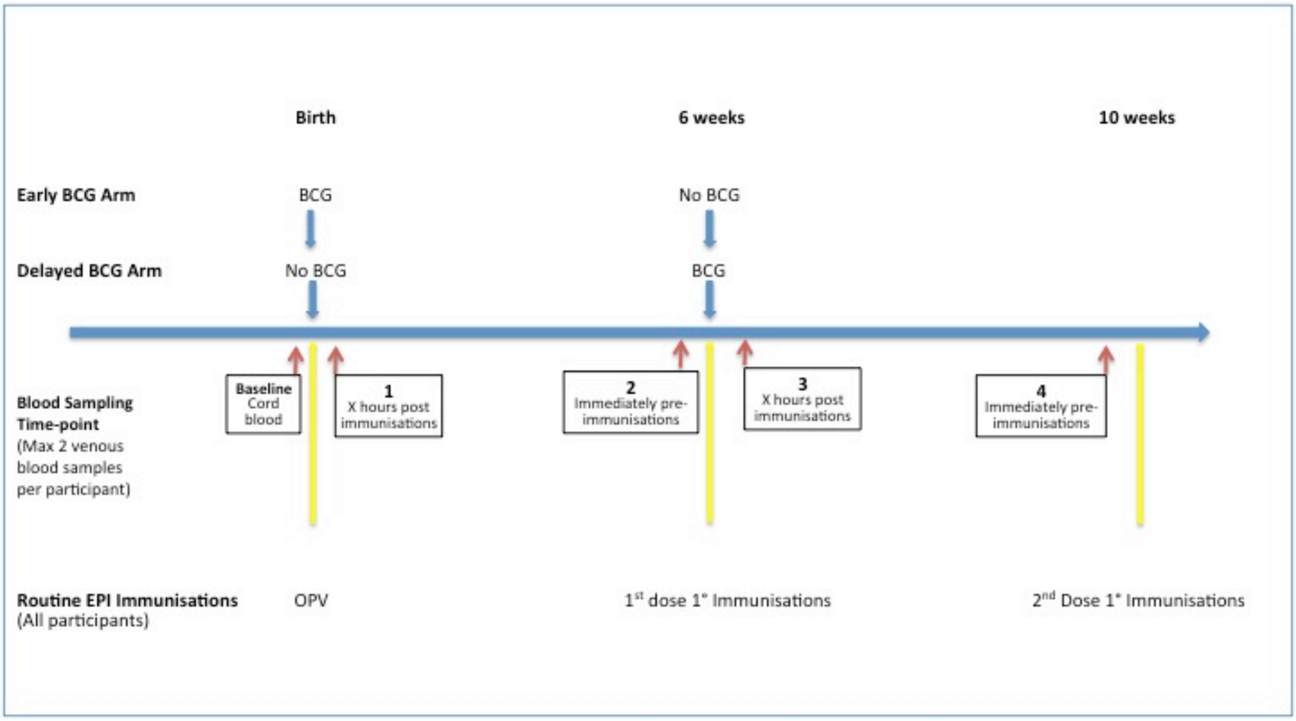

In these two sub-studies the blood-samples investigating the longer-term effects of BCG immunisation (samples 2 and 4) will be taken immediately prior to receipt of primary immunisations, to avoid their potential confounding effect on innate immune responses measured *in vitro*. The systemic nature of the iron-inflammatory response (hepcidin being produced mostly in the liver) precludes *in vitro* non-specific stimulation. Thus, longer-term blood samples in this study will be taken after receipt of primary immunisations, which will act as an *in vivo* non-specific stimulant. BCG immunisation in the delayed immunisation group will therefore be given after this blood sample has been taken. Figure 2 displays the intervention and blood sampling time-points for the iron sub-study. The three immunological sub-studies will be run sequentially, not concurrently, to avoid confusion.

Figure 1. Intervention and blood-sampling time-points. Iron sub-study

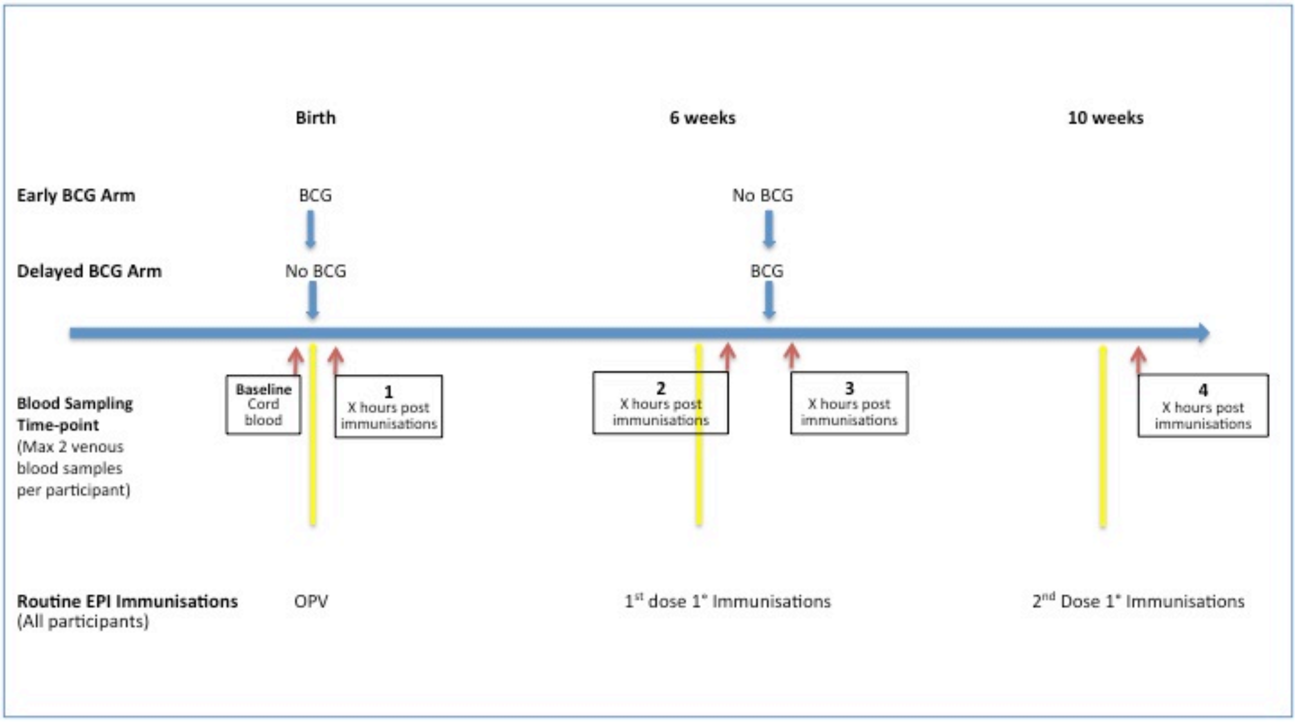

The optimal acute blood-sampling time 'X' is currently being defined by a pilot study. This study compares the levels of IL-6, hepcidin and iron parameters on day 2 and day 4 of life in neonates randomised to receive BCG immunisation either before or after blood samples (day 1 or day 4 respectively). This pilot study is due to be completed by December 2013. A further study to investigate the kinetics of changes following BCG vaccination, and thus help inform optimal blood sampling time-point, particularly for the cytokine and epigenetic sub-studies, will be carried out by a collaborator Dr Tobias Kollmann at the University of British Columbia using samples from the iron sub-study. These investigations will use a functional 'omics' approach to gain information regarding proteomic, metabolomic and transcriptomic changes post-BCG vaccination on small volume of excess stored blood.

As it is unlikely to be acceptable to obtain four blood-samples per infant, twice the statistically required sample-size will be recruited and each infant bled at only two time-points. Bleeding time-points will be randomly assigned in pairs to allow secondary longitudinal analysis of within-infant changes in innate immune parameters.

Stool samples will also be collected from all study subjects at week 6 and week 10 (prior to receipt of primary immunisations), to investigate alterations in gut microbiome between the two intervention groups. Nasal swabs will also be stored from all infants at weeks 1, 6 and 10. This will allow comparison of rates of Streptococcus pneumonia carriage to be compared by quantitative PCR between the two intervention groups. We currently do not have funding to complete the microbiome and nasal pneumococcal carriage studies, and if we are successful in securing further funds to conduct these studies, an amendment to this protocol will be submitted.

Study populations

The study population will be infants born to consenting mothers at Entebbe Grade B and Kisubi Hospitals.

Inclusion criteria

Neonates born to women delivering in the hospital will be eligible for inclusion if:

a) Mothers consent for participation
b) They reside in the study catchment areas
c) Mothers are HIV negative (based on records available from antenatal care received during this pregnancy)
d) The birth was sufficiently uncomplicated to allow the neonate to be discharged directly home from hospital with no infant admission or treatment for complications
e) The neonate is of a gestational age and birth weight to allow discharge directly home from hospital (no requirement for supplemental oxygen or feeding)

Exclusion criteria

Neonates will be excluded from the study if:

a) Cord blood is not obtained
b) They have major congenital malformations
c) The infant is clinically unwell, as judged by a midwife
d) Known maternal TB or active TB within the family (based on direct questioning of mother during recruitment).
e) Maternal or family member positive for any TB screening symptoms:
    - Cough > 2 weeks
    - Recent haemoptysis
    - >3kg weight loss in past month
    - Recurrent fevers/chills or night sweats for the past 3 days or more

   (Anybody with 1 or more positive symptoms will be referred to adult services for further assessment for TB)

No specific weight or gestational age limit has been set for this study. Clinical responses to early BCG are suggested to have the greatest effect in infants of the lowest birth-weight,[28] thus it is important to include these infants in data collection. No increased rate of detrimental side-effects or reduction of immunological efficacy has been shown with BCG immunisation of premature infants.[57] Gestational age and birth weight will be documented and adjusted for during analysis.

The exclusion of symptomatically unwell infants at birth is essential for the interpretation of the primary immunological outcomes of this study. The innate immune system is highly likely to be stimulated in unwell infants, resulting in high values for immunological parameters in the baseline cord blood sample. This might confound the interpretation of any subsequent effects of BCG vaccination on the innate immune system. The exclusion of these infants will not affect our ability to study the impact of BCG vaccination on non-tuberculous infectious diseases in the neonatal period as it will only eliminate antenatal infectious pathogen exposures, a time when BCG vaccination of the infant is impossible. Once born, the infants will continue to be regularly exposed to infectious pathogens. This study will therefore assess whether BCG vaccination at birth alters the number of clinically symptomatic infections resulting from these exposures in the first 6 weeks of life.

Consent and Recruitment

Sensitisation of parents to the study will occur during antenatal classes via posters, group discussions and during individual mid-wife led consultations. The principal investigator will be available during group discussions and otherwise by telephone to answer any questions or concerns about the study.

Mothers will be approached for consent by trained midwives when presenting in early labour to Entebbe Grade B or Kisubi Hospitals. The study will be explained in detail verbally and the information sheet provided or read to illiterate mothers. Information sheets will be available in English and Luganda. Consent will also be taken to allow for storage of excess samples and use of data in future research studies. Consent will be obtained from fathers only if mothers request it.

A two-stage procedure with verbal informed consent obtained during labour, to allow for collection of placental cord blood and further full written informed consent after delivery before the mother and baby are discharged from hospital. If written informed consent is not obtained, or the mother changes her mind, the infant will be excluded from the study, and the cord blood sample destroyed. At all follow-up visits study staff will confirm that the mother has understood the study and its requirements, before further procedures are conducted.

Another study run by our group, investigating the impact of maternal tuberculosis infection on infant responses to BCG, will run concurrently with this study[1]. The consent process will be the

[1] MRC funded study 'The impact of maternal infection with Mycobacterium tuberculosis on the infant response to BCG immunisation' Applicants: Dr Stephen Cose, Professor Alison Elliot and Professor Pontiano Kaleebu. Application approved by UVRI SEC 23rd September, 2013. Protocol submitted to UNCST.

same and we may stagger recruitment into each study on alternating weeks to aid even allocation of subjects.

Randomisation

Intervention and blood sampling time-point allocation will be determined by block randomisation, stratified by sex, conducted by the Data Manager using Microsoft Access. Allocations will be concealed within a sealed envelope. Mothers will be asked to choose between a selection of envelopes, to aid understanding of the random nature of the trials intervention.

Any mothers who request BCG vaccination at birth following randomisation and assignment of their child to the 6-week arm will be counseled again about the study design and intent. If they would still prefer for their child to be vaccinated at birth then they will be withdrawn from the study and the reasons for this withdrawal noted. They will then revert to national vaccination care, receiving standard BCG vaccination at birth. We do not feel it is appropriate to maintain these infants in the study, as it would break randomization and is likely to bias the results.

Blinding

This study will be single-blind. Mothers will not be blinded to intervention allocation due to lack of feasibility (BCG immunisation produces a visible reaction) and to reduce confusion if a child lost to follow-up presents to a community immunisation clinic. Immunological investigations will be conducted on blood samples identified only by study number. Intervention allocation code will only be broken once laboratory analysis is complete and the data have been cleaned and locked.

Blinding of investigators performing clinical assessment of children (routine follow-up or illness episode) will be accomplished by placing a plaster over the area corresponding to BCG immunisation site. This will be placed by a nurse not involved in clinical follow-up. If a child is presenting due to concerns about the immunisation site it will be left uncovered and that clinical episode excluded from final analysis. Researchers involved in clinical follow-up will not be responsible for intervention administration.

Main outcome measures

<u>Sub-study 1 – Pro-inflammatory Cytokine Analysis</u>

Primary Outcomes:

- IL-1β, IL-6, IL-10, TNF-α and IFN-γ cytokine levels following *in vitro* stimulation with *S.aureus, S.pneumoniae, E.coli, FEC* and *C.albicans*:
    1. Up to 1 week post-birth intervention

1. Immediately prior to first dose of primary immunisations (6 weeks post-birth intervention)
2. Up to 1 week post-6 week intervention
3. Immediately prior to second dose of primary immunisations (4 weeks post-6 week intervention)

<u>Sub-study 2 – Inflammatory Iron Status</u>

Primary Outcomes:

- Hepcidin levels and transferrin saturation:
    1. Up to 1 week post-birth intervention
    2. Up to 1 week post first dose of primary interventions
    3. Up to 1 week post-6 week intervention
    4. Up to 1 week post second dose of primary interventions

Secondary Outcomes:

- Serum iron, total iron binding capacity, ferritin, transferrin, haemoglobin and red cell parameters at the above time-points.

<u>Sub-study 3 – Monocyte Epigenetic Modification</u>

Primary Outcomes:

- Tri-methylation of Histone-3, Lyseine-4 at the promoter regions of pro-inflammatory cytokines in monocytes:
    1. Up to 1 week post-birth intervention
    2. Immediately prior to first dose of primary immunisations (6 weeks post-birth intervention).

<u>Combined Studies – Clinical Illness Events</u>

Primary Outcomes:

- Physician-diagnosed Invasive Infectious Disease

Secondary Outcomes:

- Parent reported Invasive Infectious Disease
- Blood Culture positive Invasive Infectious Disease
- Death

Data Collection Methods

Consent and recruitment paper forms will be completed by study midwives and entered into the study database by the data entry clerk. Clinical review and illness episode questionnaires will be

collected by direct electronic capture by the research nurse, clinical officer and/or principal investigator. Laboratory assay results will be transferred to the database electronically. Paper printouts of results will be issued and checked against the electronic database to ensure integrity of transfer.

Data from paper forms will be double entered into Microsoft Access by the principal investigator and a trained data-entry clerk to ensure quality. Hard copies will be kept securely in the MRC/UVRI archive and will be available in case of any data discrepancies or queries.

Electronic capture data will be entered directly by the research nurse and/or clinical officer. Electronic forms will be designed using a combination of drop-down menus to limit inputs to valid answers, and range checks to ensure research personnel are alerted if invalid entries are used. Electronic capture will be validated at the beginning of the study by co-documentation with paper forms and comparison. During the study, electronic data will be uploaded on a daily basis and sent in an encrypted form to the data management team. This will be stored in an interim data management file. Data will only be uploaded onto the master database once any queries highlighted by quality control checks have been resolved. This process has been successfully used in a number of studies in the Unit.

Study Procedures

1. Mothers consented in early labour by a trained midwife.
2. Cord blood collected from the umbilical vein by the midwife using needle and syringe
3. Consent and eligibility for full participation confirmed by the midwife.
4. Randomisation of infants by mothers choosing from sealed, randomly ordered envelopes (stratified by sex). Intervention group and blood sampling time-points allocated.
5. Baseline maternal demographic and infant birth details form completed by midwife.
6. Birth intervention administered as soon as possible (<24 hours) after birth: BCG + OPV or OPV alone.
7. Mother and infant pair driven home from hospital when ready for discharge. Contact details confirmed. Personal Participant Plan card given to mother with clinic visit dates. A copy of the Personal Participant Plan will be transferred to the research clinic for secure storage in the subjects file.
8. Participant seen in study clinic for:
    - (a) Venous blood sampling x 2 (2ml per sample)
    - (b) Routine immunisations (first and second dose of primary immunisations)
    - (c) 6 week intervention (BCG or nothing)

These procedures will be conducted by a research nurse who will also document anthropometry and routine observations by direct electronic capture. A stool sample and nasal swab will also be collected from each infant prior to first and second dose of primary immunisations.

1. The participant will be reviewed at every clinic visit by a clinician (clinical officer or principal investigator) who is blinded to intervention allocation. Parent recall of illness episodes and objective clinical assessment of the participant will be documented by direct electronic capture.
2. All children will be actively followed up by weekly telephone contact with the mother to ensure they remain well, and arrangements made for the child to be assessed in clinic if there are concerns. If a mother is not contactable on two consecutive days a field worker will be dispatched to locate and review the child.
3. All participants will be reviewed by a clinician, blinded to intervention allocation, when presenting to the clinic with an illness episode. Illness episode data including history, examination findings, investigation, treatment and outcomes will be documented by direct electronic capture. Patients requiring admission will be stabilised in the clinic and transferred to the paediatric ward at Entebbe Grade B Hospital. In addition to routine investigations available at Entebbe Grade B Hospital, where appropriate, infants will have blood taken for blood cultures and CRP. These will be analysed at UVRI laboratory and results fed back to the attending clinician. Management of admitted children will be carried out by the paediatric staff at Entebbe Grade B Hospital, with daily review and documentation of outcomes by research staff.
4. Initial processing of blood samples will occur at UVRI laboratory by a laboratory technician or the principal investigator. Please see below for laboratory methods.

13. Study participation is complete after final clinical follow-up at 10 weeks of age.

Laboratory methods

Cytokine analysis

1) *Overnight Whole Blood Stimulation* - Whole blood diluted 1:4 in sterile RPMI will be stimulated overnight at 37° with heat-killed *S. aureus, S. pneumoniae, E. coli, FEC* and *C. albicans.* These stimulants have been chosen as a) they are the most common pathogens isolated from septic neonates in Uganda[58] and b) they represent a range of pathogen types. The stimulants will be provided by Professor Netea. PPD and medium alone will be used as positive and negative control stimulants respectively. Supernatants will be harvested and frozen prior to ELISA. Stimulation will be conducted in duplicate to provide sufficient supernatant.
2) *Pro-inflammatory cytokine ELISA's.* Levels of pro-inflammatory cytokines IL-1β, IL-6, IL-10, TNF-α and IFN-γ in supernatants derived from whole blood stimulation assays will be

measured by ELISA (Becton-Dickson, UK). ELISA's will initially be run in singlecate but will be repeated if sample volume and funding allows.

Inflammatory iron analysis:

1) *Hepcidin analysis* – Serum hepcidin status will be measured on frozen lithium-heparin samples using Hepcidin-25 Bachem competitive ELISA kits. This analysis will be conducted at the UVRI laboratories in Entebbe in duplicate.
2) *Iron parameters* – Serum iron status will be measured on frozen lithium-heparinised samples using an automated analyser (Cobas Integra 400-Roche Diagnostics). This measures total serum iron by calorimetric measurement. Unsaturated Iron Binding Capacity (UIBC) is measured by adding a known quantity of ferrous iron to the serum, reducing it with ascorbate and measuring it with FerroZine reagents. Total Iron Binding Capacity (TIBC) is calculated from the sum of serum iron and unsaturated iron binding capacity. Transferrin saturation is calculated by dividing the serum iron by TIBC. Serum ferritin is measured by particle enhanced immunoturbidimetric assay. This analysis will be conducted in the UK due to lack of availability of a suitable analyser in Entebbe.
3) *Red Cell Parameters* – Red cell parameters (haemoglobin, mean cell volume, mean cell haemoglobin concentration, reticulocyte count, haematocrit) will be measured from EDTA blood by automated machine at MRC CDLS. Any parameters outside the reference range will be confirmed with a manual blood film and followed-up clinically if appropriate.

Epigenetic modification:

1) *Peripheral Blood Mononuclear Cell (PBMC) Isolation* - PBMC isolation will be conducted by centrifugation of EDTA blood with Ficoll Paque.
2) *Chromatin Immunoprecipitation (ChIP)-sequencing* – Quantification of histone-3, lyseine 4 tri-methylation (H3K4me3) at the promotor regions of pro-inflammatory cytokines will be conducted by chromatin immunoprecipitation sequencing. Briefly, cells are lysed and DNA harvested. DNA is fragmented by sonication and de-crosslinked before being eluted past magnetic beads coupled to H3K4me3 antibodies. The bound DNA is then purified, sequenced and quantified by quantitative PCR. DNA extraction from adherent PBMC's will be conducted in UVRI laboratories and shipped to Professor Mihai Netea's group in The Netherlands for completion of ChIP-sequencing. This highly specialist procedure is currently not feasible in Uganda.

Any excess blood volume remaining after the above studies will be stored to enable future use, subject to further ethical approval.

Additional studies

1. 10 microliters of plasma from the unvaccinated infants samples will be used to test for tuberculosis specific antibodies. This will be compared with vaccinated samples from a different study (The Infant BCG Study) to investigate whether BCG vaccination alters the rate of decay of maternally derived antibody titres.
2. Stored samples from 100 infants (50 in each arm) will be used to carry out DNA sequencing (DNA extracted from cell pellets) and gene expression analysis (RNA sequencing from peripheral blood, stabilised in RNAlater). This will be used to identify differential gene expression and expression quantitative trait (eQTL) signatures between individuals that received BCG at birth compared with delayed to 6 weeks.

Sample size determination

The overall sample size is the summation of the required participant numbers for each sub-study:

Cytokine analysis: n=240

Due to paucity of published data in this area, an approach based on standard deviation (SD) change in average population cytokine levels has been used. 48 subjects per intervention group (BCG immunisation at birth or at 6 weeks of age) will be needed at each time point to show a 0.66 SD change in average population cytokine levels at 90% power and 5% significance. 60 infants per intervention group per time point will be recruited to allow for attrition. Thus, 120 infants will need to be bled at each of the 4 time points. Each child will be bled at 2 time points so 240 infants will need to be recruited in total:

$$\frac{\text{60 infants x 2 intervention groups x 4 time-points}}{\text{Each child bled at 2 time-points}} = \text{240 infants recruited}$$

Iron analysis: n=240

Sample size determination was performed using transferrin saturation (TSAT), as it is the only primary outcome parameter currently of clinical relevance. Average neonatal TSAT in low-income settings is 55%.[59] Substantial responses in this end-point would be required to support its role on the causal pathway of the large (claimed) protective effects of BCG against heterologous pathogens. 50 infants in each group at each time point will be needed to show a 30% reduction in transferrin saturation (reduction to average adult levels in low income settings) at 90% power and 5% significance. 60 subjects will be recruited to each intervention group at each time point to allow

for attrition. Thus, 120 infants will need to be bled at each of the 4 time points. Each child will be bled at 2 time points so 240 infants will need to be recruited in total:

$$\frac{\text{60 infants x 2 intervention groups x 4 time-points}}{\text{Each child bled at 2 time-points}} = \text{240 infants}$$

Epigenetic analysis: n=80

The only previous study in this area to base sample size calculations on was a study in adults, requiring 20 subjects per intervention arm. 40 subjects will be recruited to each intervention arm (BCG immunisation at birth or at 6 weeks) to allow for attrition and also due to the requirement of 2ml blood for epigenetic analysis, which is unlikely to be obtained for all subjects. Inadequate blood volumes obtained will be stored for future transcriptomic analysis. Each infant will be bled at 2 time-points. As epigenetic analysis will be restricted to the first 2 sampling time-points, 80 subjects will be recruited in total (40 per intervention group).

$$\frac{\text{40 infants x 2 intervention groups x 2 time-points}}{\text{Each child bled at 2 time points}} = \text{80 infants}$$

Overall sample size: n=560

Combined analysis of clinical end-points from all 3 sub-studies will be conducted as secondary analysis. Based on data from a previous study in Entebbe[60] we expect 80% power to detect a ≥40% reduction in physician diagnosed invasive infections with $p<0.05$. The effect of BCG is unlikely to be this pronounced, but this preliminary data should provide sufficient evidence to determine whether application for funding to expand the cohort would be valuable.

Ethical considerations

Risk-benefit analysis of altering BCG vaccination from birth to 6 weeks of age

As BCG vaccination at birth is the standard of care in Uganda, below we detail a risk-benefit analysis for altering this standard and delaying BCG vaccination to 6 weeks of age. It should be noted that the most recent investigation of age at BCG vaccination in Uganda (2000)[61] showed that 46% of infants do not receive BCG vaccination by 12 weeks of age. Thus, as all of our study infants will be BCG vaccinated by 6 weeks of age, our study would not be worsening population wide vaccination status in Uganda

Benefits of delaying BCG vaccination to 6 weeks of age

- *Reduced risks of BCG induced complications*

BCG vaccination can induce a number of complications including abscesses (1/100), supurative lymphadenitis (1/1000) and osteomyelitis (1/3000).[62] These risks are estimated to be reduced by approximately one third if vaccination is delayed past the neonatal period.[6] Thus if all 560 infants in our study were vaccinated at birth we would expect approximately:

5.6 cases of BCG induced abscess formation

0.56 cases of supurative lymphadenitis

0.19 cases of BCG induced osteomyelitis

Thus if the risk of these complications is one-third less in the half of infants vaccinated at 6 weeks we would expect to save **1 case of BCG abscess formation, 0.1 cases of supurative lymphadenitis and 0.03 cases of osteomyelitis.** Although small in real numbers, it should be noted that the estimated reduction in BCG induced complications in our study is higher than the estimated risk of acquiring latent or active TB (see below).

- *Potentially enhanced long-term protection against TB*

It is well documented that BCG immunisation has variable efficacy worldwide and clinical protection remains sub-optimal in most areas where neonatal BCG immunisation is recommended.[63] The recommendation for administration of BCG vaccination at birth is a historical one, based on the findings of some benefit against TB compared to un-vaccinated children and for logistical reasons as the majority of infants are reviewed by a health care professional soon after birth.[64] The optimal timing for BCG administration with respect to best long-term protection against TB has never been determined.[65] Several research groups have hypothesized that delaying BCG immunisation to a time when the immune system is more mature may produce enhanced immune responses and therefore better clinical protection against TB.[4-7, 66, 67] A variety of lines of immunological evidence suggest that delaying BCG immunisation to between 1 and 3 months of age results in enhanced anti-tuberculosis immune responses, including larger scar formation,[5, 6] enhanced tuberculin[5]/PPD[6] reactivity *in vivo* and longer duration of PPD[66]/tuberculin[5] reactivity. One study did not find significant differences in IFN-γ secretion or proliferative response when BCG vaccination was delayed past the neonatal period,[68] however it is suggested that measurement of IFN-γ underestimates the complexity of the BCG-induced Th1 response.[4] A more comprehensive study investigating other Th1 cytokines including TNF- α and IL-2, showed enhanced type 1 cytokine and memory T-cell responses when BCG vaccination was delayed to 10 weeks of age[4]. A further small study also showed a trend toward higher *in vitro* cytotoxicity and proliferative immune responses when BCG vaccination was delayed to 10 weeks.[7] **It is difficult to quantify the degree of clinical TB protection that these enhanced immune responses might produce, as long-term follow-up has not been conducted in any of the above studies, but on balance they suggest that BCG vaccination efficacy may be improved if immunization is delayed. As an example, if the improved immunological outcomes**

resulting from delaying BCG vaccination to 6 weeks of age resulted in a reduction in the annual infection rate of infants by 10%, then 2860 infants per year would be saved from infection. (Annual infection rate = 0.02, 10% of this = 0.002 x 1430000 (live birth rate[69]) = 2860). This equates to a reduction in annual TB infection of 0.6 infants in the delayed BCG arm of our study.

Risks of delaying BCG vaccination to 6 weeks of age

- *6 weeks of potential exposure to tuberculosis without BCG vaccination*

  The following evidence suggests that the actual risk to study subjects arising from 6 weeks exposure to tuberculosis without BCG vaccination is small:

  1) Perinatal TB (TB developing from birth to 8 weeks of age) is extremely rare, with less than 300 cases worldwide described in the literature.[70] The vast majority of these cases are more appropriately described as congenital TB because the source case is the mother. In our study we will exclude infants born to mothers with known active TB or who have any positive TB screening symptoms, to further reduce this risk. The median age of onset of non-congenital TB in infants is 8 months of age; well after our study infants will have received BCG vaccination.
  2) A recent study using an Entebbe based birth-cohort showed a prevalence of latent- TB infection of 9.7% in children under-5 years old.[71] This gives an annual risk of infection of 1.94%, although previous studies argue that this risk is likely to be less in very young infants.[72] In our 560 study infants this equates to a potential 10.9 children/year infected (560 x 0.0194 annual risk of infection). During the 6 week period where half our study infants will not be BCG vaccinated, the likely number of infants at risk of developing **latent TB infection** is thus **0.63** (10.9 children / year = 1.26 infants in 6 weeks /2 as only half infants will be unvaccinated = 0.63 children).
  3) Published studies describing episodes of potential nosocomial TB transmission to neonates on neonatal intensive care units or maternity wards (from other congenitally infected infants, or from active TB in a healthcare worker), showed only 2 cases of infection out of 2603 exposed, BCG unvaccinated, infants who did not receive post-exposure prophylaxis.[73-76] The remaining infants all had negative TST and no signs of clinical disease when followed-up from 3-6 months.[77] This would suggest that if every unvaccinated infant in our study was exposed to TB, we could expect **0.22 cases of TB disease** to result ((2/2603)x280). However, the infants described in these studies are likely to be much more vulnerable to infection and disease than our study subjects due to prematurity, low birth weight and existing medical conditions required their admission. The duration of exposure to the infective contact is also likely to have been much more prolonged in nosocomially infected infants that are nursed continuously in the same

room as the source case, than the infants in our study would be exposed to in the community. Exclusion of infants born into families with a known case of active TB, or for whom a member of the family has any positive TB screening symptoms, will reduce the risks of similar prolonged exposures in our study. **Thus the actual infection rate per exposure to TB in the delayed vaccination group is likely to be negligible.**

4) At least seven previous studies have been conducted in areas of high TB prevalence that randomised infants to delayed BCG vaccination past 6 weeks of age.[4-7, 66, 67] None of these studies showed an increase in TB incidence in the delayed vaccination group (cumulative n for delayed BCG vaccination = 849), and the earliest case of TB in any of these studies was at 3 months of age, well after the time our subjects would receive BCG vaccination.

Although we believe the risks of acquiring TB due to a 6-week delay in vaccination are negligible, we have a number of measures in place to guard against any possible detriment. All infants will be closely followed up for signs and symptoms of TB infection by weekly telephone interviews and regular clinical reviews. Furthermore, any child with delayed BCG vaccination will have this flagged up on their vaccination cards so that when seen by healthcare professionals after the study they are alerted to the delay. Contact details for the study team will be included next to the alert, with a request to be notified if the subject is treated for TB after exiting the study. Although not feasible during the PhD period, we are actively seeking funds to include a longer-term follow-up point in this study, e.g. at 1 year, which could involve assessment for latent TB and treatment of any cases found.

- *If the null hypothesis is incorrect and BCG vaccination does produce a difference in the innate immune response to heterologous pathogens, then infants receiving BCG vaccination at 6 weeks of age might have a 6-week period of increased risk from all-cause infections.*

We are proposing to conduct this study because there is equipoise in the literature as to whether the non-specific effects of BCG vaccination exist, and no currently proposed biological mechanism. The main evidence suggesting a beneficial effect on all-cause mortality comes from Guinea Bissau (one randomised controlled trial[78] (delayed BCG n= 1161), multiple observational studies[29-35]). All other published randomised controlled trials delaying BCG vaccination past 6 weeks of age in high mortality areas have reported **no difference** in rates of mortality, serious illness or TB (delayed BCG subjects combined n=849). Thus it is just as likely that delaying BCG vaccination from birth has no effect on all-cause mortality, or even the reverse, that delaying BCG vaccination past the neonatal period improves all-cause mortality due to a previously unrecognised negative impact of BCG.

If the alternative hypothesis is correct, that neonatal BCG vaccination enhances the innate immune

response to heterologous pathogens, this beneficial effect would not be present in the 6 weeks prior to BCG vaccination for half the study group. If this leads to clinical findings similar to those found in the Guinea-Bissau data, we would predict that this loss of beneficial effect would result in a 45% increased all-cause mortality rate in the infants vaccinated at 6 weeks of age compared to those BCG vaccinated at birth. However, we feel it is extremely unlikely that the clinical impact of BCG vaccination at birth in Uganda would be as pronounced as in Guinea Bissau due to differences in the study populations. If BCG vaccination does enhance the innate immune response to heterologous pathogens then we would predict that infants with the highest exposure and susceptibility to infectious disease, and with the poorest access to treatment when unwell, would derive the greatest benefit from vaccination at birth. There are multiple reasons why these factors are more likely for the infants recruited to the Guinea Bissau study. Firstly, the baseline health status of infants in Guinea Bissau is significantly poorer than in Uganda with neonatal mortality rates nearly twice as high (NMR 44/1000[69] compared to 28/1000[69]). A combination of lower GDP, poorer living conditions and more limited access to health care are likely to contribute strongly to these differences. Secondly, the infants recruited into the Guinea Bissau study were all low birth weight/premature. This group is significantly more susceptible to serious infectious disease and death than the term, normal birth weight infants that we would recruit in this study. Lastly, the Guinea Bissau study had no enhanced clinical follow-up or improved access to health care facilities for its study participants. Information regarding mortality was collected during home visits at 3 days and 2, 6 and 12 months, with no contact in between. Unwell study children therefore utilised normal local health care facilities, which in Guinea Bissau are often of poor quality or difficult to access. In our study we will be conducting intensive and active follow-up of all participants with weekly telephone reviews to check the well-being of the child, review at home if unable to be contacted at weekly telephone review and 4 routine clinic visits before the age of 10 weeks. Transport costs will be reimbursed (10,000 Ugandan Shillings per visit) to reduce barriers to presentation. Subjects will have open access to free assessment by paediatric doctors and treatment at the research clinic throughout the study period. Lastly, the safety monitoring committee will conduct regular comparison of illness outcomes between the two groups, and the study stopped early if one group appears to be significantly more at risk of illness or death than the other. **Thus, with these contingencies in place we strongly believe that we will be able to negate any increased mortality risk from invasive infectious disease that might potentially arise from altered BCG vaccination timing if the null hypothesis is incorrect.**

<u>Benefits of participation in the study for all study participants</u>

- All participants will have regular medical follow-up with four routine clinical reviews by a paediatrician in the first 10 weeks of life. The health of study subjects is therefore likely to be improved due to early recognition and treatment of congenital conditions and serious illnesses.
- All participants will have rapid access to extra medical review and treatment for unwell study subjects.
- All participants will receive primary vaccinations at the correct time. The most recent study looking at vaccination rates in Uganda showed that **56%** of infants have not received their first

set of primary immunisations (diphtheria/tetanus/pertussis/hepatitis B/Haemophilus influenza/polio) by 12 weeks of age, with 26% still not having received it by 1 year of age. This produces a substantial risk for those children of contracting these preventable illnesses that are extremely dangerous in infants. Thus, by ensuring all subjects in our study receive primary immunisations at the correct time we would estimate that we would provide considerable benefits by reducing the risk of these infectious diseases in approximately **314 infants**. These benefits alone are likely to outweigh the extremely low increased risk of TB infection in the delayed vaccination group.

- All costs of transport to attend the clinic and receive vaccinations will be reimbursed. This is likely to improve healthcare utilisation.
- All mothers will receive education about the importance of vaccinations and other basic infant and child health messages.

Potential benefits of the research for child health in Uganda and globally

- If BCG vaccination does provide heterologous protection against non-mycobacterial invasive infectious disease, ensuring that every child is immunised with BCG vaccination as soon as possible after birth could save many lives. In Uganda, for example, although it is recommended that BCG should be received at birth, a recent survey showed that approximately 50% of infants had not received it by 12 weeks of age.[61] The only currently published estimate of the heterologous protection provided by BCG vaccination at birth compared to delayed is a 45% reduction in mortality rate in the first 4 weeks of life (Guinea-Bissau data)[78]. Thus, if we assume that infants in Uganda would have the same mortality reduction, then ensuring that the 50% of infants not currently vaccinated by 12 weeks are vaccinated at birth would prevent **22,800 neonatal deaths per year in Uganda** alone. (Current neonatal mortality rate in Uganda = 28/1000. If a 45% higher mortality rate in unvaccinated neonates is assumed then the differential neonatal mortality rates would be 36/1000 for unvaccinated infants and 20/1000 for vaccinated infants. Thus, ensuring all unvaccinated infants are vaccinated would save 16/1000 deaths per year. With an annual birth rate of 1.43 million, this gives a saving of 22880 neonatal deaths per year). A study comparing 45 low and middle income countries showed that on average 50% of children will not have received BCG vaccination by 4 weeks of age. BCG vaccination is also being phased out in many countries with low TB incidence. As the average global neonatal mortality rate is 36/1000, applying the same logic as before and assuming 50% of children globally will not have received BCG vaccination by 4 weeks of age, this would give a differential global neonatal mortality rate of 26/1000 for BCG vaccinated and 46/1000 for BCG unvaccinated infants. Thus, ensuring all 141 million infants born globally each year are vaccinated at birth could potentially **save 2.82 million deaths per year**.

- This study aims to improve our knowledge of the developing infant immune system up to 10 weeks of age. The current understanding of the developing infant immune system is limited. Increasing our understanding may have broad reaching implications for global child health which are currently difficult to quantify.

- This study aims to improve our understanding of the potential non-specific effects of vaccinations. If our hypothesis proves correct it would suggest the need for all vaccinations to be evaluated on the basis of impacts on all-cause mortality/morbidity rather than just disease-specific outcomes. Again this has the potential to have substantial, and as yet unquantifiable, impacts on the global health of children.

Thus, in summary, we believe that delaying BCG vaccination in this study poses negligible risks of increased TB infection. Although if the null-hypothesis is not correct it could pose a risk from non-TB infectious disease in the delayed vaccination group, we believe this risk would be slight and negated by our active and intensive clinical follow-up. If BCG vaccination at birth does protect against heterologous invasive infectious disease it would have profound impacts on global child health, which we believe outweigh these small risks. A summary of the above evidence is shown in Table 1.

Table 1. Summary of risk-benefits associated with a randomised controlled trial comparing BCG vaccination at birth with BCG vaccination at 6 weeks of age in Ugandan infants

	**Benefits**			**Risks**			**How we will negate any excess risk**
	Common	BCG at birth group	Delayed BCG group	Common	BCG at birth group	Delayed BCG group	
Risk/benefits that may exist **independent** of whether the null hypothesis is correct	✓ Regular paediatric medical review in the first 10 weeks of life providing early recognition and treatment of common and serious neonatal conditions<br>✓ Rapid access to review and treatment if subjects become unwell during the study<br>✓ Timely routine vaccinations during the study period. This is not currently achieved by more than **50%** of Ugandan infants and leaves them at risk of serious illness and death. By ensuring all study infants are vaccinated at the correct time we are likely to reduce the duration of exposure and risk of contracting diphtheria, tetanus, pertussis, hepatitis B, haemophilus influenza and polio in approximately **314 infants**<br>✓ Parental basic health education campaign	✓ 6 weeks of potential protection against TB infection compared to the delayed BCG group.	✓ Reduced BCG induced complications compared to the BCG at birth group. Our best estimate of this reduction is:<br><br>**1 fewer BCG abscesses**<br><br>**0.1 fewer cases of supurative lymphadenitis**<br><br>**0.03 fewer cases of BCG osteomyelitis**<br><br>✓ Potentially increased long-term protection against TB compared to the BCG at birth group. Even a small increase in protection, e.g. 10%, would **prevent 0.6 TB infections** in study infants per year	x Two, 2ml venous blood samples.	x Increased risk of BCG induced complications compared to the delayed BCG vaccination group.<br><br>x Potentially increased long-term risk of TB infection and disease compared to the delayed BCG group	x 6 weeks of potential TB exposure without some protection from BCG. Our best estimate is that at most:<br><br>**<0.63** study infants would be **at risk of latent TB** and<br><br>**<0.22** infants **at risk of TB disease.**<br><br>Due to lower exposure risks and demographic differences in our study these risks are actually likely to be **negligible**	✓ Discomfort and distress of venous blood sampling will be reduced by using only highly trained phlebotomists and by utilising comfort/distraction techniques<br><br>• Any excess risk of tuberculosis or BCG side-effects will be reduced by:<br><br>✓ Exclusion of infants born to mothers with known active TB or with positive features on TB symptom screen<br>✓ Exclusion of infants born into families with a member with known active TB or positive features on TB symptom screen<br>✓ Weekly telephone reviews of all patients<br>✓ Four clinical reviews prior to 10 weeks of age<br>✓ Open and rapid access to paediatric review, assessment and treatment if unwell.<br>✓ High index of suspicion for tuberculosis related diseases<br>✓ Un-blinding of patients where tuberculosis or BCG related disease are suspected<br>✓ Conservative

							stopping rules for safety if excess TB cases are found in either group during regular interim analysis
**Potential** risks/benefits that may exist **if our null hypothesis is incorrect**	✓ Provide evidence to advocate for BCG vaccination of all infants at birth which could prevent: **22,800 Ugandan** and **2.8 million global neonatal deaths/year**<br>✓ Increase knowledge of the developing infant immune system. This may have wide-ranging future benefits and applications.<br>✓ Increase knowledge regarding the non-specific effects of vaccination, suggesting the necessity of evaluating all current and future vaccinations in terms of all-cause mortality, and not just disease-specific mortality.	✓ If the null hypothesis is incorrect then the group receiving BCG at birth would have a reduced risk of all-cause infectious disease/ mortality compared to the delayed vaccination group, prior to the 6-week vaccination group.				x If the null hypothesis is incorrect the delayed BCG group could have an increased risk of all-cause infectious disease/mortality in the 6 weeks prior to vaccination. This risk is likely to be **smaller** than that currently present for the 50% of Ugandan neonates that do not currently receive BCG vaccine by 12 weeks of age due to our active follow-up.	• Active and intensive clinical follow-up of all children:<br><br>✓ Weekly telephone reviews<br>✓ Four clinical reviews prior to 10 weeks of age<br>✓ Open and rapid access to paediatric review, assessment and treatment if unwell<br>✓ Conservative stopping rules for safety if excess clinical illness episodes are found in either group during regular interim analysis

Evaluation of alternative study designs

During the development of this proposal, several other study designs were considered. However, there are several reasons why we have concluded that a randomized controlled trial design, as described, is imperative for this work.

a) *Observational studies, particularly involving vaccinations, are highly susceptible to selection bias.*

It is argued in the literature that children who have delayed vaccinations are likely to be fundamentally different to those that receive their vaccinations at the recommended times, confounding the interpretation of the results of this study.[79] Such differences include:

- Lower socioeconomic status[80]
- Lower educational status of parents[80]
- Poorer nutritional status[30]
- Parents less pro-active about the health of their infants, or less able to access healthcare facilities[80]
- More likely to be born at home with unskilled birth attendants rather than in hospital[81]

It is also recognized that observational studies comparing children vaccinated at the recommended time with those where vaccination has been delayed are subject to frailty bias, as vaccinations tend to be delayed in acutely unwell or chronically sick children and those of low birth weight.[79]

All of the above factors are likely to impact on the immunological status of the child and increase their risk of clinical illness episodes, due to factors such as increased exposure to infectious pathogens from less hygienic living conditions and overcrowding, as well as altered healthcare seeking behavior/access negatively influencing illness episode outcomes.

Thus, the likely predominance of healthier neonates in the vaccinated group may bias both the primary immunological outcome measures and our clinical illness data toward making BCG vaccination at birth appear more beneficial against all-cause morbidity and mortality than it really is.

Although it is possible to document and adjust for the recognized variables associated with delayed vaccine receipt, it is strongly argued in the literature that it is impossible to anticipate all confounding variables separating the two groups. Multiple observational studies have

been conducted, including case-control studies and prospective/retrospective cohort analyses, investigating the potential non-specific effects of a range of vaccinations (reviewed in [9]) These have had minimal acceptance by the research community and public health policy makers for the above reasons. In a review of the potential non-specific effects of vaccinations, the Global Advisory Committee on Vaccines has stated that 'conclusive evidence for or against non-specific effects of vaccines on mortality is unlikely to be obtained from observational studies'.[82]

Thus, we believe that the information gained from an observational study would be insufficient to accept or refute our hypothesis, would not significantly add to what is already known in this field, and would have little impact on public healthcare policy in Uganda and worldwide. We therefore argue that it may be less ethically justifiable to subject infants to blood draws and commit research funds and resources to an observational study, when we believe a randomized controlled trial delaying BCG vaccination may be safely carried out.[64]

b) *Inability to collect baseline cord blood, early neonatal blood samples and early clinical outcome data if we passively recruit infants attending vaccination clinics who have missed vaccination at birth.*

The evidence from Guinea-Bissau suggests that the major benefit from BCG vaccination at birth is derived within the first week of life.[28] It would be impossible in an observational study that recruits non-BCG vaccinated infants that are attending community vaccination clinics (with attendance normally at 6 weeks of age for their first primary immunisations) to compare immunological and clinical data between the two groups at these vital early time-points.

Other ethical considerations

BCG immunisation of premature infants

As described previously, no gestational age limit has been set for this study with children eligible for participation provided they are mature enough to be discharged directly from the maternity ward with no requirement for inpatient support. This will result in some infants <37 weeks gestation receiving BCG immunisation on the first day of life. Research has shown that BCG immunisation has no increased adverse consequences when given to premature infants and specific-mycobacterial responses are as efficacious as in term infants.[57]

Blood sampling

Blood sampling from young infants has the potential to cause discomfort to the infant and distress to the mother. This will be minimised by phlebotomy being carried out by well-trained and experienced research staff. Distraction techniques and comfort feeding will be utilised to minimise distress and a maximum of two attempts per time-point will be allowed. Venous blood sampling has been chosen due to the reduced risk of discomfort and local infection in comparison to heel-prick sampling.[83] We have considered the use of topical anaesthetic cream during blood sampling, but have decided not to offer this due to lack of evidence of benefit in young infants, risk of local and systemic side-effects and its lack of routine use in clinical practice in children under the age of 1 year.[84] Previous studies obtaining blood samples from children in the area have not used topical anaesthetic creams.

A maximum of 2ml of blood will be drawn at each sampling point. This is well below recommended sampling volumes for research[85] and is highly unlikely to be detrimental to the infant. Any abnormal indices discovered in blood samples will be followed up with clinical review and treatment of the child as appropriate. The justification for taking this volume of blood is as follows:

Cytokine sub-study:

200µl of whole blood per stimulant is required to produce approximately 300µl of supernatant. This is sufficient to conduct ELISA studies for 5 cytokines (IL-1β, IL-6, IL-10, TNF-α, IFNγ) in singlecate with 50µl of excess in case any samples need to be re-analysed. As there are 6 stimulations conducted per sample (S. aureus, S. pneumoniae, E. coli, C. albicans, FEC, positive and negative controls) a minimum of 1.2ml (200µl x 6) whole blood is required to conduct this work. Up to 2ml of blood will be collected from each infant to provide a margin for error. Any excess will be stored for future work, subject to parental consent and further ethical approval.

Iron sub-study:

Full Blood Count and red cell parameters: These are conducted by automated machine and require a minimum of 250µl of whole EDTA blood. 400µl will be collected to provide a margin of safety.

Iron studies: The Cobas Integra Machine requires a minimum of 500µl of plasma to analyse the iron status parameters. As plasma comprises approximately 50% of blood, 1ml of whole

lithium-heparinised blood will be required as a minimum for this analysis. 1.2ml will be collected to provide a margin for error.
Hepcidin analysis: A minimum of 150µl of plasma is required to conduct Hepcidin analysis in triplicate. A further 50µl will be collected to allow samples to be re-run if any are out of range of the initial standard curve. Thus a total of 200µl plasma is required, which is equivalent to 400µl of whole blood

Thus 400µl EDTA and 1.6ml (1.2ml + 400µl) of Li-Heparinised blood will be required = 2ml total venous blood.

Epigenetic sub-study:
2 million viable Peripheral Blood Mononuclear Cells are the absolute minimum required to conduct Chromatin Immunoprecipitation effectively, using current techniques. 2ml of venous blood is the minimum required to achieve this number.

The possibility of parents relaxing their attitudes to vaccination after their child has exited the study

This study provides multiple contact points between mothers and healthcare professionals within the first 10 weeks of life. We plan to use each of these visits as an opportunity to provide education about the importance of vaccinations, as well as other basic infant health care messages. This education will be provided both verbally during consultations with nurses and doctors, and in the form of posters in the research clinic. When a child exits the study at 10 weeks mothers will be reminded verbally of the importance of receiving the remainder of their vaccinations. The dates these vaccinations are due will be documented on their vaccination card and details of their nearest community vaccination clinic provided. We envisage that one of the benefits of this study will be a positive impact on infant vaccination rates amongst participants, not a relaxation of parental attitudes to vaccination

Data security and confidentiality

The main risk to confidentiality is discovery of subject's participation in the study by a third party. Data collection has been designed to minimise this risk. Each study subject will be identified through a unique identifier number. Documentation under this number only will be used for the electronic database, electronic capture of clinical information and paper laboratory forms. Consent forms will contain mother and participants names and addresses but not the unique identifier. There will be two places where the participants name and unique identifier will be stored together:

1) Linking File: the master file linking participants name with their unique identifier and intervention allocation. This will be managed by the data

manager and stored securely on the host unit server. No other data collected from the study will be stored in this file.

2) Participant Personal Study Plan: Each participant will have a paper file containing their name, date of birth, contact details, consent form and allocation for intervention/blood sampling, along with the unique identifier number. No outcome data from the study will be stored in this file. The linking of this data is imperative to ensure that each child seen at the clinic is identified correctly for blood tests and electronic data capture, and receives the correct intervention. These files will be stored securely at the clinic. Only the head research nurse will have access to the key. No access will be granted to any investigator documenting outcome measures (clinical or laboratory) to maintain blinding.

This study has already been peer reviewed during the process of gaining Wellcome Trust Funding. Specific ethical approvals will be sought from the Uganda Virus Research Institute Science and Ethics Committee, the Uganda National Council for Science and Technology, and the London School of Hygiene and Tropical Medicine Research Ethics Committee.

Clinical trial oversight and monitoring

This clinical trial will be conducted according to Good Clinical Practice standards, with oversight and monitoring co-ordinated by the London School of Hygiene and Tropical Medicine. This will include internal audit by the Clinical Trials Quality Assurance Manager and may include external audits by a third party. A Trial Monitoring Group (TMG), consisting of the PI, primary supervisor, data manger, statistician and head research nurse, will co-ordinate the day-to-day management of the clinical trial and conduct quality assurance procedures. A Trial Steering Committee (TSC) with members independent of the trial investigators will also be established. An independent Data Safety and Monitoring Board (DSMB) will be set up, with access to the un-blinded data set. The Data Safety Monitoring board will look at a number of clinical outcome measures, documented in real time during the study, to assess whether the study needs to be stopped early for safety. These will include physician diagnosed invasive infectious disease, documented febrile episodes, documented respiratory distress, dehydration secondary to diarrhoea and death. A composite measure of this will be compared between the two groups to assess the need to halt the trial early for safety:

Hospital admissions for reasons other than injury **+**

Clinic presentation with fever >38 degrees not requiring admission **+**

Clinic presentation with respiratory distress not requiring admission **+**

Clinic presentation with diarrhoea not requiring admission **+**

Death

This measure encompasses all diseases symptoms that were reported as significantly different between intervention groups in the Guinea-Bissau randomized controlled trial of BCG vaccination at birth compared to 6 weeks of age and we believe is a conservative measure to capture all likely significant infectious illness episodes. As parents are not blinded to intervention allocation we have decided not to include the outcome measure of parental recall of illness episodes due to the strong possibility of recall bias. The Data Safety Monitoring Board will define the exact number of interim analyses conducted, and this will inform the statistical degree of difference between intervention groups required at each time point to suggest the need to stop the trial early for safety.

Rates of suspected tuberculosis will also be compared between the two groups at each interim analysis and the study halted if they are significantly different between the two groups.

We will notify the SEC of the names of all board/committee members, as well as stopping criteria, prior to study commencement.

Safety reporting for this trial will follow London School of Hygiene and Tropical Medicine procedures which are in accordance with good clinical practice. These procedures are found in Appendix 1 of the protocol.

6. Data management

Data entry

Description of the data

This is a randomised controlled trial with datasets generated from clinical questionnaires and laboratory assays. A combination of direct electronic capture and paper forms will be used, linked by a unique participant identifier. Microsoft Access will be utilised to produce the study database. Data will be exported from Microsoft Access to Stata for statistical analysis.

Quality Assurance

A detailed data dictionary with range checks will be used to reduce data entry errors. Quality control checks will be run by the data clerk, on a weekly basis, who will highlight any queries to the principal investigator.

Data storage strategy

During the study the master database will be maintained on the MRC Unit server. This server is backed up to a secure location using a mirrored server. In addition, the principal investigator may maintain a password-protected and encrypted version of the database on her computer/external hard-drive.

Consent and laboratory paper forms generated during the study will be stored securely at the host unit. Participant personal study plans will be stored securely at the research clinic during the study duration, and will be transferred to secure storage at the host unit on study completion.

A secure copy of the data from this study will be preserved in a Database Repository at the administering institution (LSHTM) and host Unit. This will be maintained for at least 10 years following study completion.

Responsibilities

Sarah Prentice, with support from co-supervisors and study statistician will be responsible for the overall data management of this study.

Analysis plan

Group characteristics will be compared using Pearson's Chi-squared test for categorical variables and the t-test for continuous variables. Potential confounders will be adjusted for using multiple linear regression analysis. Cross-sectional comparisons between intervention groups at each time-point will be carried-out using the t-test for significant difference of means, with logarithmic transformation of non-normally distributed data. Mann-Whitney two-tailed test will be used for persistently skewed data. Paired/longitudinal analysis of within infant changes in parameters over time will be conducted using the paired student t-test or Wilcoxon matched-pairs test. Incidence rate of invasive infectious disease in the first 10 weeks of life will be compared by Poisson regression with a random effects model to allow for within-child clustering. Statistical significance will be assessed at the two-sided 0.05 level

but interpretation of results will not be solely reliant on P-values. A correction for multiple testing will be applied.

7. Potential limitations; anticipated problems

Potential logistical problems

Recruitment numbers

This project aims to recruit 560 infants within 1 year. However, another study recruiting neonates born in Entebbe Grade B Hospital is due to overlap with this study, potentially slowing recruitment rate. To ensure both studies get the numbers they wish, we may also recruit from Kisubi Hospital, and also alternate recruitment to each study weekly. Birth rates in Entebbe Grade B Hospital are high (300-400 per month) and previous studies have achieved recruitment rates of 3 per day, suggesting that recruitment of 560 infants is feasible within the given timeframe.

Loss to follow-up

Ensuring minimal loss to follow-up is a priority, particularly in the delayed BCG group. All mothers and infants will be driven home from hospital to ensure correct documentation of address and contact details. A field worker will be employed solely to contact non-attenders, by telephone and/or in person, to request clinic attendance. Transport costs for routine clinic attendance will be reimbursed (10,000 Ugandan Shillings per visit) to reduce barriers to presentation. Any BCG naïve infant who drops-out of the study will be offered BCG immunisation immediately. Mothers will not be blinded to vaccine administration status to avoid confusion if un-traceable subjects lost to follow-up are seen in community immunisation clinics.

Blood volume

Blood collection from neonates is challenging and may produce smaller samples than anticipated. This will be minimised by utilisation of clinic staff with training and experience in paediatric phlebotomy. Sample numbers for each sub-study also have excess built in to account for insufficient sample volumes and attrition. This is particularly pertinent for the epigenetic studies, which require a full 2ml of blood for analysis. Use of pooled samples for epigenetic work may be considered if collection of adequate blood volumes is problematic.

BCG vaccine supply

Differences in BCG strain and batch have been shown to alter specific and non-specific immune response to immunisation,[3] potentially confounding results. BCG-Danish will be imported from the Statens Serum Institute specifically for the study, ensuring strain homogeneity. As far as possible the same batch of BCG will be utilised, particularly within each sub-study. Batch alteration, due to supply limitations, will be documented and adjusted for in final analysis.

Potential study design limitations

Blinding

The lack of double-blinding in this study may potentially bias clinical results, as mothers may associate immunisation (or lack of) with illness and be more likely to recall illness episodes or present with concerns. We feel, however, that blinding mothers would be impossible (due to the visible scar reaction associated with BCG) and unethical, leading to potential confusion if infants are lost to follow-up. Blinding of clinicians to immunisation status during routine and illness episode assessment will be achieved by placing a plaster over the left deltoid of all participants. Thus, objective clinical assessment should provide an unbiased outcome measure.

Confounding influence of other immunisations

Other vaccines in the EPI schedule may have impacts on heterologous innate immune responses, minimising observed differences between the two intervention groups._ENREF_30[30] This study has been designed with this specifically in mind. The first two blood sampling time-points occur when only OPV has been given in addition to the BCG intervention. The evidence for a heterologous effect of OPV immunisation remains inconclusive and it's oral route of administration may limit its impact on systemic heterologous immunity.[86, 87] Importantly, the Guinea-Bissau study that showed large mortality benefits with early BCG immunisation, also immunised all infants with OPV immunisation at birth.[28] This suggests that OPV immunisation will have minimal influence on the heterologous immune response investigated in this study. The later two blood sampling time-points occur after receipt of the first dose of primary immunisations, allowing interrogation of the impacts of other immunisations on innate immune responses to heterologous infections.

Confounding influence of illnesses around blood sampling time point

Illness around the time of blood sampling is likely to alter innate immune responses, potentially confounding results. If a child is deemed acutely unwell at blood sampling time, sampling will either be delayed a short time until well (and this delay documented) or the child will be excluded from this sampling time point but remain in the study for future time-points. If the child is unwell at the time immunisation is due, Ugandan immunisation protocols will be followed (usually immunisation is delayed until the child is well). This delay will be documented and any post-immunisation blood samples will be timed from immunisation receipt.

8. Significance of the proposed work

Significance for basic science (immunology and vaccinology)

The cytokine sub-study will contribute knowledge to our understanding of innate immune responses in neonates, innate immune responses to BCG vaccine and heterologous immune responses following BCG immunisation. The iron sub-study will contribute knowledge of iron homeostasis in neonates, the iron-inflammatory response in neonates and the iron-inflammatory response to BCG immunisation/heterologous stimulants. Data on these parameters are currently extremely limited. By comparing the above immune responses at birth and 6 weeks, information about critical periods in the development of the neonatal innate immune system will also be obtained.

The epigenetic modification experiments will investigate the paradigm-shifting possibility of 'training' of the innate immune system by stimuli such as immunisations. Limited evidence for such an effect has been found in adult studies, but has never been investigated in children. Such an effect could have profound implications for vaccine design, suggesting vaccines should be evaluated for their overall effect on all-cause morbidity and mortality, rather than just disease specific efficacy.

Clinical and public health significance

The potential for BCG immunisation to protect against a range of pathogens, particularly in the neonatal period, is extremely exciting. Sepsis accounts for 1/3rd of

all neonatal deaths and a low-cost, safe, widely available intervention to reduce this would have a huge impact on under-5 mortality rates. Although BCG immunisation is in routine use throughout the world, immunisation is often delayed or missed, with some countries also phasing-out its use. If our hypothesis is correct more than 2.8 million neonatal deaths could be prevented each year by ensuring all children receive BCG immunisation at birth (see risk:benefit analysis above). This identification of unrecognised benefits from an existing intervention would thus have enormous impacts on global child health.

9. Plans for dissemination

In Uganda, the Uganda Virus Research Institute is an organ of the Ministry of Health and has ready access to policy makers to discuss proposed work and to present results. In addition, the Co-infection Studies Programme (CiSP), led by Professor Elliott, has a long track record of tuberculosis-related research in Uganda and established links with the National Tuberculosis and Leprosy Control Programme. The proposed work will be discussed with the NTLP leadership prior to its commencement, and regular reports will be made on the progress of the work and its results.

CiSP also has an established record of engagement with the research community and participants, including the involvement of local volunteer field workers, and community and participant meetings. These avenues will be used to foster community support for the proposed work, and to explain the proposed work and its results to the participating community. In addition, the MRC Unit in Uganda has appointed a Communications and Public Engagement officer with effect from the 1st September 2012. This individual will be engaged to help in the dissemination of the results in Uganda and beyond.

The London School of Hygiene and Tropical Medicine has a media relations department, and any important or interesting development arising from this study will be communicated to them. This department within the School has been set up to engage the wider audience by notifying both print and electronic media, as well as developing podcasts that can be made available from the School's website. This will be of particular relevance if our study shows that maternal infection status affects the infant response to BCG immunisation. This would have large public health implications, and the School's media relations department are ideally trained to disseminate such information to the general public, and of course to handle any media requests for interviews.

The research community will be made aware of our findings by dissemination through traditional publishing routes and presentations at international and local meetings. In addition, all investigators on this proposal are members of the LSHTM TB Centre (http://tb.lshtm.ac.uk). Published results can be highlighted on this website, and any interested person can read about projects that that individual is involved with. High quality publications and interesting data that arise from this study may also be highlighted on MRC UK and MRC/UVRI Uganda Research Unit on AIDS websites.

10.Time frame

This project is expected to run from January 2014 to October 2016

11. Budget

This project will be fully funded by the Principal Investigators Wellcome Trust Clinical Fellowship Award. This award allows £130,000 research expenses and pays the Principal Investigator's salary and overseas living allowance. The award will be administered through the London School of Hygiene and Tropical Medicine.

12. Appendices

Appendix 1 – Study Forms (hard copy examples – the data will be electronically captured)

a) Recruitment and Eligibility Form
b) Maternal Demographics and Birth Details Form
c) Personal Participant Plan
d) Routine Clinical Review Form
e) Illness Episode Form

Appendix 2 – Safety Reporting Procedures

12. References

1. Trunz BB, Fine P, Dye C. Effect of BCG vaccination on childhood tuberculous meningitis and miliary tuberculosis worldwide: a meta-analysis and assessment of cost-effectiveness. *Lancet* 2006; **367**(9517): 1173-80.
2. Fine PE. Variation in protection by BCG: implications of and for heterologous immunity. *Lancet* 1995; **346**(8986): 1339-45.
3. Anderson EJ, Webb EL, Mawa PA, et al. The influence of BCG vaccine strain on mycobacteria-specific and non-specific immune responses in a prospective cohort of infants in Uganda. *Vaccine* 2012; **30**(12): 2083-9.
4. Kagina BM, Abel B, Bowmaker M, et al. Delaying BCG vaccination from birth to 10 weeks of age may result in an enhanced memory CD4 T cell response. *Vaccine* 2009; **27**(40): 5488-95.

5. Suciliene E, Ronne T, Plesner AM, et al. Infant BCG vaccination study in Lithuania. *The international journal of tuberculosis and lung disease : the official journal of the International Union against Tuberculosis and Lung Disease* 1999; **3**(11): 956-61.
6. Ildirim I, Sapan N, Cavusoglu B. Comparison of BCG vaccination at birth and at third month of life. *Archives of disease in childhood* 1992; **67**(1): 80-2.
7. Hussey GD, Watkins ML, Goddard EA, et al. Neonatal mycobacterial specific cytotoxic T-lymphocyte and cytokine profiles in response to distinct BCG vaccination strategies. *Immunology* 2002; **105**(3): 314-24.
8. Kaufmann SH, Hussey G, Lambert PH. New vaccines for tuberculosis. *Lancet* 2010; **375**(9731): 2110-9.
9. Shann F. The nonspecific effects of vaccines and the expanded program on immunization. *The Journal of infectious diseases* 2011; **204**(2): 182-4.
10. Aronson JD. BCG vaccination among American Indians. *American review of tuberculosis* 1948; **57**(1): 96-9.
11. Aronson JD. Protective vaccination against tuberculosis, with special reference to BCG vaccine. *Minnesota medicine* 1948; **31**(12): 1336.
12. Levine MI, Sackett MF. Results of BCG immunization in New York City. *American review of tuberculosis* 1946; **53**: 517-32.
13. Shann F. The non-specific effects of vaccines. *Archives of disease in childhood* 2010; **95**(9): 662-7.
14. Rosenthal SR, Loewinsohne, Graham ML, Liveright D, Thorne G, Johnson V. BCG vaccination against tuberculosis in Chicago. A twenty-year study statistically analyzed. *Pediatrics* 1961; **28**: 622-41.
15. Gibson DH, Baskerville A, Ashworth LA, Fitzgeorge RB. Non-specific protection against pulmonary Legionella pneumophila infection in guinea-pigs immunized and challenged with mycobacteria. *British journal of experimental pathology* 1985; **66**(3): 333-44.
16. Kato G, Kondo H, Aoki T, Hirono I. Mycobacterium bovis BCG vaccine induces non-specific immune responses in Japanese flounder against Nocardia seriolae. *Fish & shellfish immunology* 2012; **33**(2): 243-50.
17. Ruitenberg EJ, Van Noorle Jansen LM, Kruizinga W, Steerenberg PA. Effect of pretreatment with Bacillus Calmette-Guerin on the course of a Listeria monocytogenes infection in normal and congenitally athymic (nude) mice. *British journal of experimental pathology* 1976; **57**(3): 310-5.
18. Ujiie A, Watanabe M, Ushiba D. [Non-specific resistance to the infection with Salmonella enteritidis induced in mice vaccinated with BCG. (II)]. *Nihon saikingaku zasshi Japanese journal of bacteriology* 1966; **21**(12): 701-6.
19. Yang J, Kawamura I, Mitsuyama M. Involvement of inflammatory cytokines and nitric oxide in the expression of non-specific resistance to Listeria monocytogenes in mice induced by viable but not killed Mycobacterium bovis BCG. *Microbial pathogenesis* 1997; **22**(2): 79-88.
20. Mudd S, Varnell ED, Engelstein J. The effect of nonspecific immune stimulation on the recurrence rate of herpetic keratitis in rabbits. *Investigative ophthalmology* 1975; **14**(6): 469-71.
21. Spencer JC, Ganguly R, Waldman RH. Nonspecific protection of mice against influenza virus infection by local or systemic immunization with Bacille Calmette-Guerin. *The Journal of infectious diseases* 1977; **136**(2): 171-5.
22. Werner GT. The effect of BCG-vaccination on vaccinia virus infections in mice. *Experientia* 1979; **35**(11): 1514-5.

23. Maiti PK, Kumar A, Kumar R, Mohapatra LN. Role of antibodies and effect of BCG vaccination in experimental candidiasis in mice. *Mycopathologia* 1985; **91**(2): 79-85.
24. Kleinnijenhuis J, Quintin J, Preijers F, et al. Bacille Calmette-Guerin induces NOD2-dependent nonspecific protection from reinfection via epigenetic reprogramming of monocytes. *Proceedings of the National Academy of Sciences of the United States of America* 2012; **109**(43): 17537-42.
25. Ghadirian E, Kongshavn PA. Protection of mice against intestinal amoebiasis with BCG, Corynebacterium parvum and Listeria monocytogenes. *Parasite immunology* 1986; **8**(6): 663-7.
26. Langley RJ, Gray JS. Non-specific resistance to Babesia divergens in the Mongolian gerbil (Meriones unguiculatus). *International journal for parasitology* 1989; **19**(3): 265-9.
27. Murray M, Morrison WI. Non-specific induction of increased resistance in mice to Trypanosoma congolense and Trypanosoma brucei by immunostimulants. *Parasitology* 1979; **79**(3): 349-66.
28. Aaby P, Roth A, Ravn H, et al. Randomized trial of BCG vaccination at birth to low-birth-weight children: beneficial nonspecific effects in the neonatal period? *The Journal of infectious diseases* 2011; **204**(2): 245-52.
29. Garly ML, Martins CL, Bale C, et al. BCG scar and positive tuberculin reaction associated with reduced child mortality in West Africa. A non-specific beneficial effect of BCG? *Vaccine* 2003; **21**(21-22): 2782-90.
30. Kristensen I, Aaby P, Jensen H. Routine vaccinations and child survival: follow up study in Guinea-Bissau, West Africa. *Bmj* 2000; **321**(7274): 1435-8.
31. Roth A, Garly ML, Jensen H, Nielsen J, Aaby P. Bacillus Calmette-Guerin vaccination and infant mortality. *Expert review of vaccines* 2006; **5**(2): 277-93.
32. Roth A, Sodemann M, Jensen H, et al. Tuberculin reaction, BCG scar, and lower female mortality. *Epidemiology* 2006; **17**(5): 562-8.
33. Rodrigues A, Fischer TK, Valentiner-Branth P, et al. Community cohort study of rotavirus and other enteropathogens: are routine vaccinations associated with sex-differential incidence rates? *Vaccine* 2006; **24**(22): 4737-46.
34. Stensballe LG, Nante E, Jensen IP, et al. Acute lower respiratory tract infections and respiratory syncytial virus in infants in Guinea-Bissau: a beneficial effect of BCG vaccination for girls community based case-control study. *Vaccine* 2005; **23**(10): 1251-7.
35. Veirum JE, Sodemann M, Biai S, et al. Routine vaccinations associated with divergent effects on female and male mortality at the paediatric ward in Bissau, Guinea-Bissau. *Vaccine* 2005; **23**(9): 1197-204.
36. Bank W. 2011.
37. Marchant A, Goetghebuer T, Ota MO, et al. Newborns develop a Th1-type immune response to Mycobacterium bovis bacillus Calmette-Guerin vaccination. *Journal of immunology* 1999; **163**(4): 2249-55.
38. Madura Larsen J, Benn CS, Fillie Y, van der Kleij D, Aaby P, Yazdanbakhsh M. BCG stimulated dendritic cells induce an interleukin-10 producing T-cell population with no T helper 1 or T helper 2 bias in vitro. *Immunology* 2007; **121**(2): 276-82.
39. Vekemans J, Amedei A, Ota MO, et al. Neonatal bacillus Calmette-Guerin vaccination induces adult-like IFN-gamma production by CD4+ T lymphocytes. *European journal of immunology* 2001; **31**(5): 1531-5.
40. van der Meijden AP, Debruyne FM, Steerenberg PA, de Jong WH. Aspects of non-specific immunotherapy with BCG in superficial bladder cancer: an overview. *Progress in clinical and biological research* 1989; **310**: 11-33.
41. Sun J, Hou J, Li D, et al. Enhancement of HIV-1 DNA vaccine immunogenicity by BCG-PSN, a novel adjuvant. *Vaccine* 2013; **31**(3): 472-9.

42. Andersen A, Roth A, Jensen KJ, et al. The immunological effect of revaccination with Bacille Calmette-Guerin vaccine at 19 months of age. *Vaccine* 2013; **31**(17): 2137-44.
43. Djuardi Y, Sartono E, Wibowo H, Supali T, Yazdanbakhsh M. A longitudinal study of BCG vaccination in early childhood: the development of innate and adaptive immune responses. *PloS one* 2010; **5**(11): e14066.
44. Drakesmith H, Prentice AM. Hepcidin and the iron-infection axis. *Science* 2012; **338**(6108): 768-72.
45. Bullen JJ, Leigh LC, Rogers HJ. The effect of iron compounds on the virulence of Escherichia coli for guinea-pigs. *Immunology* 1968; **15**(4): 581-8.
46. Kochan I. The role of iron in bacterial infections, with special consideration of host-tubercle bacillus interaction. *Current topics in microbiology and immunology* 1973; **60**: 1-30.
47. Debebe Z, Ammosova T, Jerebtsova M, et al. Iron chelators ICL670 and 311 inhibit HIV-1 transcription. *Virology* 2007; **367**(2): 324-33.
48. Breidbach T, Scory S, Krauth-Siegel RL, Steverding D. Growth inhibition of bloodstream forms of Trypanosoma brucei by the iron chelator deferoxamine. *International journal for parasitology* 2002; **32**(4): 473-9.
49. Francisco AF, de Abreu Vieira PM, Arantes JM, et al. Trypanosoma cruzi: effect of benznidazole therapy combined with the iron chelator desferrioxamine in infected mice. *Experimental parasitology* 2008; **120**(4): 314-9.
50. Gwamaka M, Kurtis JD, Sorensen BE, et al. Iron deficiency protects against severe Plasmodium falciparum malaria and death in young children. *Clinical infectious diseases : an official publication of the Infectious Diseases Society of America* 2012; **54**(8): 1137-44.
51. Harvey PW, Bell RG, Nesheim MC. Iron deficiency protects inbred mice against infection with Plasmodium chabaudi. *Infection and immunity* 1985; **50**(3): 932-4.
52. Fiori A, Van Dijck P. Potent synergistic effect of doxycycline with fluconazole against Candida albicans is mediated by interference with iron homeostasis. *Antimicrobial agents and chemotherapy* 2012; **56**(7): 3785-96.
53. Kim J, Cho YJ, Do E, et al. A defect in iron uptake enhances the susceptibility of Cryptococcus neoformans to azole antifungal drugs. *Fungal genetics and biology : FG & B* 2012; **49**(11): 955-66.
54. Kochan I. Mecahnism of tuberculostasis in mammalian serum. I. Role of transferrin in human serum tuberculostasis. *The Journal of infectious diseases* 1969; **119**(1): 11-8.
55. Becroft DM, Dix MR, Farmer K. Intramuscular iron-dextran and susceptibility of neonates to bacterial infections. In vitro studies. *Archives of disease in childhood* 1977; **52**(10): 778-81.
56. Barry DM, Reeve AW. Iron and infection. *British medical journal* 1988; **296**(6638): 1736.
57. D'Angio CT. Immunization of the premature infant. *The Pediatric infectious disease journal* 1999; **18**(9): 824-5.
58. Mugalu J, Nakakeeto MK, Kiguli S, Kaddu-Mulindwa DH. Aetiology, risk factors and immediate outcome of bacteriologically confirmed neonatal septicaemia in Mulago hospital, Uganda. *African health sciences* 2006; **6**(2): 120-6.
59. Paiva Ade A, Rondo PH, Pagliusi RA, Latorre Mdo R, Cardoso MA, Gondim SS. Relationship between the iron status of pregnant women and their newborns. *Revista de saude publica* 2007; **41**(3): 321-7.
60. Elliott AM, Namujju PB, Mawa PA, et al. A randomised controlled trial of the effects of albendazole in pregnancy on maternal responses to mycobacterial antigens and infant responses to Bacille Calmette-Guerin (BCG) immunisation [ISRCTN32849447]. *BMC infectious diseases* 2005; **5**: 115.

61. Clark A, Sanderson C. Timing of children's vaccinations in 45 low-income and middle-income countries: an analysis of survey data. *Lancet* 2009; **373**(9674): 1543-9.
62. Institute SS. BCG-Danish Product Characteristic Leaflet. 2013.
63. Price JF. BCG vaccination. *Archives of disease in childhood* 1982; **57**(7): 485-6.
64. Shann F, Nohynek H, Scott JA, Hesseling A, Flanagan KL, Working Group on Nonspecific Effects of V. Randomized trials to study the nonspecific effects of vaccines in children in low-income countries. *The Pediatric infectious disease journal* 2010; **29**(5): 457-61.
65. BCG vaccination policies. Report of a WHO study group. *World Health Organization technical report series* 1980; **652**: 1-17.
66. Pabst HF, Godel JC, Spady DW, McKechnie J, Grace M. Prospective trial of timing of bacillus Calmette-Guerin vaccination in Canadian Cree infants. *The American review of respiratory disease* 1989; **140**(4): 1007-11.
67. Ota MO, Vekemans J, Schlegel-Haueter SE, et al. Influence of Mycobacterium bovis bacillus Calmette-Guerin on antibody and cytokine responses to human neonatal vaccination. *Journal of immunology* 2002; **168**(2): 919-25.
68. Burl S, Adetifa UJ, Cox M, et al. Delaying bacillus Calmette-Guerin vaccination from birth to 4 1/2 months of age reduces postvaccination Th1 and IL-17 responses but leads to comparable mycobacterial responses at 9 months of age. *Journal of immunology* 2010; **185**(4): 2620-8.
69. https://http://www.cia.gov/library/publications/the-world-factbook/fields/2091.html. Central Intelligence Agency - World Factbook. 2013.
70. Whittaker E, Kampmann B. Perinatal tuberculosis: new challenges in the diagnosis and treatment of tuberculosis in infants and the newborn. *Early human development* 2008; **84**(12): 795-9.
71. Nkurunungi G, Lutangira JE, Lule SA, et al. Determining Mycobacterium tuberculosis infection among BCG-immunised Ugandan children by T-SPOT.TB and tuberculin skin testing. *PloS one* 2012; **7**(10): e47340.
72. Stott H, Patel A, Sutherland I, et al. The risk of tuberculous infection in Uganda, deprived from the findings of national tuberculin surveys 1958 and 1970. *Tubercle* 1973; **54**(1): 1-22.
73. Light IJ, Saidleman M, Sutherland JM. Management of newborns after nursery exposure to tuberculosis. *The American review of respiratory disease* 1974; **109**(4): 415-9.
74. Steiner P, Rao M, Victoria MS, Rudolph N, Buynoski G. Miliary tuberculosis in two infants after nursery exposure: epidemiologic, clinical, and laboratory findings. *The American review of respiratory disease* 1976; **113**(3): 267-71.
75. Burk JR, Bahar D, Wolf FS, Greene J, Bailey WC. Nursery exposure of 528 newborns to a nurse with pulmonary tuberculosis. *Southern medical journal* 1978; **71**(1): 7-10.
76. Myers JP, Perlstein PH, Light IJ, Towbin RB, Dincsoy HP, Dincsoy MY. Tuberculosis in pregnancy with fatal congenital infection. *Pediatrics* 1981; **67**(1): 89-94.
77. Laartz BW, Narvarte HJ, Holt D, Larkin JA, Pomputius WF, 3rd. Congenital tuberculosis and management of exposures in a neonatal intensive care unit. *Infection control and hospital epidemiology : the official journal of the Society of Hospital Epidemiologists of America* 2002; **23**(10): 573-9.
78. Aaby P, Benn CS. Non-specific and sex-differential effects of routine vaccines: what evidence is needed to take these effects into consideration in low-income countries? *Human vaccines* 2011; **7**(1): 120-4.

79. Fine PE, Williams TN, Aaby P, et al. Epidemiological studies of the 'non-specific effects' of vaccines: I--data collection in observational studies. *Tropical medicine & international health : TM & IH* 2009; **14**(9): 969-76.
80. Jahn A, Floyd S, Mwinuka V, et al. Ascertainment of childhood vaccination histories in northern Malawi. *Tropical medicine & international health : TM & IH* 2008; **13**(1): 129-38.
81. Cutts FT, Rodrigues LC, Colombo S, Bennett S. Evaluation of factors influencing vaccine uptake in Mozambique. *International journal of epidemiology* 1989; **18**(2): 427-33.
82. Meeting of Global Advisory Committee on Vaccine Safety, 18-19 June 2008. *Releve epidemiologique hebdomadaire / Section d'hygiene du Secretariat de la Societe des Nations = Weekly epidemiological record / Health Section of the Secretariat of the League of Nations* 2008; **83**(32): 287-92.
83. Shah V, Ohlsson A. Venepuncture versus heel lance for blood sampling in term neonates. *Cochrane database of systematic reviews* 2007; (4): CD001452.
84. Kapellou O. Blood sampling in infants (reducing pain and morbidity). *Clinical evidence* 2011; **2011**.
85. Howie SR. Blood sample volumes in child health research: review of safe limits. *Bulletin of the World Health Organization* 2011; **89**(1): 46-53.
86. Sartono E, Lisse IM, Terveer EM, et al. Oral polio vaccine influences the immune response to BCG vaccination. A natural experiment. *PloS one* 2010; **5**(5): e10328.
87. Lund N, Andersen A, Monteiro I, Aaby P, Benn CS. No effect of oral polio vaccine administered at birth on mortality and immune response to BCG. A natural experiment. *Vaccine* 2012; **30**(47): 6694-9.

Appendix 1.

LSHTM Safety Reporting Procedures for Drug Trials

7.1 DEFINITIONS

Adverse Event (AE): any untoward medical occurrence in a patient or clinical trial subject administered a medicinal product and which does not necessarily have a causal relationship with this treatment. *An AE can therefore be any unfavourable and unintended sign (including an abnormal laboratory finding), symptom, or disease temporally associated with the use of an investigational medicinal product (IMP), whether or not considered related to the IMP.*

Adverse Reaction (AR): all untoward and unintended responses to an IMP related to any dose administered. *All AEs judged by either the reporting investigator or the sponsor as having reasonable causal relationship to a medicinal product qualify as adverse reactions. The expression reasonable causal relationship means to convey in general that there is evidence or argument to suggest a causal relationship.*

Unexpected Adverse Reaction: an AR, the nature or severity of which is not consistent with the applicable product information (eg investigator's brochure for an unapproved investigational product or summary of product characteristics (SmPC) for an authorised product*). When the outcome of the adverse reaction is not consistent with the applicable product information this adverse reaction should be considered as unexpected. Side effects documented in the SmPC which occur in a more severe form than anticipated are also considered to be unexpected.*

Serious Adverse Event (SAE) or **Serious Adverse Reaction:** any untoward medical occurrence or effect that at any dose

- **Results in death**
- **Is life-threatening** – *refers to an event in which the subject was at risk of death at the time of the event; it does not refer to an event which hypothetically might have caused death if it were more severe*
- **Requires hospitalisation, or prolongation of existing inpatients' hospitalisation**
- **Results in persistent or significant disability or incapacity**
- **Is a congenital anomaly or birth defect**

Medical judgement should be exercised in deciding whether an AE/AR is serious in other situations. Important AE/ARs that are not immediately life-threatening or do not result in death or hospitalisation but may jeopardise the subject or may require intervention to prevent one of the other outcomes listed in the definition above, should also be considered serious.

Suspected Unexpected Serious Adverse Reaction (SUSAR): any suspected adverse reaction related to an IMP that is both unexpected and serious.

7.2 CAUSALITY

Most adverse events and adverse drug reactions that occur in this study, whether they are serious or not, will be expected treatment-related toxicities due to the drugs used in this study. The assignment of the causality should be made by the investigator responsible for the care of the participant using the definitions in the table below.

If any doubt about the causality exists the local investigator should inform the study coordination centre who will notify the Chief Investigators. The pharmaceutical companies and/or other clinicians may be asked to advise in some cases.

In the case of discrepant views on causality between the investigator and others, all parties will discuss the case. In the event that no agreement is made, the MHRA will be informed of both points of view.

**Relationship**	**Description**
**Unrelated**	There is no evidence of any causal relationship
**Unlikely**	There is little evidence to suggest there is a causal relationship (e.g. the event did not occur within a reasonable time after administration of the trial medication). There is another reasonable explanation for the event (e.g. the participant's clinical condition, other concomitant treatment).
**Possible**	There is some evidence to suggest a causal relationship (e.g. because the event occurs within a reasonable time after administration of the trial medication). However, the influence of other factors may have contributed to the event (e.g. the participant's clinical condition, other concomitant treatments).
**Probable**	There is evidence to suggest a causal relationship and the influence of other factors is unlikely.
**Definitely**	There is clear evidence to suggest a causal relationship and other possible contributing factors can be ruled out.
**Not assessable**	There is insufficient or incomplete evidence to make a clinical judgement of the causal relationship.

7.3 REPORTING PROCEDURES

All adverse events should be reported. Depending on the nature of the event the reporting procedures below should be followed. Any questions concerning adverse event reporting should be directed to the study coordination centre in the first instance. A flowchart is shown below to aid in the reporting procedures.

7.3.1 Non serious Adverse Reactions (ARs)/Adverse Events (AEs)

All such events, whether expected or not, should be recorded in the toxicity section of the relevant case report form and sent to the study coordination centre within one month of the form being due.

7.3.2 Serious Adverse Reactions (SARs)/Serious Adverse Events (SAEs)

Fatal or life threatening Serious Adverse Events (SAEs), Serious Adverse Reactions (SARs) and Suspected Unexpected Serious Adverse Reactions (SUSARs) should be reported on the day that the local site is aware of the event. The SAE form asks for nature of event, date of onset, severity, corrective therapies given, outcome and causality (i.e. unrelated, unlikely, possible, probably, definitely). The responsible investigator should sign the causality of the event. Additional information should be sent within 5 days if the reaction has not resolved at the time of reporting.

SAEs

An SAE form should be completed and faxed to the study coordination centre for all SAEs within 24 hours. However, hospitalisations for elective treatment of a pre-existing condition, or for accidental injury unrelated to the trial do not need reporting as SAEs.

SUSARs

In the case of serious, unexpected and related adverse events, the staff at the site should:

> Complete the SAE case report form & send it immediately (within 24 hours, preferably by fax), signed and dated to the study coordination centre together with relevant treatment forms and anonymised copies of all relevant investigations.

Or

> Contact the study coordination centre by phone and then send the completed SAE form to the study coordination centre within the following 24 hours as above.

The study coordination centre will notify the local regulatory authority and ethics committee of all SUSARs occurring during the study according to the following timelines; fatal and life-threatening within 7 days of notification and non-life threatening within 15 days. All investigators will be informed of all SUSARs occurring throughout the study.

Local investigators should report any SUSARs and /or SAEs as required by their Local Research Ethics Committee and/or Research & Development Office.

Contact details for reporting SAEs and SUSARs
Fax: To be added, attention: Dr Sarah Prentice
Please send SAE forms to: To be added
Tel: To be added (Mon to Fri 09.00 – 17.00)

Flowchart for safety reporting

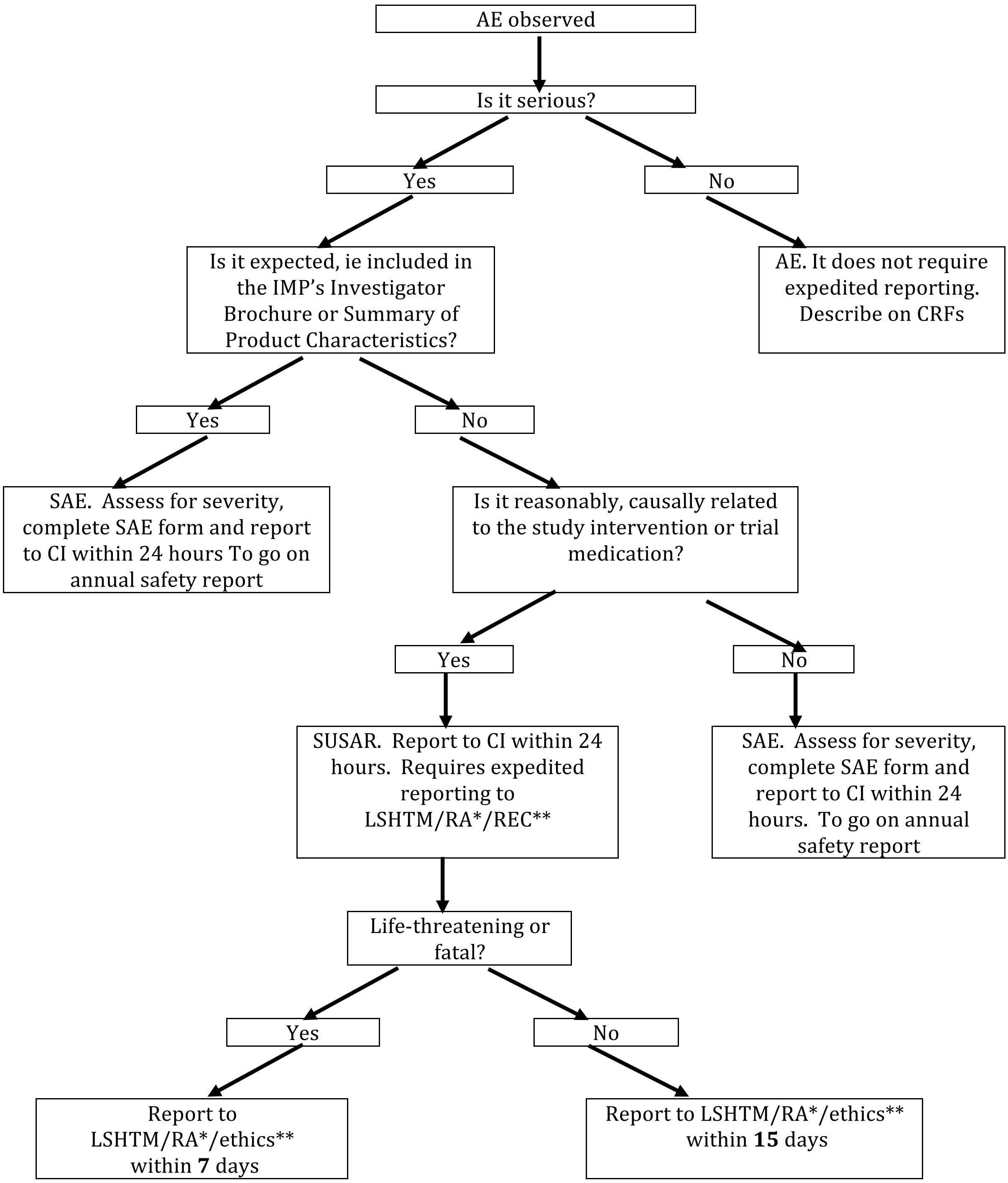

** RA: Regulatory Authority, eg MHRA in the UK*

*** And report to local regulatory authority and ethics committee as required by each country*
